# The protective PLCγ2-P522R variant mitigates Alzheimer’s disease-associated pathologies by enhancing beneficial microglial functions

**DOI:** 10.1186/s12974-025-03387-6

**Published:** 2025-03-05

**Authors:** Mari Takalo, Heli Jeskanen, Taisia Rolova, Inka Kervinen, Marianna Hellén, Sami Heikkinen, Hennariikka Koivisto, Kimmo Jokivarsi, Stephan A. Müller, Esa-Mikko Koivumäki, Petra Mäkinen, Sini-Pauliina Juopperi, Roosa-Maria Willman, Rosa Sinisalo, Dorit Hoffmann, Henna Jäntti, Michael Peitz, Klaus Fließbach, Teemu Kuulasmaa, Teemu Natunen, Susanna Kemppainen, Pekka Poutiainen, Ville Leinonen, Tarja Malm, Henna Martiskainen, Alfredo Ramirez, Annakaisa Haapasalo, Stefan F. Lichtenthaler, Heikki Tanila, Christian Haass, Juha Rinne, Jari Koistinaho, Mikko Hiltunen

**Affiliations:** 1 Institute of Biomedicine, University of Eastern Finland, Kuopio, Finland; 2 Neuroscience Center, Helsinki Institute of Life Science (HiLIFE), University of Helsinki, Helsinki, Finland; 3 A. I. Virtanen Institute for Molecular Sciences, University of Eastern Finland, Kuopio, Finland; 4 German Center for Neurodegenerative Diseases (DZNE Munich), Munich, Germany; 5 Neuroproteomics, School of Medicine and Health, Klinikum Rechts Der Isar, Technical University of Munich, Munich, Germany; 6 Turku PET Centre, University of Turku, Turku, Finland; 7 Turku PET Centre, Turku University Hospital, Turku, Finland; 8 Institute of Reconstructive Neurobiology, University of Bonn Medical Faculty & University Hospital Bonn, Bonn, Germany; 9 Cell Programming Core Facility, University of Bonn Medical Faculty, Bonn, Germany; 10 Department of Old Age Psychiatry and Cognitive Disorders, University of Bonn Medical Center, Bonn, Germany; 11 German Center for Neurodegenerative Diseases (DZNE Bonn), Bonn, Germany; 12 Diagnostic Imaging Center, Kuopio University Hospital, Kuopio, Finland; 13 Department of Neurosurgery, Kuopio University Hospital, Kuopio, Finland; 14 Institute of Clinical Medicine – Neurosurgery, University of Eastern Finland, Kuopio, Finland; 15 Division of Neurogenetics and Molecular Psychiatry, Department of Psychiatry and Psychotherapy, Medical Faculty, University of Cologne, Cologne, Germany; 16 Department of Old Age Psychiatry and Cognitive Disorders, University Hospital Bonn, University of Bonn, Bonn, Germany; 17 Glenn Biggs Institute for Alzheimer’s & Neurodegenerative Diseases, University of Texas Health Sciences Center, San Antonio, TX USA; 18 Cologne Excellence Cluster on Cellular Stress Responses in Aging-Associated Disease (CECAD), University of Cologne, Cologne, Germany; 19 Munich Cluster for Systems Neurology (SyNergy), Munich, Germany; 20 Metabolic Biochemistry, Biomedical Centre (BMC), Faculty of Medicine, Ludwig-Maximilians-Universität München, Munich, Germany; 21 InFLAMES Research Flagship, University of Turku, Turku, Finland; 22 Drug Research Program, Division of Pharmacology and Pharmacotherapy, University of Helsinki, Helsinki, Finland

**Keywords:** Alzheimer’s disease, β-amyloid pathology, Microglia, Lipid droplets, Phospholipase C gamma 2, PLCγ2-P522R variant

## Abstract

**Background:**

Phospholipase C gamma 2, proline 522 to arginine (PLCγ2-P522R) is a protective variant that reduces the risk of Alzheimer’s disease (AD). Recently, it was shown to mitigate β-amyloid pathology in a 5XFAD mouse model of AD. Here, we investigated the protective functions of the PLCγ2-P522R variant in a less aggressive APP/PS1 mouse model of AD and assessed the underlying cellular mechanisms using mouse and human microglial models.

**Methods:**

The effects of the protective PLCγ2-P522R variant on microglial activation, AD-associated β-amyloid and neuronal pathologies, and behavioral changes were investigated in PLCγ2-P522R knock-in variant mice crossbred with APP/PS1 mice. Transcriptomic, proteomic, and functional studies were carried out using microglia isolated from mice carrying the PLCγ2-P522R variant. Finally, microglia-like cell models generated from human blood and skin biopsy samples of PLCγ2-P522R variant carriers were employed.

**Results:**

The PLCγ2-P522R variant decreased β-amyloid plaque count and coverage in female APP/PS1 mice. Moreover, the PLCγ2-P522R variant promoted anxiety in these mice. The area of the microglia around β-amyloid plaques was also increased in mice carrying the PLCγ2-P522R variant, while β-amyloid plaque-associated neuronal dystrophy and the levels of certain cytokines, including IL-6 and IL-1β, were reduced. These alterations were revealed through [18F]FEPPA PET imaging and behavioral studies, as well as various cytokine immunoassays, transcriptomic and proteomic analyses, and immunohistochemical analyses using mouse brain tissues. In cultured mouse primary microglia, the PLCγ2-P522R variant reduced the size of lipid droplets. Furthermore, transcriptomic and proteomic analyses revealed that the PLCγ2-P522R variant regulated key targets and pathways involved in lipid metabolism, mitochondrial fatty acid oxidation, and inflammatory/interferon signaling in acutely isolated adult mouse microglia and human monocyte-derived microglia-like cells. Finally, the PLCγ2-P522R variant also increased mitochondrial respiration in human iPSC-derived microglia.

**Conclusions:**

These findings suggest that the PLCγ2-P522R variant exerts protective effects against β-amyloid and neuronal pathologies by increasing microglial responsiveness to β-amyloid plaques in APP/PS1 mice. The changes observed in lipid/fatty acid and mitochondrial metabolism revealed by the omics and metabolic assessments of mouse and human microglial models suggest that the protective effects of the PLCγ2-P522R variant are potentially associated with increased metabolic capacity of microglia.

**Supplementary Information:**

The online version contains supplementary material available at 10.1186/s12974-025-03387-6.

## Background

Phospholipase C gamma 2 (PLCγ2) is an important enzyme that is involved in the regulation of multiple cellular signaling pathways. PLCγ2 plays crucial roles in various cellular processes, including signal transduction, cell differentiation, cell proliferation, survival, and immune responses. Upon activation, PLCγ2 specifically converts phosphatidylinositol 4,5-bisphosphate (PIP2) into the second messengers inositol trisphosphate (IP3) and diacylglycerol (DAG). In turn, these trigger intracellular calcium release, activate downstream signaling pathways, and lead to various cellular responses. PLCγ2 is particularly important in immune cells, where it is activated by antigens binding to the surface receptors of immune cells [1].

In the brain, PLCγ2 is expressed primarily in microglia, the resident immune cells of the brain [2, 3]. Microglia play a key role in surveillance; tissue homeostasis; and the response to injury, infection, and accumulation of toxic metabolites, including β-amyloid [4]. In the context of Alzheimer’s disease (AD), activated microglia limit disease pathology by clearing and/or compacting β-amyloid and protecting vulnerable neurons from its toxic effects [5]. Conversely, extensive microglial activation promotes chronic neuroinflammation, which causes neuronal damage and thus is a major player in AD pathogenesis alongside β-amyloid aggregation and hyperphosphorylated tau deposition [4].

In microglia, PLCγ2 functions as a key downstream effector for triggering receptor expressed on myeloid cells 2 (TREM2) and Toll-like receptors (TLRs) [6, 7]. TREM2 is a microglia-specific lipid sensor that initiates an intracellular signaling cascade upon ligand binding, leading to changes in gene transcription, cell proliferation, cell metabolism, phagocytosis, and survival. Genetic depletion of either TREM2 or PLCγ2 similarly impairs lipid processing and mitochondrial function in human iPSC-derived microglia (iMGL) [6, 8]. The impairment of cellular metabolism may be the driver of a dysfunctional and senescent phenotype in microglia during aging and during β-amyloid accumulation [9, 10].

Recent genetic studies support a strong link between late-onset AD (LOAD) and *PLCG2,* the gene encoding PLCγ2. A rare gain-of-function variant (rs72824905, C > G, p.P522R) was shown to reduce the risk of AD [2]. The same variant was also suggested to have other beneficial effects on brain health. The risks of dementia with Lewy bodies and frontotemporal dementia were shown to be reduced in individuals carrying the PLCγ2-P522R variant, and the protective variant has been found to be highly expressed among old (100+ years) individuals with well-preserved cognitive functions [11]. Furthermore, the rate of cognitive decline was reduced in patients with mild cognitive impairment carrying the PLCγ2-P522R variant compared to those not carrying the variant, suggesting that the PLCγ2-P522R variant has universally beneficial effects on cognition and brain health upon aging [12]. In addition, other gain- and loss-of-function variants in *PLCG2* have recently been linked to a decreased or an increased risk of AD, respectively, which further supports the key role of PLCγ2 in AD and general brain health [13–15].

PLCγ2-P522R is a functional hypermorph that mildly increases PLCγ2 enzyme activity [3, 6, 7]. We previously showed that the PLCγ2-P522R variant increases microglial activation in adult C57BL/6J mice and enhances the acute inflammatory response and increases the survival of mouse bone marrow-derived macrophages upon treatment with lipopolysaccharide (LPS) [7]. To date, only a few studies have underlined the role of the PLCγ2-P522R variant in AD pathogenesis. A study utilizing AD model 5XFAD mice transplanted with human PLCγ2-P522R iMGLs reported that the PLCγ2-P522R variant has no influence on β-amyloid pathology [16]. In contrast, PLCγ2-P522R was shown to promote microglia-mediated phagocytosis of β-amyloid, leading to more compacted plaque morphology, reduced neuronal damage, and improved working memory in 5XFAD model mice with endogenous microglia carrying the PLCγ2-P522R variant [17]. Importantly, very similar effects have been reported for agonistic anti-TREM2 antibodies, which are designed to enhance microglial functions in AD patients [8, 18]. While these studies emphasize the importance of TREM2-PLCγ2-driven microglia in mitigating AD-related pathologies and highlight PLCγ2 as a plausible disease-modifying therapeutic target in AD, more data gathered from different models are needed to comprehensively understand the role of PLCγ2-P522R in AD. This study aimed to investigate the effects of the protective PLCγ2-P522R variant on microglial responses and AD-related β-amyloid and neuronal pathologies as well as behavioral changes for the first time in a less aggressive APP/PS1 AD mouse model. In addition, mouse primary microglial and human microglial models were utilized to assess the molecular and cellular mechanisms underlying the protective effects of the PLCγ2-P522R variant.

## Materials and methods

### Study design

In this study, the APPswe/PS1dE9 transgenic mouse line (later simply APP/PS1) [19] was crossbred with the PLCγ2-P522R knock-in (KI) mouse line [7] to investigate the effects of the PLCγ2-P522R variant on AD-related β-amyloid and associated neuronal pathologies, microglial activation, and behavioral changes. Female and male mice at the ages of 7 and 13 months were subjected to TSPO-PET imaging with the [18F]FEPPA ligand to monitor microglial activation [7]. The mice were sacrificed at the age of 13 months, and half of the brain was cut into coronal sections and used for immunohistochemical analyses, whereas the other half was dissected into regional blocks and utilized for biochemical assays. The entorhinal cortex and hippocampus were subjected to immunohistochemical analyses, while the temporo-occipital cortex and hippocampus of the same mice were subjected to biochemical analyses. The whole brains of a subset of 13-month-old mice were used for CD11b+ microglia isolation and the isolated CD11b+ cells were used for RNA sequencing studies. Behavioral testing was conducted on 12- to 13-month-old mice. All immunohistochemical, biochemical, and behavioral analyses were conducted in female mice.

CD11b+ microglia acutely isolated from 13-month-old PLCγ2-P522R KI and wild-type (WT) mice and cultured primary microglia isolated from 0–3-day-old pups under different conditions to induce cellular stress were subjected to global transcriptomic and proteomic analyses as well as functional studies. Transcriptomic analyses were conducted in blood monocyte-derived microglia (MDMi) isolated from PLCγ2-P522R variant carriers and matched controls to study how well the findings translated to human cells. Induced pluripotent stem cell-derived microglia (iMGL) homozygous for the PLCγ2-P522R variant with either an *APOE3/3* or *APOE4/4* genetic background and isogenic control lines were used to functionally assess the key findings.

### Mouse lines

The APP/PS1 line in the C57BL/6J background was used as a model for AD [19]. This mouse line coexpresses a single transgene, a chimeric mouse/human APP695 harboring the Swedish K670M/N671L mutation (Mo/Hu APPswe), and a human PS1 with exon 9 deletion (PS1dE9) under the mouse prion protein promoter. This line was crossbred with a PLCγ2-P522R KI mouse line generated using CRISPR/Cas9-assisted gene editing on a C57Bl/6J background, as we reported previously [7]. WT (C57BL/6J) mice were used as controls in in vitro experiments. The mice were kept in an environment with a constant temperature of 22 ± 1 °C and 50–60% humidity. The lights were on from 07:00–19:00, and food and water were available ad libitum. All mouse studies were carried out in accordance with the guidelines of the European Community Council Directives 86/609/EEC and approved by the Animal Experiment Board of Finland/Regional State Administrative Agency and Project Authorization Board (ESAVI 21203–2019, EKS-004–2019).

### Mouse genotyping

Genomic DNA was purified from ear biopsies by incubating the samples with 50 mM NaOH for 1 h at 95 °C and neutralizing them with 1 M Tris, pH 8.0. Genotyping of the PLCγ2-P522R locus was carried out with SNP genotyping. Ten nanograms of DNA were mixed with TaqMan Genotyping master mix (Applied Biosystems) and a TaqMan SNP genotyping assay (Applied Biosystems) targeted to trinucleotide variation, leading to the substitution of proline 522 with arginine [7]. The reaction mixture was amplified for 10 s at 95 °C, 15 s at 95 °C, and 60 s at 60 °C for 40 cycles and measured with a LightCycler® 480 II (Roche).

To confirm the expression of the *APP* and *PSEN1* genes, loci harboring the human *PSEN1* and mouse prion protein promoters were amplified using the following primers: 5'-CCTCTTTGTGACTATGTGGACTGATGTCGG-3', 5'-GTGGATACCCCCTCCCCCAGCCTAGACC-3', and 5'-CAGGTGGTGGAGCAAGATG-3'. Touchdown PCR was performed using the following program: 3 min at 94 °C; 30 s at 94 °C, 1 min at 62–58 °C, and 2 min at 72 °C, for two cycles at each temperature; 30 s at 94 °C, 1 min at 57 °C, and 2 min at 72 °C for 25 cycles; and 5 min at 72 °C. The 1300 bp (*PSEN1*) and 750 bp (internal control) bands were detected with agarose gel electrophoresis.

### Behavioral studies

Starting at the age of 12 months, the mice were subjected to a behavioral test battery. The mice were weighed before testing began.

Spontaneous explorative activity was assessed with an automated activity monitor (TruScan, Coulbourn Instruments, Whitehall, PA, USA) based on infrared photobeam detection. The system consisted of an observation cage with white plastic walls (26 cm × 26 cm × 39 cm) and two frames of photo detectors for monitoring horizontal and vertical activity. The test cage was cleaned with 70% ethanol before each mouse to remove odor traces. The following parameters were measured during a 10-min session: ambulatory distance (gross horizontal locomotion), rearing time, and time in the area center vs. periphery.

A light‒dark box with an electric grid floor in the dark chamber was used to test spontaneous fear towards the illuminated chamber and for foot-shock reinforced passive avoidance. On Day 1, the mouse was first placed in the illuminated chamber with free access to the dark chamber through a narrow opening, and the time spent in the light and dark chambers was recorded for 5 min. Thereafter, the mouse was placed again in the illuminated chamber for 30 s with the entrance to the dark chamber closed. The slide door separating the chambers was then opened. As soon as the mouse entered the dark side (cutoff 3 min), the sliding door was closed, and a mild foot shock was delivered (2 × 2 s at 0.30 mA). The mouse was then returned to its home cage. On Day 3 (48 h later), the mouse was again placed in the illuminated chamber with the sliding door open, and the time to enter the dark chamber was recorded as an index for fear memory. Finally, the mouse was confined to the dark chamber, and the pain threshold to electric foot shock was determined by gradually increasing the current on the grid floor until the mouse reacted.

Motor coordination and balance were tested using an automated rotarod device (Uno Bacile, Italy). Each mouse was first familiarized with the rotating rod twice for 2 min by allowing it to rotate at a constant speed of 4 rpm. On a separate day, the mouse was tested twice, with the speed of the rod increasing stepwise from 4 to 40 rpm. The cutoff time was 6 min. The trial with longer latency to fall was recorded.

The pain threshold was tested using a hotplate with surface temperatures of 47 °C and 51 °C. The time to lick a paw or jump was recorded until a cutoff time of 60 s. Each mouse was tested three times at both temperatures (lower temperature first), with 1 h between the test sessions. The average reaction time at each temperature was recorded.

Spatial learning and memory were assessed using the Morris water maze (MWM) test. The test was conducted in a white circular wading pool (diameter of 120 cm), and a transparent platform (14 cm × 14 cm) was submerged underneath the water. The pool was open to landmarks in the room. The water temperature was 20 ± 0.5 °C. The testing was preceded by two training days with a guiding alley to the platform. During the acquisition phase (Days 1–5), the location of the hidden platform was kept constant, and the start position varied between four different locations at the pool edge. Each mouse was placed in the water with its nose pointing toward the pool wall. If the mouse failed to find the escape platform within 60 s, it was placed on the platform for 10 s by the experimenter. The acquisition phase consisted of five daily trials with a 10 min intertrial interval. On Day 5, the search bias was tested in a 60-s probe trial (the 5th trial) without the platform. The mouse was video tracked, and a video analysis program (EthoVision, Noldus, The Netherlands) was used to calculate the escape latency, swimming speed, path length and time at the pool periphery (10 cm from the wall) and in the platform zone (diameter of 30 cm).

### PET imaging

[18F] FEPPA-PET imaging was conducted on female and male mice at the ages of 7 and 13 months. [18F]FEPPA is a specific positron emission tomography (PET) ligand for the mitochondrial translocator protein 18 kDa (TSPO) that is highly expressed in activated microglia [20]. The mice were anesthetized with isoflurane (1.5% with N2/O2 70%/30% through a nose cone) and placed on a heated animal holder (Équipement Vétérinaire Minerve, Esternay, France) on the scanner bed in a prone position and secured with tape to prevent movement during scanning. For the 7-month timepoint, the mice were imaged using a dedicated PET scanner (Inveon DPET, Siemens Healthcare) and immediately afterward with a CT scanner (Flex SPECT/CT, Gamma Medica, Inc.) for anatomical reference images using the same animal holder. For the 13-month timepoint, imaging was performed with a preclinical PET/MRI scanner (MR Solutions Ltd., Surrey, UK). The change in scanners was due to unexpected technical problems with the dedicated PET scanner. Dynamic imaging for 70 min was started at the time of activity. 18F-FEPPA (8.0 ± 2.0 MBq) was injected as a slow bolus over a period of 30 s through the tail vein. PET data were gathered in list-mode form and corrected for deadtime, randomness, scatter and attenuation. The data were reconstructed using the scanner manufacturers' software with a 2D- (7 mo) or 3D- (13 mo) ordered-subsets expectation maximization (OSEM) algorithm. Regions of interest (ROIs) were drawn for the whole brain (excluding the cerebellum and olfactory bulb), frontal cortex, pons, hippocampus and cerebellum. The ROIs were drawn using Carimas 2.10 software (Turku PET Centre, Finland). The final uptake values (percent of injected dose per ml of tissue) were the average values from 20 to 70 min from the ligand injection.

### Tissue collection and sample preparation

At the age of 13 months, terminal anesthesia was administered as intraperitoneal injection of 60 mg/ml pentobarbital, after which mice were transcardially perfused with ice-cold phosphate-buffered saline (DPBS, #17-512F, BioNordica) for 5 min to rinse blood from the brain. The brain was quickly removed and placed on ice. One brain hemisphere was immersed in 4% paraformaldehyde (PFA) in phosphate buffer (0.05 M, pH 7.6) for 24 h at 4 °C, followed by incubation in 30% sucrose in DPBS overnight. The fixed tissue was stored in antifreeze (0.5 M sucrose, 37.5% (v/v) ethylene glycol, 0.03 M phosphate buffer (pH 7.6), 0.004 M sodium azide) at − 20 °C until it was cut into 35 µm-thick coronal sections using a freezing sliding microtome.

The temporo-occipital cortex and hippocampus were dissected from the other hemisphere and snap-frozen in liquid nitrogen. The tissue pieces were mechanically homogenized in 300 µl of ice-cold DPBS. Fifty microliters of the lysate was mixed with 500 µl of TRIzol reagent (#T9424, Sigma‒Aldrich) and used for RNA extraction (below). The remaining lysate was supplemented with 1:100 HALT™ EDTA-free protease (#87785, Thermo Scientific) and HALT^™^ phosphatase (#78420, Thermo Scientific) inhibitor cocktails. Proteins were extracted by incubating the lysate on ice for 30 min with Tissue Protein Extraction Reagent (T-PER, #78510, Thermo Scientific), and the supernatant was collected after centrifugation for 10 min at 10 000 × g at 4 °C. The fraction of the PBS lysate was ultracentrifuged at 100 000 × g for 2 h at 4 °C, and the supernatant containing the soluble fraction was collected in clean tubes. The pellets containing the insoluble fraction were solubilized in 5 M guanidine-HCl by vortexing for 3 h at room temperature. The samples were subsequently centrifuged at 10 000 × g for 10 s. The protein concentrations were measured using a BCA protein assay kit (#23225, Pierce).

### Western blotting

Total protein lysates (15–20 μg) were supplemented with NuPAGE LDS sample buffer (#NP0007, Invitrogen) containing β-mercaptoethanol and separated via SDS‒PAGE via NuPAGE 4–12% Bis–Tris Midi protein gels (#WG1402BOX, Invitrogen). Proteins were subsequently transferred to polyvinylidene difluoride (PVDF) membranes (#IB24001, Invitrogen) using the iBlot 2 Dry Blotting System (Invitrogen). Unspecific antibody binding was blocked by incubating the blots in 5% nonfat milk or 5% bovine serum albumin (BSA, #A9647, Sigma‒Aldrich) in 1 × Tris-buffered saline with 0.1% Tween 20 (TBST) for 1 h at room temperature. Proteins were detected from the blots using the following primary antibodies diluted in 1 × TBST and incubated overnight at 4 °C: mouse anti-β-amyloid 1:1000 (6E10, #SIG-39320, BioLegend), rabbit anti-sAPPβ 1:100 (#18957, IBL America), mouse anti-APP N-terminal 1:1000 (22C11, #MAB348, Merck), rabbit anti-APP C-terminal 1:1000 (#A8717, Merck), and mouse anti-GAPDH 1:10 000 (#ab8226, Abcam). The blots were subsequently incubated with sheep anti-mouse 1:5000 (#NA931V, Cytiva) or donkey anti-rabbit 1:5000 (#NA934V, Cytiva) horseradish peroxidase (HRP)-conjugated secondary antibodies in 1 × TBST for 1 h at room temperature. Enhanced chemiluminescence Prime (#RPN2232, Cytiva) and Select (#RPN2235, Cytiva) Western blotting detection reagents were used to detect the protein bands. SeeBlue Plus2 Prestained Standard (#LC5925, Invitrogen) was used to estimate the size of the detected bands. The blots were imaged with a ChemiDoc MP system (Bio-Rad), and the images were quantified using the ImageLab (Bio-Rad) software. Results are shown as percentage of A+/P^wt/wt^ group.

### Aβ40, Aβ42, sTREM2, and APOE ELISA

Aβ40 and Aβ42 levels in the soluble and insoluble lysates were determined with Amyloid β (40) Human/Rat (#294–64701, FUJIFILM, Wako) and High-Sensitivity Amyloid β (42) Human/Rat (#292–64501, FUJIFILM, Wako) ELISA Kits following the manufacturers’ instructions. Aβ levels were normalized to the total protein concentration within each sample.

Soluble and insoluble APOE were measured in lysates using an Apolipoprotein E (APOE) Mouse ELISA Kit (#ELK2007, Gentaur) according to the manufacturer’s instructions. APOE levels were normalized to the total protein concentration within the same sample.

For sTREM2 measurement from the respective lysates, a 96-well Costar Assay Plate (9018, Corning) was coated with 500 ng/ml human/mouse anti-TREM2 antibody (#MAB17291-100, R&D Systems) in 0.5 M carbonate-bicarbonate buffer (pH 9.6, #C3041, Sigma‒Aldrich) and incubated at 4 °C overnight. The wells were subsequently washed 3 times with 0.05% Tween 20 (#93773-250G, Sigma‒Aldrich) in DPBS and blocked with 1% Block Ace (#BUF029, Bio-Rad) in DPBS for 4 h at room temperature. The wells were washed once before adding Recombinant Mouse TREM2 Protein in concentrations 12.21–781.25 pg/ml (#50,149-M08H, Sino Biological, Inc.), and the samples were diluted in assay buffer (1% BSA (#A9647, Sigma‒Aldrich) and 0.05% Tween 20 in DPBS). The standards and samples were incubated at 4 °C overnight. The wells were washed five times following incubation with 66.67 pg/ml mouse TREM2 biotinylated antibody (#BAF1729, R&D Systems) for 1 h at room temperature, after which the wells were washed 3 times. Streptavidin poly-HRP40 (#20102011, Fitzgerald) diluted 1:3000 in assay buffer was added, and the mixture was incubated for 1 h at room temperature in the dark, after which the wells were washed 5 times. 3,3′,5,5′-Tetramethylbenzidine Liquid Substrate (TMB, Super Slow, #T5569, Sigma‒Aldrich) was added, and the mixture was incubated for 15 min at room temperature. The reaction was stopped with 1 M H_3_PO_4,_ and the absorbances were measured with a plate reader (Tecan Infinite, 450 nm).

### Mesoscale inflammatory marker assay

The levels of inflammatory cytokines and transforming growth factor beta (TGF-β) 1–3 in the cortical and hippocampal lysates were determined with an MSD mouse V-plex Proinflammatory Panel 1 (#K15048, Mesoscale) and a U-plex TGFβ Combo Panel (#K15242K, Mesoscale), respectively, according to the manufacturer’s instructions. The results were normalized to the total protein concentration in each sample.

### Immunohistochemical staining of mouse brain sections

Three to four brain sections between the bregma − 3.1 mm and − 3.5 mm coronal planes according to Paxinos and Franklin’s Mouse Brain in Stereotaxic Coordinates (2001) were selected from each mouse for every staining. Before staining, the sections were incubated in sodium phosphate buffer overnight at room temperature. The next day, the sections were pretreated with 0.05 M citrate solution (pH 6.0) for 30 min at 80 °C and cooled in sodium phosphate buffer for 20 min. To visualize β-amyloid plaques, the sections were incubated with 0.1 mM X-34 (#SML1954, Sigma‒Aldrich) for 1 h at room temperature in a solution containing 60% DPBS and 40% EtOH. The sections were then rinsed three times with 60% DPBS + 40% EtOH followed by washing with TBST three times for 5 min. Nonspecific antibody binding was blocked with 3% BSA in TBST, after which the sections were incubated with the following primary antibodies at 4 °C overnight: a rabbit anti-β-amyloid antibody (1:1000, 6E10, #SIG-39320, BioLegend) to detect diffuse β-amyloid, a mouse anti-APP N-terminal antibody (1:2000, 22C11, #MAB348, Merck) to visualize dystrophic neurites, a rabbit anti-IBA1 antibody (1:1000 #019–19741, FUJIFILM Wako) to detect microglia, a rabbit anti-GFAP antibody (1:500, #Z0334, Dako) to detect astrocytes, and a goat anti-APOE antibody (1:1000, #178479, Merck). After being washed three times for 5 min each with TBST at room temperature, the sections were incubated with Alexa Fluor^™^ 568 donkey anti-mouse (#A-10037, Thermo Scientific), Alexa Fluor^™^ 488 goat anti-rabbit (#A-11008, Invitrogen), Alexa Fluor™ 488 donkey anti-rabbit (#A-21206, Thermo Scientific), Alexa Fluor™ 568 goat anti-rabbit (#A-11036, Invitrogen), and Alexa Fluor™ 568 donkey anti-goat (#A-11057, Thermo Scientific) secondary antibodies for 1 h at room temperature. The sections were again washed three times for 5 min and then mounted with Vectashiled HardSet Antifade Mounting Medium (#H-1400, Vector Laboratories) on gelatin-coated slides. Control sections without primary antibody treatment were processed simultaneously.

### Fluorescence microscopy and image analysis

For X-34-positive β-amyloid plaque coverage analysis, the slices were imaged with a Leica Thunder Imager 3D Tissue slide scanner equipped with a K5-14400955 camera and an HC PL FLUOTAR 20x (NA 0.5) DRY objective (Leica Microsystems). Z-stack images were taken with 19 layers with a total distance of 27 μm. The whole entorhinal cortex and hippocampus were scanned, stitched, and subjected to automated Thunder image processing computational clearance. All the stained samples, which included more than one fluorophore, were imaged with a Zeiss Axio Observer inverted microscope with a Zeiss LSM 800 confocal module (Carl Zeiss Microimaging GmbH) with a 20 × (NA 0.5) objective. A Plan-Apochromat 63 × (NA 1.4) objective was used for imaging 6E10 and IBA1 for Imaris-based colocalization analysis. Three 1024 × 1024-pixel fields from both brain areas were taken via the Z-stack imaging method 15–19 layers with a total distance of 22.5–27 μm. All laser, light, and detector settings were kept constant for all immunostained samples. Images were taken by investigators who were blinded to the mouse groups.

Quantitative image analysis was carried out with Fiji (ImageJ, version 1.53c) software. Maximum intensity projections were generated for each channel, and the rolling ball algorithm (radius 50) and Gaussian blurring (sigma 2) were applied to subtract the background and remove noise, respectively. The following automatic thresholding methods were used to create a mask for each stain: Otsu for X-34, Moments for 22C11 and IBA1, and Li for GFAP and APOE. For Leica Thunder Imager 3D tissue slide scanner images, the entorhinal cortex and hippocampal ROIs were manually drawn for each image. The X-34-positive β-amyloid plaque area and the number of plaques in the entorhinal cortex and hippocampus were subsequently normalized to the area of the respective ROI. For the analysis of IBA1, GFAP, 22C11, and APOE within and surrounding the β-amyloid plaque area, the outlines of the β-amyloid plaques were first determined from X-34 staining, and then the IBA1+, GFAP+, 22C11+, and APOE+ areas were analyzed within 0–40 µm, 0–40 µm, 0–14 µm, and 0–10 µm from the plaque outline, respectively (Figure S1A-D). The smallest area covering most of the IBA1, GFAP, 22C11 and APOE signals surrounding β-amyloid plaques was used to choose the width of the analyzed area. The image preprocessing and thresholding methods were kept constant between the samples within each staining. No signal was detected in the negative control samples. The ZEN 2012 software Blue edition (Carl Zeiss Microimaging GmbH) was used for visualization purposes.

Imaris (10.1.1) software was used for quantitative image analysis of the colocalization of 6E10 and IBA1. A surface for 6E10 and IBA1 was created using the surface tool. A Gaussian filter was applied via a smoothing function, and the surface detail value was set to 0.198 µm. The absolute intensity thresholding method was used for surface creation, with a set threshold of 3.8 for 6E10 and 12 for IBA1. Surfaces below 600 voxels in the IBA1 channel and 70.7 voxels in the 6E10 channel were filtered out from the analysis to exclude small particles. A mask was created from the 6E10 channel using the created IBA1 surface. The voxel intensity outside the IBA1 surface was set to 0 for this mask. A new surface was created from the mask using the same settings as those used to create the previous 6E10 surfaces. The new surface represents the overlapping signal of 6E10 IBA1. All image analysis parameters were kept the same for all the samples. The data are presented as the 6E10 volume inside the IBA1 surface as a percentage of the total 6E10 volume. All image analyses were performed by an investigator blinded to the genetic background of the mice.

### Acute CD11b+ microglia isolation

At 13 months of age, the mice were anesthetized with 60 mg/ml pentobarbital and briefly perfused with ice-cold DPBS to eliminate blood cells. The mice were then decapitated, and the brains were placed in ice-cold Hank's balanced salt solution without Ca^2+^ or Mg^2+^ (HBSS, #14,175,129, Gibco). The brains were mechanically dissociated into small pieces and further dissociated into single-cell suspensions via enzymatic digestion. Cell isolation was performed according to the instructions of the Adult Brain Dissociation Kit (#130-107-677, Miltenyi), and CD11b+ microglia were separated via CD11b MicroBeads, human and mouse (#130-093-634, Miltenyi) kits instructions. Magnetic separation was performed via a QuadroMACS Separator (#130-090-976, Miltenyi) with LS Columns (#130-042-401, Miltenyi). The cell pellet was washed twice with DPBS and immediately used for RNA extraction (below) or stored as a dry pellet at − 80 °C for proteomic analysis (below).

### Study subjects, MDMi preparation and analysis

Cognitively healthy PLCγ2-P522R monoallelic (GC, *APOE3/3,* age 68 ± 6.7, n = 7) carriers and matched controls (CC, *APOE3/3,* age 70 ± 5.5, n = 7) were recruited from 2022 to 2024 through the Auria Biobank on the basis of preexisting genome data returned to the biobank from FinnGen [21]. Blood samples for monocyte and DNA isolation were collected from the study subjects following written informed consent from each participant.

To extract peripheral blood mononuclear cells (PBMCs), 60–100 ml of peripheral venous blood was collected from the participants and processed within 24 h of sample collection. PBMCs were extracted by gradient centrifugation using Ficoll-Paque PLUS (#17–1440-02, Cytiva) in SepMate-50 tubes (#85450, Stemcell Technologies). Positive selection of monocytes from PBMCs was performed with human CD14 MicroBeads (#130-050-201, Miltenyi Biotec) and magnet-activated cell sorting.

Monocytes were plated at a density of 1 million cells per well in 12-well plates. Monocyte differentiation into MDMis was performed according to a previously published protocol [22] with modifications described in Martiskainen et al. [23]. After MDMi differentiation for 12 days in vitro, the conditioned medium was replaced with fresh MDMi culture medium or with culture medium containing myelin (25 µg/ml) or LPS (200 ng/ml, O26:B6, L5543, Sigma Aldrich), and the cells were cultured for 24 h prior to sample collection. For RNA extraction, the cells were washed three times with ice-cold PBS and stored in TRIzol reagent (#T9424 Sigma‒Aldrich). The RNA extraction protocol is described below.

### RNA extraction

The mouse temporo-occipital cortex was homogenized in TRIzol reagent (#T9424, Sigma‒Aldrich), after which chloroform was added (one-fifth of the volume of TRIzol reagent), and the tubes were shaken vigorously. The mixtures were incubated at room temperature for 5 min, and phase separation was performed by centrifugation at 12 000 × g for 20 min at 4 °C. The RNA was precipitated from the aqueous phase with 2-propanol by mixing and incubating at room temperature for 30 min. The samples were subsequently centrifuged at 12 000 × g for 25 min at 4 °C, after which the pellets were washed twice with 75% ethanol. The RNA pellets were air-dried and dissolved in RNAse-free H_2_O.

Isolated mouse CD11b+ microglia were resuspended in DPBS and immediately mixed with lysis buffer included in a High Pure RNA Isolation Kit (#11828665001, Roche). RNA was extracted following the kit instructions and sent for sequencing to Novogene Europe (below).

MDMi cells were washed once with 1 × DPBS, and the cells were collected with TRIzol reagent, where one-fifth of the TRIzol reagent volume of chloroform was added. The samples were mixed and incubated at room temperature for 2 min, followed by centrifugation at 12 000 × g for 15 min at 4 °C. The aqueous phase was collected, and 2-propanol was added at room temperature. The samples were vortexed and incubated at room temperature for 10 min. The RNA was washed twice with 75% ethanol and centrifuged at 13 000 × g for 5 min at 4 °C. The supernatant was removed, and the remaining ethanol was allowed to evaporate at room temperature for approximately 10 min. The RNA pellet was resuspended in RNase-free H_2_O and sent for sequencing to Novogene Europe (below).

### RNA sequencing and data analysis

Library preparation and RNA sequencing were conducted by Novogene (Cambridge, UK) and executed with an Illumina high-throughput sequencing platform. In brief, mRNA enrichment was performed with oligo(dT) bead pulldown, from which the pulldown material was subjected to fragmentation, followed by reverse transcription, second strand synthesis, A-tailing, and sequencing adaptor ligation. The final amplified and size-selected library comprised 250–300 bp of inserted cDNA and 150 bp paired-end sequences. Sequencing yielded reads for 20.2–31.34, 29.4–49.9, and 19.3–31.8 million library fragments per CD11b+ mouse microglia, mouse cortex, and human MDMi cells, respectively.

The 150-nucleotide pair-end RNA-seq reads were quality controlled using FastQC (version 0.11.7) (https://www.bioinformatics.babraham.ac.uk/projects/fastqc/). The reads were then trimmed with Trimmomatic (version 0.39) [24] to remove Illumina sequencing adapters and poor-quality read ends via the following essential settings: ILLUMINACLIP:2:30:10:2:true, SLIDINGWINDOW:4:10, LEADING:3, TRAILING:3, and MINLEN:50. The trimmed reads were aligned to the Gencode mouse transcriptome version M25 (for genome version GRCm38) or the human transcriptome version 38 (for genome version GRCh38) using STAR (version 2.7.9a) [25] with essential nondefault settings: –seedSearchStartLmax 12, –alignSJoverhangMin 15, –outFilterMultimapNmax 100, –outFilterMismatchNmax 33, –outFilterMatchNminOverLread 0, –outFilterScoreMinOverLread 0.3, and –outFilterType BySJout. The unstranded, uniquely mapped, genewise counts for primary alignments produced by STAR were collected in R (version 4.2.2 or 4.3.2) using Rsubread::featureCounts (version 2.12.0, 2.12.3 or 2.16.1)[26], ranging from 16.2 to 19.7, 25.1 to 43.1, or 14.5 to 25.1 million per CD11b+ mouse microglia, mouse cortex, and human MDMi cells, respectively. Differentially expressed genes (DEGs) between experimental groups were identified in R (version 4.3.2) using DESeq2 (version 1.38.3) [27] by employing the Wald statistic and lfcShrink for FC shrinkage (type = “apeglm”) [28]. For the human MDMi samples, comparisons between PLCγ2-P522R variant carriers (GC) and matched controls (CC) were performed while adjusting for the sample delivery batch. Pathway enrichment analysis was performed on the gene lists ranked by the pairwise DEG test log2FCs in R (version 4.2.3) using clusterProfiler::GSEA (version 4.6.2) [29] with Molecular Signatures Database gene sets (MSigDB, version 2022.1 for mouse CD11b+ microglia, and version 2023.1 for the mouse cortex and human MDMi) [30].

### Proteomic analysis

Sample preparation for proteomic analyses of acutely isolated CD11b+ microglia from PLCγ2-P522R mice was carried out as described previously by Martiskainen et al. [23]. A total of 350 ng of peptide was separated on an in-house packed C18 analytical column (15 cm × 75 µm ID, ReproSil-Pur 120 C18-AQ, 1.9 µm, Dr. Maisch GmbH) using a 120 min long binary gradient of water and acetonitrile (B) containing 0.1% formic acid at a flow rate of 300 nL/min and a column temperature of 50 °C for CD11b+ microglia. A Data Independent Acquisition Parallel Accumulation–Serial Fragmentation (DIA-PASEF) method with a cycle time of 1.9 s was used for spectrum acquisition. Briefly, ion accumulation and separation via trapped ion mobility spectrometry (TIMS) was set to a ramp time of 100 ms. One scan cycle included one TIMS full MS scan followed by 17 DIA PASEF scans with two 26 m/z windows covering the m/z range from 350–1200 m/z.

The raw data were analyzed using the software DIA-NN version 1.8 [31] for protein label-free quantification (LFQ). One protein per gene canonical fasta database of *Mus musculus* (download date: January 12th 2023, 21957 entries) from UniProt and a fasta database with 125 potential human contaminations from MaxQuant [32]. Trypsin was defined as a protease. DIA-NN was used to generate a spectral library on the basis of the individual datasets and to perform label-free quantification. Two missed cleavages were allowed, and peptide charge states were set to 2–4. Carbamidomethylation of cysteine was defined as a static modification. Acetylation of the protein N-term as well as oxidation of methionine were set as variable modifications. The false discovery rate for both peptides and proteins was adjusted to less than 1%. Data normalization was disabled.

The identification of differentially expressed proteins between genotypes, nonnormalized intensities as the starting point, and subsequent pathway enrichment were performed for the corresponding RNA-seq data, with the exception of MSigDB version 2023.1 [30]. To facilitate direct comparisons between proteomic and RNA-seq data, the respective gene symbols were used instead of the UniProt protein IDs.

### Mouse primary microglia cultures

Homozygous PLCγ2-P522R KI and WT mouse pups (P0-P3) were decapitated, and the brains were removed and placed in 6-cm petri dishes in ice-cold HBSS (#24020091, Thermo Fisher). Meninges were removed under a microscope, and the brains were washed three times with ice-cold HBSS. For tissue lysis, 0.25% trypsin (#15090046, Gibco) was added, and the brains were incubated for 10 min at 37 °C in a cell culture incubator. The reaction was stopped by adding culture medium containing DMEM with 4.5 g/L glucose without L-glutamine (#ECB7501L, BioNordica), 10% (v/v) heat-inactivated fetal bovine serum (FBS, #10500–064, Gibco), 2 mM L-glutamine (#BE17-605E, Lonza Bioscience), and 1% (v/v) penicillin‒streptomycin (#DE17-602E, Lonza Bioscience). Next, 400 µl of 5 mg/ml deoxyribonuclease I (#D4527-40KU, Sigma) was added, and the cells were separated by pipetting carefully. The cells were then centrifuged for 10 min at 800 × g at room temperature and resuspended in culture medium. The cells were cultured in T-75 cell culture flasks coated with 0.02% poly-L-lysine hydrobromide (PLL, #P6407, Sigma) until the microglia were shaken one week later and then every 3‒4 days.

### Myelin preparation

Myelin was isolated from the brains of adult male C57BL/6J mice by a decreasing sucrose gradient according to a protocol described previously [33, 34]. Briefly, the brain tissue was homogenized in a solution containing 10 mM HEPES, 5 mM EDTA, 0.32 M sucrose and protease inhibitor. The homogenate was layered on top of 0.85 M sucrose solution prepared in HEPES-EDTA buffer and centrifuged at 75 000 × g for 30 min with low acceleration and deceleration using a Beckman Ultracentrifuge with a Ti 50.2 rotor. The white myelin layer was collected from the interface, suspended in sterile water, and centrifuged at 75 000 × g for 15 min. The pellet was purified twice by hypo-osmotic shock using ice-cold water and centrifuged at 12 000 × g for 10 min. After that, the sucrose gradient and hypo-osmotic shocks were repeated to purify the myelin fraction. Finally, the myelin pellet was resuspended in HEPES-EDTA buffer and stored at − 80 °C. The concentration of myelin was determined using a BCA protein assay kit (#23225, Pierce).

The extracted myelin was labeled with a pHrodo^™^ iFL Red Microscale Protein Labeling Kit (Thermo Fisher Scientific, P36014) by adding 25 µl of dye per 1 mg of myelin and incubating for 60 min at room temperature. The myelin was then pelleted by centrifuging for 10 min at 15 000 × g and washed twice with 1 × DPBS to remove the excess dye. pHrodo-labeled myelin was resuspended in 1 × DPBS at a concentration of 1 mg/ml and stored as aliquots at − 80 °C.

### Lipid droplet analysis

To analyze lipid accumulation in mouse primary microglia, 50 000 cells were plated onto a 48-well plate on top of glass cover slips precoated with 0.02% PLL in microglial culture medium (above) with reduced (5%) FBS. The next day, the cells were treated with 5 µg/ml LPS (#L5543; Sigma‒Aldrich) for 24 h or with 30 µg/ml myelin for 48 h. Control wells were left untreated. The cells were then washed three times with warm DPBS and stained with 2 µM BODIPY 493/503 (#D3922, Invitrogen) for 15 min in a cell culture incubator. The cells were rinsed several times with DPBS, fixed with 4% PFA for 20 min at room temperature and mounted on microscope glasses with a Vectashield Hard Set containing phalloidin (#H-1699, Vector Laboratories).

Images of primary microglia stained with BODIPY 493/503 to visualize neutral lipid droplets (LDs) and phalloidin to visualize the actin cytoskeleton (cell border) were acquired with a Zeiss Axio Observer inverted microscope with a Zeiss LSM 700 confocal module using a 20 × (NA 0.5) objective. Five to ten 1024 × 1024-pixel fields per cover slip were obtained. All the laser and detector settings were kept constant for all the samples. Imaging was performed by an investigator blinded to the genetic background and treatment of the cells.

LDs were analyzed via ImageJ (version 1.53c). LDs were segmented via the Intermodes automatic threshold method. Outliers with a radius of 3 and threshold of 50 were removed from the mask, after which particles larger than 100 pixels were included in the particle analysis. The number of LD-positive cells was manually counted. Analysis was performed by an investigator blinded to the genetic background and treatment of the cells. The image preprocessing and thresholding methods were kept constant throughout the analysis. The ZEN 2012 software Blue edition (Carl Zeiss Microimaging GmbH) was used for visualization purposes. The percentage of LD-positive cells among all the cells and the average LD size (µm^2^) per imaged field are shown. The numbers of analyzed images per group were as followed: WT untreated = 11, LPS = 22, myelin = 42 and KI PLCγ2-P522R untreated = 6, LPS = 12, myelin = 35.

### iMGL preparation

The original iPSC line used in this study, carrying *APOE4/4* and control *PLCG2* genotype CC/*APOE4/4* (UKBi011-A, https://hpscreg.eu/cell-line/UKBi011-A), was described previously [35]. The iPSC line CC/*APOE3/3* (UKBi011-A3, https://hpscreg.eu/cell-line/UKBi011-A-3) was generated from the original line using CRISPR/Cas9 as previously described [36]. For the generation of homozygous PLCγ2-P522R lines on the *APOE3/3* (GG/*APOE3/3*) and *APOE4/4* (GG/*APOE4/4*) backgrounds, the gRNA CCCCAAAATGTAGTTCTGTA was used. The sequence of the ssODN used as an HDR donor was GTGAGACAGAAGGACCTGTCTAGTGATGCTGGGGTTTGGTCCAAGGCTTTCAGAAACCCCTCCTCTCTTTGCGGCCCAGGATATACGCCCGACGGAGCTACATTTTGGGGAGAAATGGTTCCACAAG.

Human iPSC lines were cultured in Essential 8 (E8) medium (#A1517001, Gibco) on 3.5 cm dishes coated with Matrigel (#356231, Corning) (#83.3900.500, Sarstedt) at 37 °C in 5% CO_2_ and split with 0.5 mM EDTA every 4–5 days. For iMGL differentiation from iPSCs, a protocol described previously was used [37–39]. Briefly, iPSC colonies were detached using ReLeSR reagent (#5872, STEMCELL Technologies) and plated at a density of 3‒6 colonies per cm^2^ on Matrigel-coated 6-well plates (#356231, Corning) in E8 medium supplemented with 5 µM ROCK inhibitor (#Y-27632, Merck). On the following day, differentiation into hematopoietic stem cells was initiated via a commercial STEMdiff Hematopoietic kit (#05310, STEMCELL Technologies). After 11‒13 days of differentiation, the floating hematopoietic progenitors were collected and plated at a density of 7000‒8000 cells per cm^2^ on new Matrigel-coated 6-well plates in microglial differentiation medium consisting of DMEM/F12 (#21331020, Gibco), 2 × insulin-transferrin-selenite (#41400045, Invitrogen), 2 × B27 (#17504044, Invitrogen), 0.5 × N2, 1 × GlutaMAX (#35050‒038, Invitrogen), 1 × nonessential amino acids (#11140050, Invitrogen), 400 μM monothioglycerol (#M1753, Sigma), 5 μg/mL human insulin (#19278, Sigma), 100 ng/mL human IL-34 (#200‒34, PeproTech), 50 ng/mL human TGF-β1 (#100‒21, PeproTech), and 25 ng/mL human M-CSF (#300‒25, PeproTech). The cells were grown for 27 days, and fresh medium was added every other day. During the last 4 days of culture, microglial maturation was promoted by adding 100 ng/mL human CD200 (#77002, Biolegend) and 100 ng/mL human CX3CL1 (#300–31, PeproTech) to the cells.

### Seahorse assay for mitochondrial and glycolytic energy metabolism

To perform the cell Mito Stress test in the iMGLs, 40 000 cells per well were seeded in 200 μl of maturation medium one week before the experiment. Every other day, half of the medium was replaced with fresh medium. On the day of the experiment, Seahorse XF assay medium was prepared by adding 2 mM Glutamax (#35050–038, Invitrogen) to Seahorse DMEM. First, the cells were rinsed with 180 μl of Seahorse XF medium following the addition of Seahorse XF medium to the cells to a final volume of 180 μl. The cells were incubated in a non-CO_2_ incubator for 1 h at 37 °C before being run on an XFe96 Analyzer. At the beginning of the assay, the XFe96 Analyzer added 10 mM glucose and 1 mM sodium pyruvate (#11360,039, Gibco) to the cells. Next, modulators of the electron transport chain were injected at a 1 μM concentration into the cells in the following order: oligomycin (#11342, Cayman Chemical), carbonyl cyanide-4 (trifluoromethoxy) phenylhydrazone (FCCP) (#15218, Cayman Chemical) and a mixture of rotenone (#13995, Cayman Chemical) and antimycin A (#A8674, Sigma‒Aldrich). During the assay, the oxygen consumption rate (OCR) was directly measured by the XFe96 Analyzer. The results were normalized to the cell confluence determined by an IncuCyte S3 (Sartorius) before the beginning of the assay. The results are shown from three independent Seahorse XF96 cell culture plates, each with 3‒4 technical replicates per line.

### Statistical analyses

GraphPad Prism 10.1.2 software was used for all the statistical analyses and for the visualization of the data. The Shapiro‒Wilk test was used to test whether the data had a normal distribution. Accordingly, the statistical significance was tested via an unpaired samples t test or the Mann‒Whitney test. In the case of multiple dependent variables, two-way ANOVA together with Šídák's or Tukey’s multiple comparisons test was used. Correlation analyses were performed via Pearson correlation coefficients. P values less than 0.05 were considered statistically significant. All the data are presented as the means ± SEMs.

## Results

### Microglial activation is increased in 13-month-old APP/PS1 mice carrying the PLCγ2-P522R variant

**Fig. 1:**
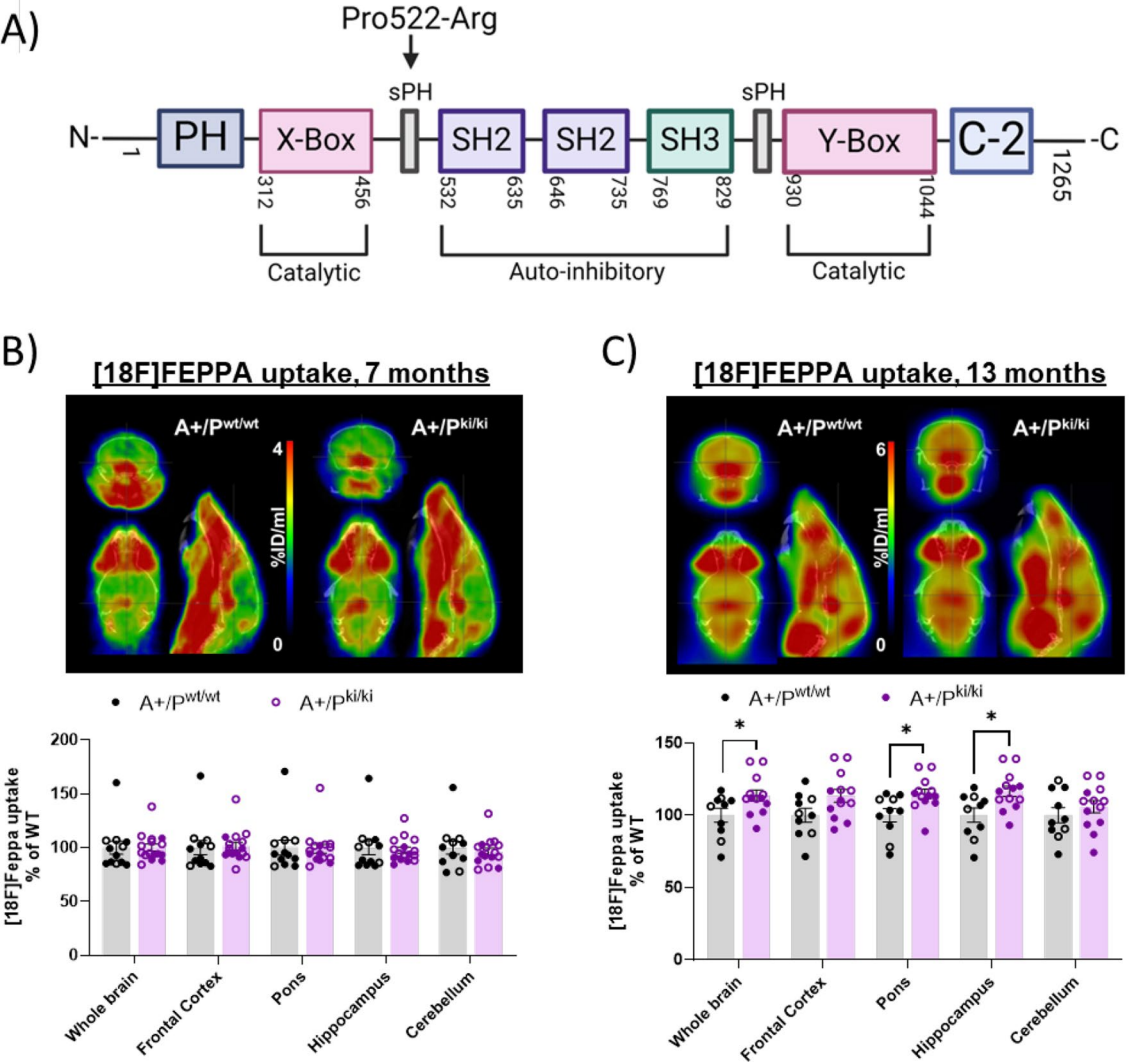
PLCγ2-P522R variant increases microglia activation in the brain of 13-month-old female and male APP/PS1 mice. **A** Illustration of the PLCγ2 protein domain and the location of the PLCγ2-P522R variant in the protein domain. Created in https://BioRender.com. **B**-**C** Example positron emission (PET) images of the uptake (percent of the injected dose per ml of tissue) of [18F]FEPPA for both APP/PS1 (A+/P^wt/wt^) and APP/PS1xPLCγ2-P522R (A+/P^ki/ki^) mice at (B) 7 months and (**C**) 13 months of age with an anatomical CT reference image in the background. [18F]FEPPA uptake remains unaltered between the genotypes at the 7-month timepoint. A+/P^wt/wt^ n = 12 and A+/P^ki/ki^ n = 17. Compared with that in A+/P^wt/wt^ mice, [18F]FEPPA uptake in the whole brain (*p = 0.032), pons (*p = 0.013), and hippocampus (*p = 0.012) was significantly greater in 13-month-old A+/P^ki/ki^ mice. A+/P^wt/wt^ n = 10 and A+/P^ki/ki^ n = 13. The colored circles indicate data obtained from female mice, and the hollow circles indicate data obtained from male mice. The data were normalized to those of A+/P^wt/wt^ mice of each sex

*PLCG2* is highly expressed in microglia [3]. We have previously shown that the protective PLCγ2-P522R (rs72824905, p.P522R, Fig. 1A) variant increases microglia activation in WT mice with no β-amyloid pathology [7]. We now analyzed whether the PLCγ2-P522R variant promotes microglial activation in APP/PS1 (hereafter referred to as A+/P^wt/wt^) mice using PET imaging with the TSPO ligand [18F]FEPPA, which is highly expressed in activated microglia [20]. To increase statistical power, data from female and male mice were pooled for analysis. Given the baseline difference between the sexes, the data were normalized to the average A+/P^wt/wt^ value within each sex. There was no difference in [18F]FEPPA ligand uptake between the genotypes in 7-month-old mice (Fig. 1B). However, in 13-month-old animals, increased [18F]FEPPA ligand uptake was observed in the whole brain (p = 0.032) of APP/PS1 x PLCγ2-P522R KI (hereafter referred to as A+/P^ki/ki^) mice compared with A+/P^wt/wt^ mice. When the brain regions were examined separately, [18F]FEPPA ligand uptake was significantly increased in the pons (p = 0.013) and hippocampus (p = 0.012) (Fig. 1C). These findings suggest that microglial activation in the brain tissue of APP/PS1 mice carrying the PLCγ2-P522R variant moderately increases at 13 months of age.

### β-amyloid plaque pathology is mitigated in female APP/PS1 mice carrying the PLCγ2-P522R variant

**Fig. 2:**
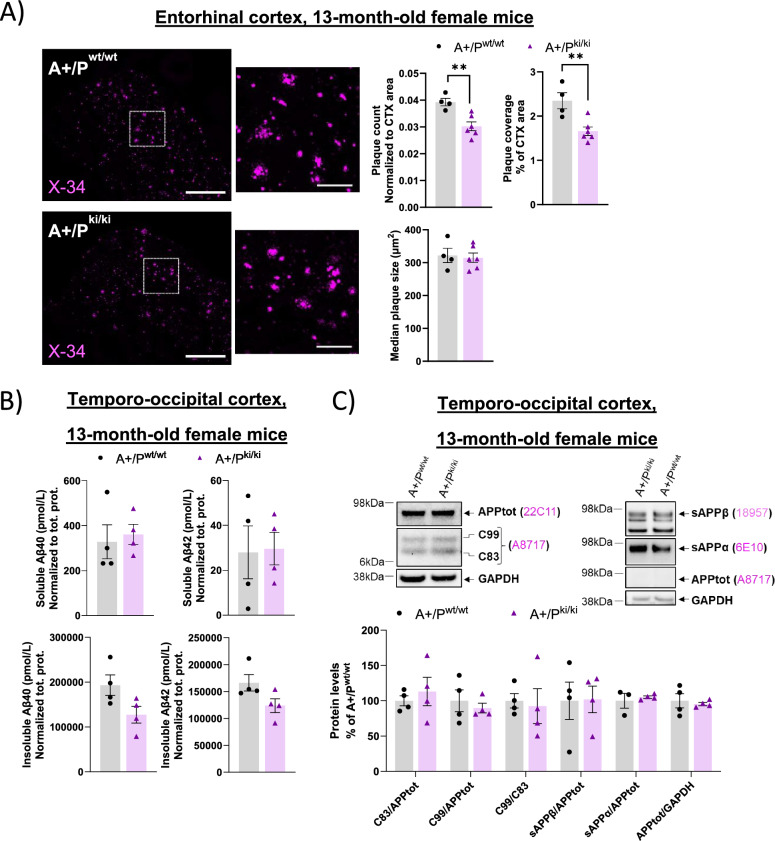
β-amyloid pathology is mitigated in female APP/PS1 mice carrying the PLCγ2-P522R variant. **A** X-34-positive β-amyloid plaque count (*p = 0.005) and coverage (**p = 0.006, total plaque area, % of whole analyzed area) were lower in the entorhinal cortex of APP/PS1xPLCγ2-P522R (A+/P^ki/ki^) mice than in that of APP/PS1 (A+/P^wt/wt^) mice. The size (µm^2^) of the individual plaques did not differ between the genotypes. A+/P^wt/wt^ n = 4 and A+/P^ki/ki^ n = 6. **B** Insoluble but not soluble Aβ40 and −42 levels are slightly but not significantly lower in the temporo-occipital cortex of A+/P^ki/ki^ mice than in that of A+/P^wt/wt^ mice. Aβ40 and Aβ42 levels were normalized to the total protein concentration in the same sample. A+/P^wt/wt^ n = 4 and A+/P^ki/ki^ = 4. **C** Representative Western blots and corresponding quantification showing no differences in the levels of full-length APP (APPtot, normalized to GAPDH), APP C-terminal fragments (C99 and C83, normalized to APPtot), or soluble APPα and APPβ (sAPPα, sAPPβ, normalized to APPtot) species in the temporo‒occipital cortex of A+/P^ki/ki^ and A+/P^wt/wt^ mice. A+/P^wt/wt^ n = 4 and A+/P^ki/ki^ n = 4. The scale bars in the representative immunofluorescence images are 657 µm for the whole area and 164 µm for the zoomed view. Unpaired samples t test. All the data are presented as the means ± SEMs. Each data point represents an individual mouse

Given that microglial activation was observed in the brain tissue of 13-month-old mice but not in those of younger animals, the following characterizations of brain pathology were carried out in female mice at this time point. In general, the APP/PS1 female mice used in this study presented more pronounced β-amyloid-related pathology and greater learning and memory impairments than their male counterparts did [40]. Therefore, we concentrated our subsequent assessments on female A+/P^ki/ki^ and A+/P^wt/wt^ mice. The PLCγ2-P522R variant was previously shown to reduce β-amyloid pathology in 5XFAD mice [2, 17]. To study whether the PLCγ2-P522R variant affects brain β-amyloid pathology in APP/PS1 mice, we stained β-amyloid plaques using the fluorescent amyloid stain X-34 [41]. Image analysis revealed that β-amyloid plaque number (normalized to the size of the analyzed area, p = 0.005) and β-amyloid plaque coverage (total plaque area % of the size of the analyzed area, p = 0.006) were both significantly lower in the entorhinal cortex of the A+/P^ki/ki^ mice than in that of the A+/P^wt/wt^ mice (Fig. 2A). However, a similar reduction was not detected in the hippocampus of the same mice (Supplementary Figures, Figure S2A). The average size of individual X-34+ β-amyloid plaques did not differ between the genotypes in either the entorhinal cortex (Fig. 2A) or the hippocampus (Supplementary Figures, Figure S2A).

The soluble and insoluble levels of Aβ40 and Aβ42 in the ELISA did not significantly differ in the temporo-occipital cortex between A+/P^ki/ki^ and A+/P^wt/wt^ mice (Fig. 2B). Similarly, there were no significant changes in the levels of soluble and insoluble Aβ40 and Aβ42 in the hippocampus of A+/P^ki/ki^ mice compared with those in the hippocampus of A+/P^wt/wt^ mice (Supplementary Figures, Figure S2B). Importantly, there were no differences in the levels of full-length APP (APPtot), APP C-terminal fragments (C83 and C99, Fig. 2C and Supplementary Figures, S2C, left panel), or soluble APPα and -β (sAPPα and -β, Fig. 2C and Supplementary Figures, S2C, right panel) in the temporo-occipital cortex or hippocampus according to Western blot analyses of these mice, suggesting that the histochemically determined decrease in the β-amyloid pathology of A+/P^ki/ki^ mice was not due to alterations in APP stability or processing.

### The area of microglia around β-amyloid plaques is increased in female APP/PS1 mice carrying the PLCγ2-P522R variant

**Fig. 3:**
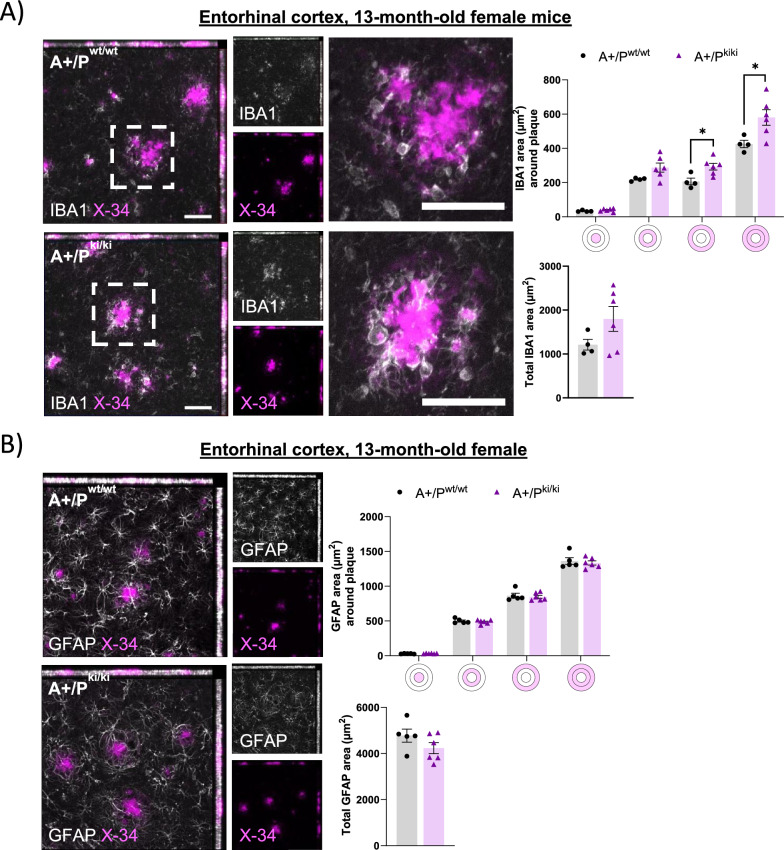
PLCγ2-P522R variant increases the area of microglia around β-amyloid plaques in female APP/PS1 mice. **A** The area (µm^2^) of IBA1-positive microglia was greater at distances of 20–40 µm (*p = 0.017) and 0–40 µm (*p = 0.03) from the plaque outline in the entorhinal cortex of APP/PS1xPLCγ2‒P522R (A+/P^ki/ki^) mice than in that of APP/PS1 (A+/P^wt/wt^) mice. Simultaneously, a trend toward an increase in the total IBA1 area is observed. A+/P^wt/wt^ n = 4 and A+/P^ki/ki^ n = 6. **B** GFAP-positive astrocyte area (µm^2^) around plaques (within 0–20 µm and 20–40 µm from the plaque outline) and total GFAP area remain unaltered between the genotypes. A+/P^wt/wt^ n = 5, A+/P^ki/ki^ n = 6. The colored disks on the x-axis of A and B indicate the analyzed area overlapping (middle circle) and surrounding β-amyloid plaques. The scale bar in the representative immunofluorescence images is 50 μm. Unpaired samples t test. All the data are presented as the means ± SEMs. Each data point represents an individual mouse

To assess whether PLCγ2-P522R affects the microglial response to β-amyloid deposition, we quantified the IBA1+ area within and surrounding X-34-positive β-amyloid plaques in the APP/PS1 mouse brain (Supplementary Figures, Figure S1A). The IBA1+ area was increased within the 0–40 µm radius from the β-amyloid plaque outline in the entorhinal cortex (p = 0.03, Fig. 3A) and hippocampus (p = 0.037, Supplementary Figures, Figure S3A) of A+/P^ki/ki^ mice compared with A+/P^wt/wt^ mice. No difference was found in the IBA1+ signal overlapping the β-amyloid plaque area itself, either in the entorhinal cortex or hippocampus. The total IBA1+ area in the entorhinal cortex was marginally increased, whereas in the hippocampus, the total IBA1+ area was decreased (p = 0.043). Together, these data suggest that the PLCγ2-P522R variant increases the area of microglia around β-amyloid plaques and consequently, may resist β-amyloid deposition more efficiently.

Since activated microglia secrete chemokines and cytokines that promote astrocyte activation [42], we stained astrocytes with a glial fibrillary acidic protein (GFAP) antibody to study potential changes in astrocytes in the entorhinal cortex and hippocampus of APP/PS1 mice [42]. We found no differences in the total GFAP+ area or in the GFAP+ area overlapping or surrounding (within the 0–40 µm radius) X-34-positive β-amyloid plaques (Supplementary Figures, Figure S1B) in either the entorhinal cortex (Fig. 3B) or the hippocampus (Supplementary Figures, Figure S3B) of A+/P^ki/ki^ mice compared with A+/P^wt/wt^ mice, suggesting that PLCγ2-P522R variant-driven microglial activation does not promote astrocyte activation in the brain tissue of 13-month-old APP/PS1 mice.

### The number of dystrophic neurites around β-amyloid plaques is decreased in female APP/PS1 mice carrying the PLCγ2-P522R variant

**Fig. 4:**
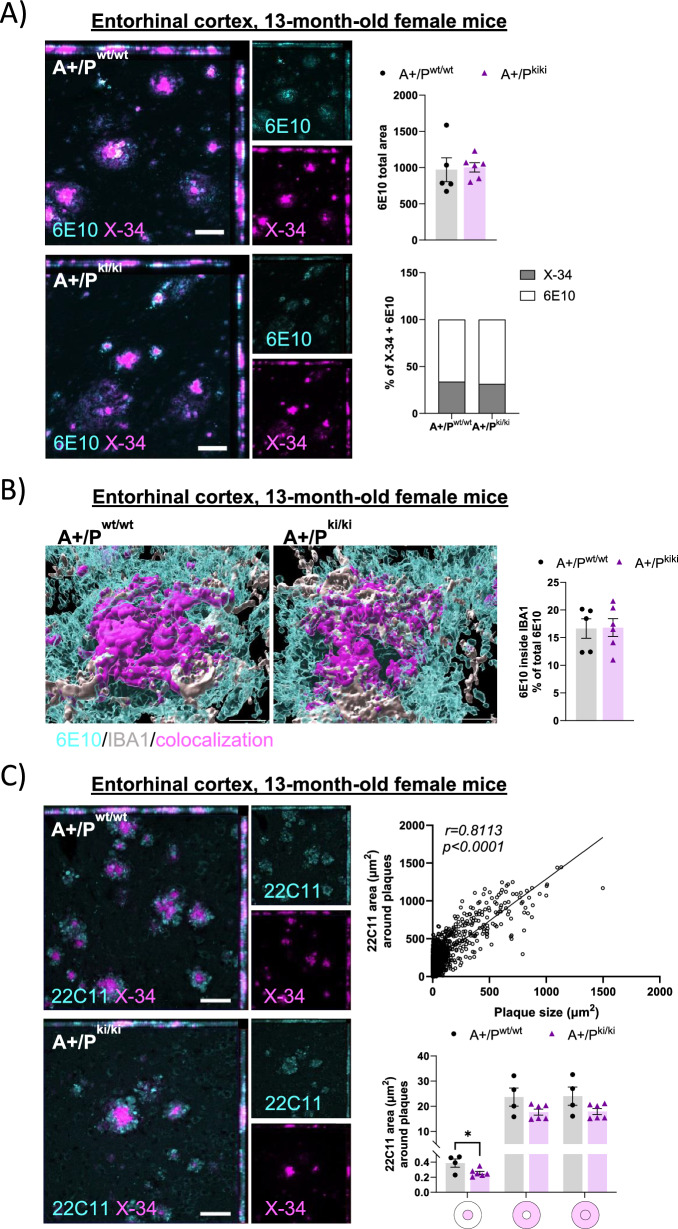
PLCγ2-P522R variant decreases β-amyloid plaque-associated dystrophic neurites in female APP/PS1 mice. **A** Total area (µm^2^) of diffuse β-amyloid (6E10) and composition β-amyloid plaques, as indicated by the percentage of compact (X-34) and diffuse (6E10) β-amyloid plaques, remaining unaltered in the entorhinal cortex of the APP/PS1xPLCγ2-P522R (A+/P^ki/ki^) mice compared with the APP/PS1 (A+/P^wt/wt^) mice. A+/P^wt/wt^ n = 5 and A+/P^ki/ki^ n = 6. **B**) A 3D reconstruction showing the 6E10 and IBA1 (microglia) signals and their colocalization. Quantification of the 6E10 signal within the IBA1-positive area as a percentage of all the 6E10 signal in the entorhinal cortices of the A+/P^ki/ki^ mice compared with that in the A+/P^wt/wt^ mice. **C** The area (µm^2^) of 22C11-labeled dystrophic neurites around β-amyloid plaques strongly correlates with plaque size (r = 0.8113, ****p < 0.0001). The 22C11-positive area was decreased within the β-amyloid plaque area (p = 0.033), and a similar decreasing trend was observed within the 0–14 µm radius from the β-amyloid plaque outline in the entorhinal cortices of the A+/P^ki/ki^ mice compared with the A+/P^wt/wt^ mice when the plaque size was normalized. A+/P^wt/wt^ n = 4 and A+/P^ki/ki^ n = 6. Colored disks on the x-axis indicate the analyzed area overlapping (middle circle) and surrounding β-amyloid plaques. Scale bars in the representative immunofluorescence images are 50 μm. Pearson correlation and unpaired samples t tests were used. All the data are presented as the means ± SEMs. Each data point represents an individual mouse

Microglia clustered around β-amyloid plaques have been suggested to pack diffuse β-amyloid into a more compacted and less toxic form [43]. Hence, to investigate β-amyloid plaque morphology, we used X-34 to detect compact plaques and the anti-β-amyloid antibody 6E10 to detect diffuse plaques. Despite the evident improvement in the microglial response toward β-amyloid plaques, we found no differences in the total 6E10+ area or in the percentage of X-34+ or 6E10+ area of total β-amyloid pathology (X-34 + 6E10) either in the entorhinal cortex (Fig. 4A) or in the hippocampus (Supplementary Figures, Figure S4A) between the mice with different genotypes. Furthermore, no differences in the colocalization of 6E10 within the IBA1+ area were detected between the mice with different genotypes (Fig. 4B and Supplementary Figures, S4B), suggesting that microglial uptake of diffuse β-amyloid nor the ratio of diffuse and compact β-amyloid in the brain tissue are affected in 13-month-old APP/PS1 mice carrying the PLCγ2-P522R variant.

To assess the formation of dystrophic neurites around β-amyloid plaques in the brain tissue of APP/PS1 mice carrying the PLCγ2-P522R variant, brain sections were stained with the N-terminal anti-APP antibody 22C11. It is well documented that IHC staining with 22C11 shows the locations of swollen neuronal processes linked to abnormal protein accumulation, including abnormal N-terminal APP and phosphorylated Tau (p-Tau) accumulation, in different *in vivo* models of AD [44–47]. Since the 22C11 signal was strongly dependent on the Aβ plaque size (Pearson correlation coefficient (r) = 0.81, p < 0.0001) (Fig. 4C), we normalized the 22C11+ area around each plaque to the size of the analyzed plaque. The 22C11+ area overlapping the plaque area in the entorhinal cortex was significantly smaller (p = 0.033) in A+/P^ki/ki^ mice than in A+/P^wt/wt^ mice, and a similar but nonsignificant trend was observed within the 0–14 µm radius from the plaque outline (Supplementary Figures, Figure S1C and 4C). In the hippocampus, the 22C11+ area was significantly decreased within the 0–14 µm radius from the plaque outline (p = 0.010) (Supplementary Figures, Figure S4C). In addition to the N-terminal APP, dystrophic neurites contain various other proteins, including p-Tau [44–47]. Thus, the levels of p-Tau relative to total Tau (ratio of p-Tau/total Tau) were assessed to gain insight into the extent of p-Tau pathology and its association with dystrophic neurites in protein lysates from the temporo-occipital cortex and hippocampus of 13-month-old female A+/P^ki/ki^ and A+/P^wt/wt^ mice **(**Supplementary Figures, Figure S5A and S5B). Consequently, Western blot analysis did not reveal significant differences in the ratio of p-Tau/total Tau between A+/P^ki/ki^ and A+/P^wt/wt^ mice in the tissue lysates studied. Taken together, these findings suggest that the PLCγ2-P522R variant decreases the formation of dystrophic neurites around β-amyloid plaques, as shown by N-terminal APP staining, whereas a similar decrease in the p-Tau/total Tau ratio in the tissue lysates was not detected.

### Neuroinflammation is alleviated in the brain tissue of female APP/PS1 mice carrying the PLCγ2-P522R variant

**Fig. 5:**
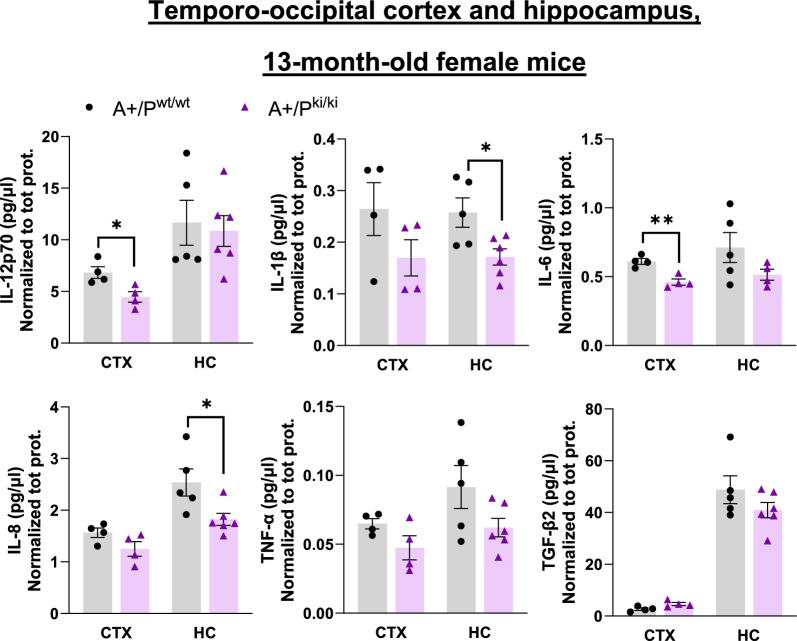
PLCγ2-P522R variant alleviates neuroinflammation in the brain tissue of female APP/PS1 mice. **A** IL-12p70 (p = 0.049) and IL-6 (p = 0.003) levels are significantly lower in the temporo-occipital cortex (CTX), whereas IL-1β (p = 0.021) and IL-8 (p = 0.026) levels are significantly lower in the hippocampus (HC) of APP/PS1xPLCγ2-P522R (A+/P^ki/ki^) mice than in those of APP/PS1 (A+/P^wt/wt^) mice. In addition, the IL-1β, IL-8, and TNF-α levels tended to decrease in the CTX group, whereas the IL-6, TNF-α, and TGF-β2 levels tended to decrease in the HCs of the A+/P^ki/ki^ mice compared with those of the A+/P^wt/wt^ mice. CTX A+/P^wt/wt^ n = 4 and A+/P^ki/ki^ n = 4; HC A+/P^wt/wt^ n = 5 and HC A+/P^ki/ki^ n = 6. Unpaired samples t test. All the data are presented as the means ± SEMs. Each data point represents an individual mouse

The well-established ability of microglia to compact β-amyloid deposits and protect surrounding neurons is considered beneficial. However, microglial activation also promotes neuroinflammation, which is harmful to neuronal health over time. Therefore, we next analyzed the levels of several inflammatory markers in the temporo-occipital cortex and hippocampal lysates of 13-month-old mice via an MSD mouse inflammatory panel. In general, A+/P^ki/ki^ mice presented lower levels of several inflammatory markers in both the temporo-occipital cortex and hippocampus than did A+/P^wt/wt^ mice (Fig. 5). The levels of IL-12-p70 (p70 refers to an active heterodimer, p = 0.049) and IL-6 (p = 0.003) in A+/P^ki/ki^ mice were significantly lower in the cortex, while those of IL-1β (p = 0.021) and IL-8 (p = 0.026) were lower in the hippocampus. These findings suggest that despite increased microglial activation, the PLCγ2-P522R variant does not increase chronic β-amyloid-associated neuroinflammation in the APP/PS1 mouse brain upon aging.

### PLCγ2-P522R variant does not increase β-amyloid plaque-associated APOE or promote DAM signature in the female APP/PS1 mice

**Fig. 6:**
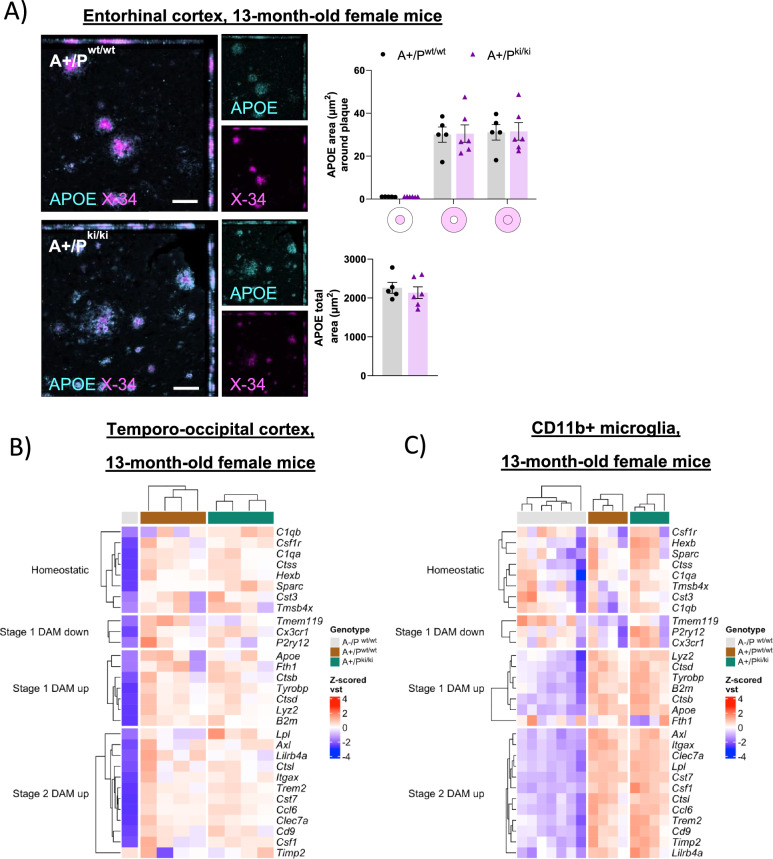
PLCγ2-P522R variant does not increase the plaque-associated APOE or DAM signature in female APP/PS1 mice. **A** Analysis of total APOE and APOE areas (µm^2^) within and surrounding (within 0–10- and 10–20-µm distances from the amyloid plaque outline) β-amyloid plaques revealed no differences between the APP/PS1xPLCγ2-P522R (A+/P^ki/ki^) and APP/PS1 (A+/P^wt/wt^) mice. A+/P^wt/wt^ n = 5, A+/P^ki/ki^ n = 6. Colored disks on the x-axis indicate the analyzed area overlapping (middle circle) and surrounding β-amyloid plaques. The scale bar in the representative immunofluorescence images is 50 μm. Unpaired samples t test. All the data are presented as the means ± SEMs. Each data point represents an individual mouse. **B** Heatmap of z scored vst-normalized expression of homeostatic as well as stage 1 and stage 2 disease-associated microglia (DAM) signature genes in the bulk temporo-occipital cortex and **C**) CD11b+ microglia isolated from the whole brain tissue of 13-month-old wild-type (A-/P^wt/wt^), A+/P^wt/wt^ and A+/P^ki/ki^ female mice. Cortex A-/P^wt/wt^ n = 1, A+/P^wt/wt^ n = 4, and A+/P^ki/ki^ n = 4; CD11b+ A-/P^wt/wt^ n = 7, A+/P^wt/wt^ n = 4, and A+/P.^ki/ki^ n = 4

β-Amyloid plaque-associated APOE has been suggested to increase plaque compaction and support microglial responses toward β-amyloid deposition [48, 49]. To address whether APOE plays a role in the PLCγ2-P522R variant-mediated microglial response to Aβ, we analyzed the APOE+ area within and surrounding plaques. We found no differences in the APOE+ area overlapping or within a 10 µm radius from the plaque outline (Supplementary Figures, Figure S1D) between A+/P^ki/ki^ and A+/P^wt/wt^ mice in the entorhinal cortex (Fig. 6A) or in the hippocampus (Supplementary Figures, Figure S6A). In line with these findings, soluble and insoluble APOE levels in the temporo-occipital cortex and hippocampus did not differ between the mice with different genotypes (Supplementary Figures, Figure S6B).

The upregulation of *Apoe* is part of the disease-associated microglia (DAM) signature [5]. DAM activation is regulated by *Trem2* expression and is needed for restricting β-amyloid pathology in AD [5]. Hence, to study whether the protective variant exacerbates the RNA signature related to DAM activation in the brain tissue of 13-month-old APP/PS1 mice, RNA sequencing was carried out in the bulk temporo-occipital cortex and in CD11b+ microglia acutely isolated from the whole brain. The expression of both *Apoe* and *Trem2* in cortical tissue and CD11b+ microglia did not differ between the A+/P^ki/ki^ and A+/P^wt/wt^ mice, suggesting that the DAM signature is not upregulated in APP/PS1 mice carrying the PLCγ2-P522R variant (Fig. 6B and C). In fact, no DEGs or enriched biological pathways were detected in the temporo-occipital cortex (Supplementary Figures, Figure S6D) or isolated CD11b+ microglia (Supplementary Figures, Figure S6E) when A+/P^ki/ki^ were compared with A+/P^wt/wt^ mice.

At the protein level, shedding of soluble TREM2 (sTREM2) terminates the signaling of full-length TREM2 and therefore influences microglial activation [18]. To assess whether PLCγ2-P522R modulates TREM2 shedding, we measured TREM2 levels in the PBS soluble fraction of temporo-occipital and hippocampal lysates. There were no differences between mice with different genotypes in the levels of sTREM2 in these brain areas (Supplementary Figures, Figure S6C), suggesting that TREM2 shedding is not promoted in the mice carrying the PLCγ2-P522R variant.

We next wanted to investigate whether the expression of genes related to cell motility and phagocytosis was altered in CD11b+ microglia isolated from female A+/P^ki/ki^ mice to understand the increased microglial area around β-amyloid plaques and the reduced pathology of β-amyloid plaques. More specifically, we examined the expression of fibronectin and integrin classes α and β genes, as described by Obst et al., which reported decreased cell adhesion and motility in human iPSC-derived macrophages upon PLCγ2 deficiency [50]. We also assessed other well-known cell motility and phagocytosis genes via gene set enrichment analysis (GSEA) core enrichment gene sets. Although several targets related to cell motility and phagocytosis were slightly upregulated in A+/P^ki/ki^ microglia compared with A+/P^wt/wt^ microglia, these results were not statistically significant (Supplementary Figures, Figure S7A and S7B).

### The PLCγ2-P522R variant promotes anxiety in female APP/PS1 mice carrying the PLCγ2-P522R variant

**Fig. 7:**
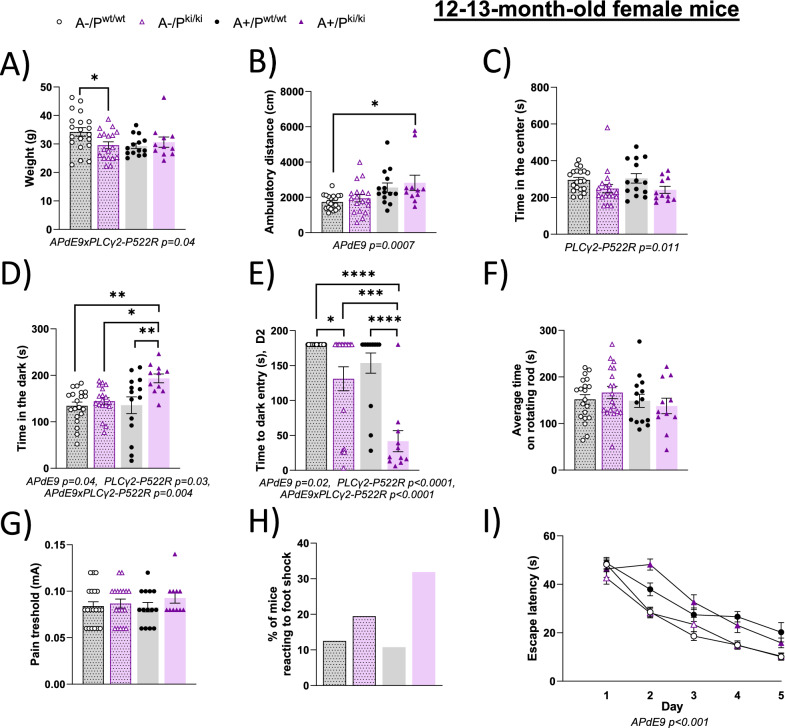
PLCγ2-P522R female mice exhibit avoidance responses to open places, bright environments, and sudden unpleasant stimuli. **A** Body weights of the PLCγ2-P522R (P^ki/ki^) mice are lower than those of their wild-type (P^wt/wt^) littermates on the APP/PS1-negative background (A-, *p = 0.024) but not on the APP/PS1 hemizygous background (A+). **B** PLCγ2-P522R does not influence the ambulatory distance traveled in the spontaneous activity test. **C** P^ki/ki^ mice spent significantly less time in the center of the novel test box than did control P^wt/wt^ mice, regardless of the APP/PS1 genotype. **D** In the passive avoidance test, the P^ki/ki^ mice showed increased avoidance of the illuminated chamber, which was further accentuated by the APP/PS1 hemizygous genotype. **E** Even after receiving a foot shock, P^ki/ki^ mice have a shorter latency to enter the dark chamber as compared to P^wt/wt^ mice. This tendency was further accentuated by the APP/PS1 hemizygous genotype. **F** No difference in motor skills was observed between the genotypes, as indicated by the time spent on the rotating rod. **G** P^ki/ki^ and P^wt/wt^ mice have similar thresholds for pain (foot shock), but **H)** P^ki/ki^ mice have a stronger behavioral response (crab-like walking backward) to electric foot shock. **I**) PLCγ2-P522R does not influence spatial learning or memory in the Morris water maze. The APP/PS1 hemizygous mice presented strongly impaired learning in terms of escape latency (F_1,64_ = 46.8, p < 0.001). A-/P^wt/wt^ n = 20, A-/P^ki/ki^ n = 18, A+/P^wt/wt^ n = 14, and A+/P^ki/ki^ n = 11. Two-way ANOVA with Šídák's multiple comparisons test. All the data are presented as the means ± SEMs. Each data point represents an individual mouse

Given that β-amyloid deposition and associated neuronal pathologies are mitigated in the brain tissue of APP/PS1 mice carrying PLCγ2-P522R, we next wanted to investigate whether this protective variant influences the behavioral alterations observed in APP/PS1 mice. First, the body weight showed an APP/PS1 x PLCγ2-P522R genotype interaction (p = 0.04), such that the APP/PS1 wild-type (-) A-/P^ki/ki^ mice had a lower weight than the A-/P^wt/wt^ littermates did (p = 0.024), but the same was not detected among the APP/PS1 hemizygous (+) mice (Fig. 7A). Given that the PLCγ2-P522R variant did not influence the ambulatory distance traveled in the spontaneous activity test (Fig. 7B), the observed weight reduction was not related to increased activity. However, the time spent in the center of the light–dark box was significantly shorter in the P^ki/ki^ than in the P^wt/wt^ mice, regardless of the APP/PS1 genotype (p = 0.011, Fig. 7C), suggesting that the PLCγ2-P522R variant increases the avoidance of open places. In the light‒dark box, the mice were given a free choice between compartment the illuminated and dark chamber. In this test, ANOVA revealed a main effect of both the PLCγ2-P522R (p = 0.03) and APP/PS1 (p = 0.04) genotypes and their interaction (p = 0.004). The P^ki/ki^ mice showed increased avoidance of the illuminated chamber, which was further accentuated in mice with the APP/PS1+ genotype (Fig. 7D). In the passive avoidance test, which was performed one day after the mice had received a foot shock when they entered the dark chamber, ANOVA revealed an even stronger main effect of both the PLCγ2-P522R (p < 0.0001) and APP/PS1 (p = 0.02) genotypes and their interaction (p < 0.0001). On the day after the foot shock, the P^ki/ki^ mice rushed into the dark chamber, whereas the P^wt/wt^ mice were hesitant to enter (Fig. 7E). This tendency was further accentuated by the APP/PS1+ genotype. Given that P^ki/ki^ and P^wt/wt^ mice did not differ in their motor abilities on the rotating rod (Fig. 7F) and that they had similar pain thresholds (Fig. 7G), this outcome was not explained by decreased motor ability or impaired ability to sense pain. In fact, the P^ki/ki^ mice had a stronger behavioral response (crab-like walking backward) to foot shock than did the P^wt/wt^ mice (Fig. 7H). Finally, the PLCγ2-P522R variant did not influence spatial learning or memory in the Morris water maze in either APP/PS1- or APP/PS1+ mice (Fig. 7I), whereas APP/PS1+ mice exhibited strongly impaired learning ability in terms of escape latency (p < 0.001). This finding suggests that rushing into the dark chamber behavior is not a result of memory dysfunction related to the foot shock in the P^ki/ki^ mice.

### The calcium signaling pathway, fatty acid metabolism, and oxidative phosphorylation-related pathways are activated in acutely isolated microglia from male mouse carrying the PLCγ2-P522R variant

**Fig. 8:**
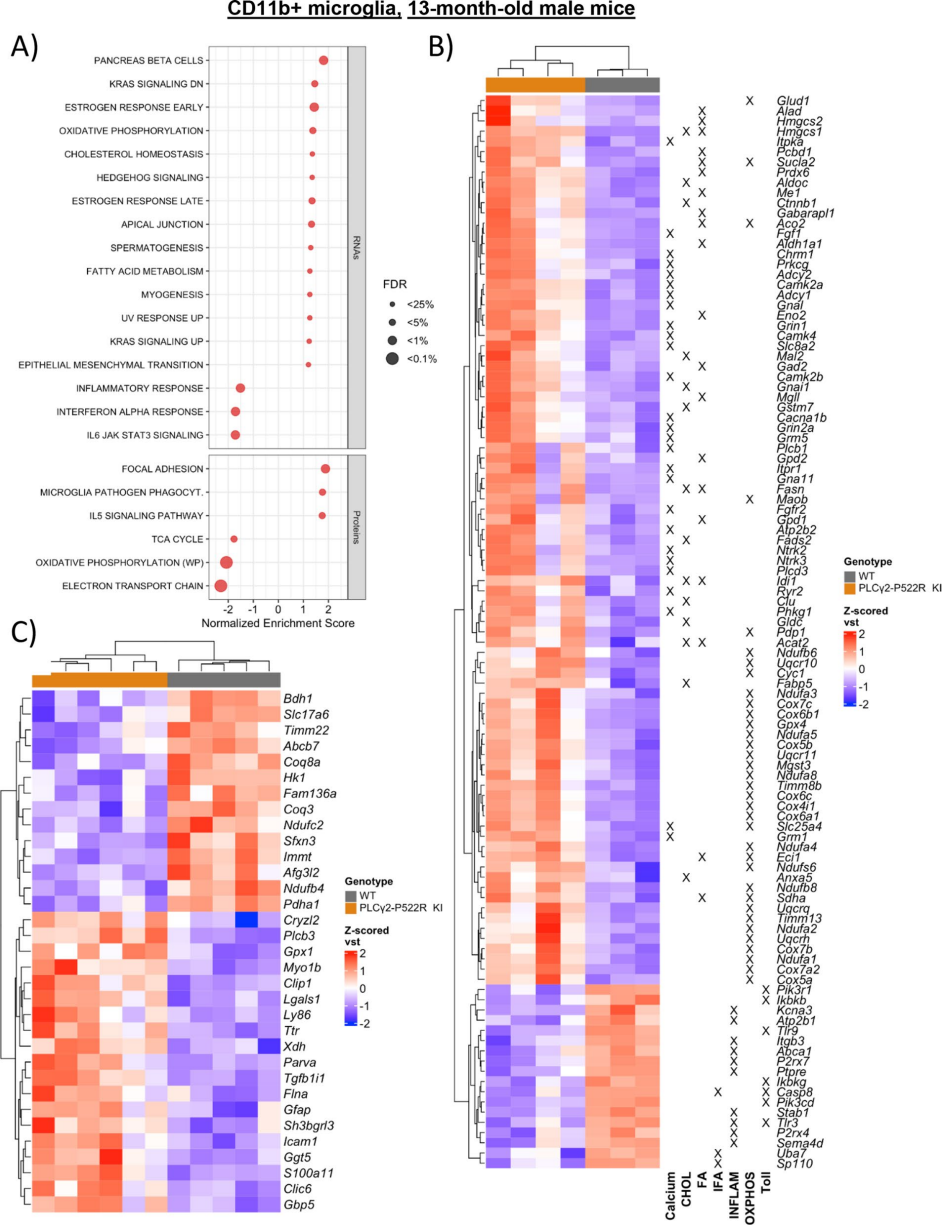
Fatty acid metabolism and mitochondrial function-related targets are upregulated in male mouse PLCγ2-P522R microglia. **A** Dot plot of normalized enrichment scores for enriched and depleted gene sets in enrichment analyses by GSEA for gene (MSigDB Hallmark, FDR < 0.25) and protein (Wikipathways, FDR < 0.05) expression in CD11b+ microglia isolated from 13-month-old PLCγ2-P522R KI and wild-type (WT) mice. **B** Heatmap of z scored vst-normalized expression of up- and downregulated differentially expressed genes (DEGs) that were core enrichment genes in a GSEA of gene sets for calcium signaling (Calcium), cholesterol homeostasis (CHOL), fatty acid metabolism (FA), the IFN-α response (IFA), the inflammatory response (INFLAM), oxidative phosphorylation (OXPHOS), and Toll-like receptor signaling (Toll). **C** Heatmap of z scored vs. normalized expression of significantly up- and downregulated differentially expressed proteins (DEPs, expressed as their encoding gene symbols) derived from a proteomics study. RNA WT n = 3 and PLCγ2-P522R KI n = 4; protein WT n = 5 and PLCγ2-P522R KI n = 6

To elucidate the molecular pathways associated with the PLCγ2-P522R variant, global RNA sequencing and proteomics analyses were performed in CD11b+ microglia. As the PLCγ2-P522R variant did not promote transcriptomic changes on an APP/PS1 background, the analyses were also carried out in microglia acutely isolated from 13-month-old male and female PLCγ2-P522R KI and WT mice. RNA sequencing revealed dramatically different RNA signatures between male PLCγ2-P522R KI and WT microglia, including 3719 DEGs (FDR 0.05) (Supplementary Figures, Figure S8A). Among these genes, 1302 were downregulated, and 2417 were upregulated. Gene enrichment analysis revealed several enriched biological pathways in the upregulated and downregulated DEGs between PLCγ2-P522R KI microglia and WT microglia (Fig. 8A). Among these pathways, calcium signaling, fatty acid (FA) metabolism, cholesterol homeostasis, and oxidative phosphorylation were prominently enriched in the upregulated DEGs in PLCγ2-P522R KI microglia, whereas pathways related to the inflammatory response and the interferon-α (IFN-α) response were enriched in the downregulated DEGs. Closer examination revealed that the core DEGs within the calcium signaling pathway included *Itpr1, Itpka, Camk4, Camk2a* and *Camk2b,* whereas the calcium-sensitive MEF2 transcription factor family members *Mef2a, Mef2c,* and *Mef2d* were downregulated in PLCγ2-P522R KI microglia (Fig. 8B; Supplementary Figures, Figure S8C). The upregulated genes within the FA metabolism pathway included, e.g., genes linked to LD lipolysis and FA synthesis (*Mgll, Fasn, Fads2, Fabp7, Fabp5, Fabp3,* Fig. 8B; Supplementary Figures, Figure S8C). Interestingly, several genes encoding PI3K subunits, *Pik3cd, Pik3cg, Pik3r5, Pik3ap1, Pik3r1,* and *Pik3r4,* were downregulated (Supplementary Figures, Figure S8C). Furthermore, several mitochondrial complex I (*Ndufa1, Ndufa2, Ndufc2, Ndufb8, Ndufs6, Ndufa4, Ndufa8, Ndufa5, Ndufa3,* and *Ndufb6*), complex II (*Sdha*), complex III (*Uqcrh, Uqcrq,* and *Uqcr10*), and complex IV (*Cox5a, Cox7a2, Cox7b, Cox6a1, Cox4i1, Cox6c, Cox5b, Cox6b1,* and *Cox7c*) genes as well as genes encoding other mitochondrial enzymes involved in FA oxidation (FAO, e.g., *Eci1*) were upregulated. Finally, the downregulated DEGs in PLCγ2-P522R KI microglia related to the inflammatory response, TLR signaling and the IFN-α response included *Tlr9, Tlr3, P2rx4, P2rx7,* and *Casp8* (Fig. 8B). Conversely, similar RNA-seq analysis of CD11b+ microglia from female mice did not yield as prominent results as microglia from male mice did (Supplementary Figures, Figure S8E). Thus, we decided to focus on assessing microglia isolated from males via proteomic analyses instead of microglia isolated from females to increase the likelihood of detecting significant changes owing to the PLCγ2-P522R variant.

Proteomic analysis revealed 34 differentially expressed proteins (DEPs, FDR 0.05), 14 of which were downregulated and 20 of which were upregulated in male PLCγ2-P522R KI microglia compared with WT microglia (Fig. 8C; Supplementary Figures, Figure S8B). DEPs and biological pathways were only mildly associated with changes detected in gene expression. In contrast to gene expression, core enriched proteins related to oxidative phosphorylation were downregulated in the proteomics dataset **(**Fig. 8C). In contrast, proteins related to FA oxidation were mildly upregulated in PLCγ2-P522R KI microglia, which was similar to what was observed at the gene expression level (Supplementary Figures, Figure S8D). Together, the results of the RNA and proteomic analyses suggest that pathways related to calcium signaling, the inflammatory/IFN response, mitochondrial oxidative metabolism (FAO), and lipid/FA metabolism are modulated in acutely isolated microglia from male mice carrying the PLCγ2-P522R variant.

### PLCγ2-P522R variant improves lipid processing in mouse primary microglia

**Fig. 9:**
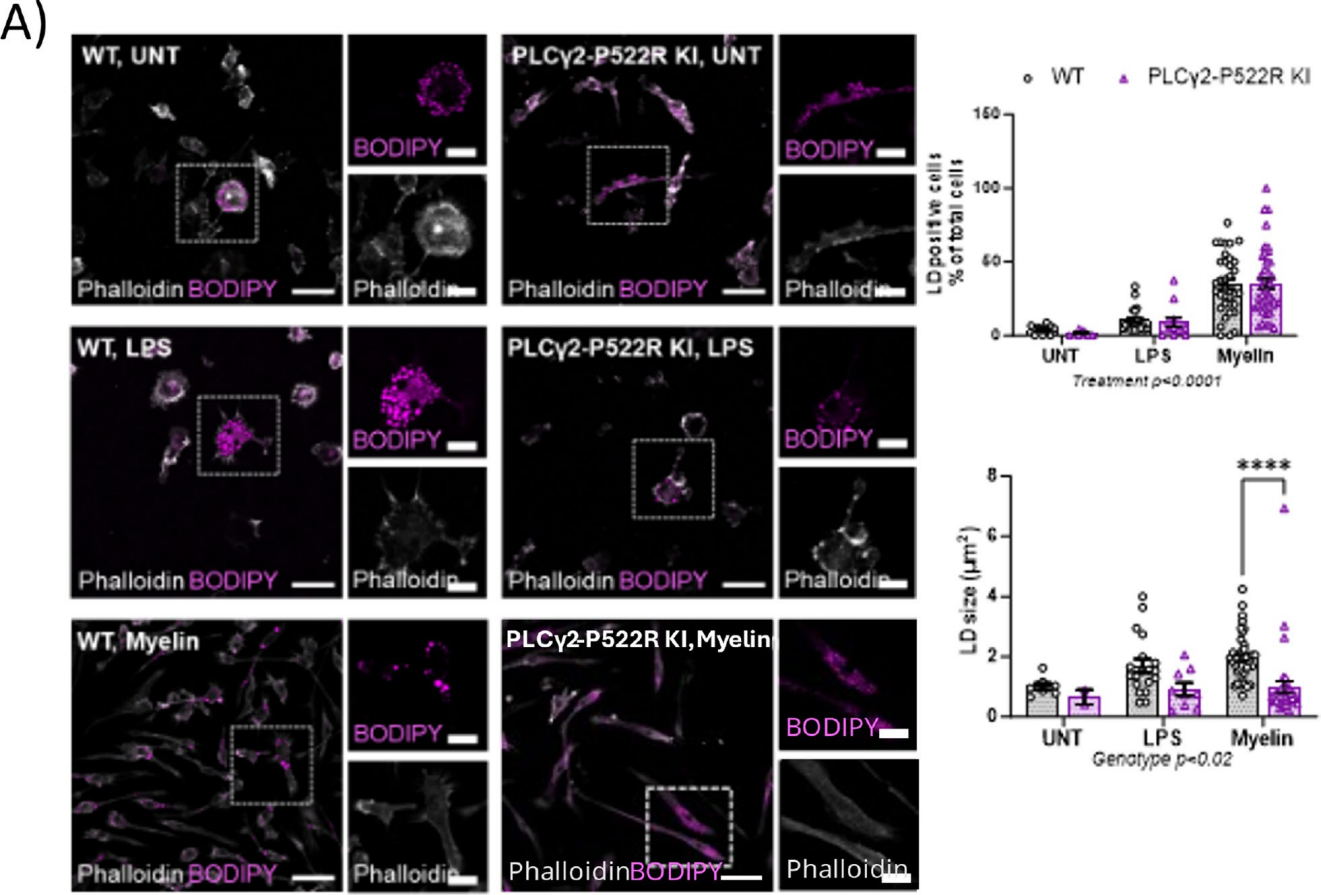
PLCγ2-P522R variant changes lipid accumulation in mouse primary microglia. **A** The percentage of lipid droplet (LD)-positive cells was greater in mouse primary microglia after 24 h of lipopolysaccharide (LPS) and 48 h of myelin treatment than in untreated cells (UNT). The percentage of LD-positive cells did not differ between wild-type (WT) and PLCγ2-P522R knock-in (KI) microglia. PLCγ2-P522R KI decreases the size (µm^2^) of individual LDs under myelin-treated conditions (****p < 0.0001). n (analyzed images) WT UNT n = 11, LPS n = 22, myelin n = 42; PLCγ2-P522R KI UNT n = 6, LPS n = 12, myelin n = 35. Two-way ANOVA with Tukey’s multiple comparisons test. All the data are presented as the means ± SEMs

The downregulation of genes involved in LD dynamics, including *FABPs*, has been shown to be associated with lipid accumulation and impaired mitochondrial function in TREM2 KO and PLCγ2 KO iMGLs [6, 8]. As several genes associated with LD accumulation (*Pi3k* subunits) and LD lipolysis (e.g., *Fabp*) were differentially expressed in CD11b+ PLCγ2-P522R KI mouse microglia (Supplementary Figures, Figure S8C), we next addressed whether the PLCγ2-P522R variant influences LD accumulation under LPS- and myelin-induced stress conditions. Both LPS and myelin have previously been shown to cause lipid dyshomeostasis and promote LD accumulation in similar models [10, 51]. Compared with no treatment, LPS and even more myelin treatment increased the number of LD-positive microglia, whereas genotype had no effect on the percentage of LD-accumulating microglia (Fig. 9). However, the size of the LDs was clearly smaller in PLCγ2-P522R KI microglia than in WT microglia (p < 0.0001) upon myelin treatment. A similar but nonsignificant trend was also detected in LPS-treated microglia. These findings suggest that PLCγ2-P522R KI reduces the buildup of LDs in microglia under conditions of cellular stress.

### PLCγ2-P522R variant improves mitochondrial function in human microglial models

**Fig. 10:**
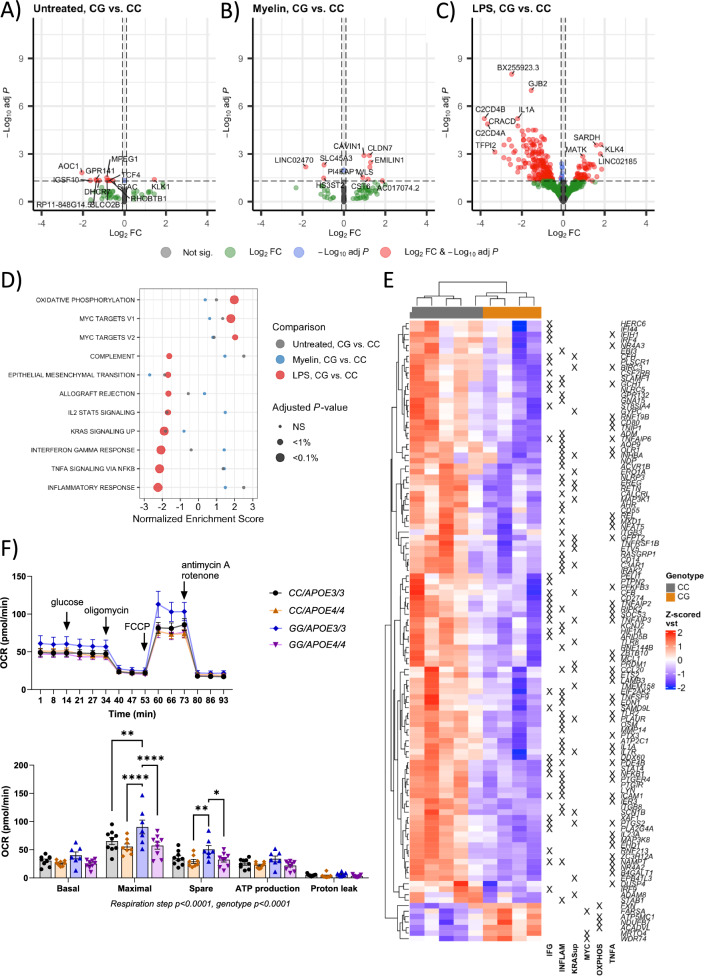
PLCγ2-P522R variant improves mitochondrial function in human microglial models. Volcano plot of differentially expressed genes (DEGs) in blood monocyte-derived microglia-like cells (MDMi) from PLCγ2-P522R variant carriers (CG) and matched controls (CC) under **A**) untreated (UNT), **B** myelin-treated and **C** lipopolysaccharide (LPS)-treated conditions. FDR < 0.05. Horizontal dashed line: adjusted p value of 0.05; vertical dashed lines: |log2FC|= 0.1. **D** Dot plot of normalized enrichment scores for enriched and depleted gene sets in enrichment analyses by GSEA for gene (hallmarks, FDR < 0.25) expression in MDMi cells of CG and CC individuals upon UNT, LPS, and myelin treatments. **E** Heatmap of z scored vst-normalized expression of up- and downregulated DEGs in the LPS-treated condition that were core enrichment genes in a GSEA of gene sets for the IFN-γ response (IFG), inflammatory response (INFLAM), Kras signaling up (KRASup), MYC-target (MYC), oxidative phosphorylation (OXPHOS), and TNF-α signaling (TNFA). UNT CC n = 5 and CG n = 4; Myelin CC n = 5 and CG n = 4; LPS CC n = 5 and CG n = 4. **F**) The mitochondrial oxygen consumption rate (OCR, maximal respiration and spare respiratory capacity) is greater in homozygous PLCγ2-P522R (GG)-induced pluripotent stem cell-derived microglia (iMGL) than in isogenic controls (CCs) with the *APOE3/3* genetic background but not the *APOE4/4* genetic background. iMGL n = 1 line per group, 7–9 technical replicates per line. Two-way ANOVA with Tukey’s multiple comparisons test. The data are presented as the means ± SEMs

To assess whether PLCγ2-P522R-driven mechanisms are translated into human microglial models, we conducted RNA sequencing of human MDMi cells derived from PLCγ2-P522R variant carriers (CG) and matched control individuals (CC) under untreated conditions and after myelin and LPS treatments. Compared with those in CC MDMi cells, only 11 significantly altered DEGs were identified in the untreated group, and 10 DEGs were identified in the myelin-treated group in the CG (Fig. 10A-B). LPS had the most robust effect on gene expression, resulting in 236 DEGs in CG cells compared with CC cells (Fig. 10C). Under LPS-treated conditions, gene enrichment analysis revealed the enrichment of oxidative phosphorylation and MYC targets (V1 and V2) in the upregulated DEGs, whereas pathways related to the inflammatory response, TNF-α signaling, the IFN-γ response, and KRAS signaling were most clearly enriched pathways in the downregulated DEGs in the MDMis of CG carriers (Fig. 10D). The core enriched genes in the oxidative phosphorylation pathway included *ACADVL,* an enzyme regulating FA oxidation; the mitochondrial complex I gene; *NDUFB7;* mitochondrial ATP synthase; *ATP5MC1*; and *FXN,* a gene associated with mitochondrial iron transport and respiration (Fig. 10E). Upregulated genes linked to the MYC target pathway and involved in RNA regulation included *FARSA, MRTO4,* and *WDR74*. Several IFN (*IRF9, IRF4, and STAT4*), TNF (*TNFAIP2, TNFAIP3, and TNFAIP6*) and IL (*IL1-α and IL-7R*) family members, immune sensors (*CD14, TLR8, TLR2, and NLRP3*), and related transcription factors (*NFKB1 and NFAT5*) were downregulated and associated with the enriched pathways. To examine whether a reduction in inflammatory cytokine expression can be detected at the protein level, IL-6 and TNF-α levels were measured in the conditioned medium of LPS-treated MDMi cells. However, no significant changes were found in the levels of either of the cytokines (Supplementary Figures, Figure S9A). Together with findings in mouse CD11b+ microglia, these data confirm a central role for PLCγ2-P522R in the regulation of genes associated with mitochondrial function and FA oxidation as well as inflammatory/IFN responses.

To further investigate mitochondrial function in human microglia, we employed PLCγ2-P522R homozygous (GG) iMGLs and isogenic control lines (CCs) with *APOE3/3* or *APOE4/4* backgrounds (Supplementary Figures, Figure S9B-C). In general, the mitochondrial OCR significantly differed between the genotypes (p < 0.0001). Specifically, maximal respiration was greater in PLCγ2-P522R *GG*/*APOE3/3* iMGLs than in all the other groups (*CC/APOE3/3* p = 0.001, *CC/APOE4/4* p < 0.0001, *CG/APOE4* p < 0.0001) (Fig. 10F). In addition, the spare respiratory capacity was significantly greater in *GG*/*APOE3/3* cells than in *CC/APOE4/4* (p = 0.009) and *GG/APOE4/4* (p = 0.027) iMGLs. However, PLCγ2-P522R did not increase mitochondrial respiration in the *APOE4/4* background. Taken together, both the mouse and human data in this study point toward promoted lipid/FA metabolism and improved mitochondrial function in microglia from individuals carrying PLCγ2-P522R variant.

## Discussion

PLCγ2-P522R is a well-established variant that reduces the risk for LOAD and was recently shown to mitigate β-amyloid pathology in the 5XFAD mouse model of AD [2, 14, 17]. In line with these findings, we now demonstrate that PLCγ2-P522R variant decreases the β-amyloid plaque count and coverage in the brain tissue of female APP/PS1 mice, which is a less aggressive mouse model of AD as compared to 5XFAD mouse model. This decrease was associated with increased microglial activation and microglial area around β-amyloid plaques as well as decreased formation of β-amyloid plaque-associated neuronal dystrophy, as shown by 22C11 IHC staining. Importantly, at the mechanistic level, the PLCγ2-P522R variant upregulated pathways related to lipid/FA metabolism and mitochondrial function in several different PLCγ2-P522R mouse and human microglial models. Conversely, signaling pathways related to the inflammatory response were suppressed in these models.

In 5XFAD mice carrying the PLCγ2-P522R variant, the size of β-amyloid plaques was reduced and the plaque morphology shifted from diffuse to a more compacted form [17]. In contrast, another study revealed no differences in the β-amyloid plaque load or microglial internalization of β-amyloid in 5XFAD mice transplanted with human PLCγ2-P522R iMGLs [16], highlighting the importance of additional studies in different models to comprehensively characterize the role of the PLCγ2-P522R variant in the cellular processes relevant for AD. In the current study, the number of X-34-positive compact plaques was reduced in the brain tissues of 13-month-old female APP/PS1 mice carrying the PLCγ2-P522R variant. Instead, the ratio of 6E10-positive diffuse plaques and X-34-positive compact plaques as well as the sizes of the individual plaques and the levels of soluble and insoluble Aβ remained unaffected. Furthermore, in contrast to what was reported in 5XFAD mice [17], the PLCγ2-P522R variant did not change the colocalization of 6E10 and IBA1, suggesting that the uptake of diffuse β-amyloid remained unaltered in these mice. This discrepancy may be related to the fact that the APP/PS1 mice in the present study were analyzed at the age of 13 months, which contrasts with the results of the analyses of 7.5-month-old 5XFAD mice. By this age, APP/PS1 mice are typically at an advanced stage of pathology, with extensive β-amyloid plaque deposition, whereas 7.5-month-old 5XFAD mice may exhibit different pathological features, such as early plaque formation and initial microglial responses. Despite this, we detected a significant increase in the area of microglia around β-amyloid plaques, which was accompanied by a reduced number of dystrophic neurites, indicative of decreased β-amyloid-driven axonal damage [44–47]. Importantly, we did not detect significant changes in other well-established markers of neuronal dystrophy, such as the p-Tau/total Tau ratio in the tissue lysates we studied. Thus, more detailed morphological analyses are needed to conclusively determine the beneficial effects of the PLCγ2-P522R variant on synaptic, axonal and neuronal pathologies under β-amyloid-driven conditions.

In recent years, a critical role for transcriptional changes in microglial activation has been highlighted, and unique RNA signatures are used to identify distinct microglial subpopulations [5, 52]. Accordingly, specific RNA signatures highlighting antigen presentation or microglial activation and responsiveness to β-amyloid pathology have been described in PLCγ2-P522R chimeric and endogenous 5XFAD models, respectively [16, 17]. To our surprise, the PLCγ2-P522R variant did not promote transcriptional changes in the bulk cortex or in isolated CD11b+ microglia of APP/PS1 mice. One possible reason for this outcome is that single-cell resolution or spatial analyses focusing on plaque-associated microglia may be needed to reveal potentially subtle expression changes induced by the PLCγ2-P522R variant in the APP/PS1 model. Compared with the APP/PS1 model, which has a milder phenotype, the 5XFAD model is known for its aggressive β-amyloid pathology, which may elicit stronger microglial responses and more pronounced transcriptional changes. Furthermore, RNA sequencing was carried out at different ages in APP/PS1 (13-month-old) and 5XFAD (7.5-month-old) mice, which may represent different stages of pathologies and microglial responses analyzed in these studies.

Deficiency of PLCγ2 in human iPSC-derived macrophages leads to reduced migration and adhesion, which is associated with decreased expression of fibronectin and a specific set of integrin genes [50]. Given these findings, we assessed the expression status of these genes as well as other relevant genes linked to cell motility in acutely isolated microglia from 13-month-old female A+/P^ki/ki^ and A+/P^wt/wt^ mice. Although we did not observe significant changes in the pathway enrichment analysis, several genes linked to cell motility showed a subtle increase in their expression in PLCγ2-P522R microglia. In line with this observation, proteomics analysis of microglia acutely isolated from 13-month-old male PLCγ2-P522R KI and wild-type mice without the APP/PS1 background revealed significant upregulation of focal adhesion-associated proteins in PLCγ2-P522R microglia. Collectively, these findings suggest that the observed increase in the area of microglia around β-amyloid plaques in female A+/P^ki/ki^ mice could be due to the increased clustering of activated microglia around β-amyloid plaques. Relatedly, previous studies have demonstrated that the PLCγ2-P522R variant also increases the phagocytosis of Aβ 1–42 in both mouse microglia and human iPSC models [17, 53, 54]. To address this aspect, we assessed the expression status of genes linked to microglial phagocytosis in acutely isolated microglia from 13-month-old female A+/P^ki/ki^ and A+/P^wt/wt^ mice. This analysis also revealed a subtle increase in the expression of phagocytosis genes in PLCγ2-P522R microglia, although this change was not statistically significant in the pathway enrichment analysis. Similarly, proteomics analysis of microglia acutely isolated from 13-month-old male PLCγ2-P522R and wild-type mice without the APP/PS1 background revealed the upregulation of genes involved in microglial phagocytosis of pathogens in PLCγ2-P522R microglia. Together with increased microglial PET signals in the brain tissue of 13-month-old female mice, these findings suggest that the PLCγ2-P522R variant enhances microglial activation and shifts microglia to a more β-amyloid-responsive state, including increased phagocytotic activity of Aβ. This then leads to enhanced barrier formation around β-amyloid plaques, which protects the surrounding neuronal population. Thus, the protective PLCγ2-P522R variant may exert similar beneficial effects on the functions of microglia as those previously reported with a TREM2-activating antibody in different AD disease models [8]. Importantly, our current findings are primarily based on female A+/P^ki/ki^ mice. In contrast, omics-based analyses of male PLCγ2-P522R microglia without the APP/PS1 background revealed more pronounced effects on PLCγ2-P522R microglia than female PLCγ2-P522R microglia. This raises the question of potential sex-dependent differences in immune cell functions related to the PLCγ2-P522R variant, indicating the need for further studies in male A+/P^ki/ki^ mice. This notion is also supported by a recent study that revealed that the PLCγ2-P522R variant modulates mouse peripheral macrophage functions in a sex-dimorphic manner [55].

Although PLCγ2-P522R reduced β-amyloid deposition and associated neuronal pathologies, there was no evident improvement in learning and memory functions in the APP/PS1 mice. The most robust manifestation of the behavioral phenotype associated with PLCγ2-P522R was observed in the passive avoidance task. The tendency of PLCγ2-P522R mice to rush into the dark despite having mild foot shock a day earlier can stem from changes in four CNS systems that regulate 1) the pain threshold, 2) spontaneous motor activity, 3) memory, and 4) the level of anxiety. The threshold to react to an electric foot shock did not differ between the genotypes. The total distance traversed in a new test box also did not differ. The ‘gold standard’ spatial memory test, the Morris water maze test, revealed robust impairment associated with the APP/PS1 genotype but no effect of PLCγ2-P522R. When these issues are excluded, changes in anxiety level remain the most plausible explanation for the passive avoidance test results. This interpretation is supported by direct evidence of increased anxiety in PLCγ2-P522R mice. First, before passive avoidance memory testing, we observed that the PLCγ2-P522R mice spent significantly less time in the illuminated chamber than the other groups did. Second, after receiving foot shock, PLCγ2-P522R mice walked backward in a crab-like manner, which can be interpreted as an attempt to retreat from the daunting environment. Third, during activity monitoring, the PLCγ2-P522R mice avoided the center of the arena more than the control mice did. Fear and anxiety are essential emotions that aid in the survival of species such as mice living under constant threat from predators in the wild [56]. Furthermore, increasing evidence has shown that microglial activation may be a key driver of the anxiety phenotype in mice [57]. Together, these findings suggest that the PLCγ2-P522R variant promotes an anxiety phenotype in APP/PS1 mice, which may promote longevity via protection against environmental threats through mechanisms associated with altered microglial functions.

We previously showed that PLCγ2-P522R KI mouse macrophages respond more efficiently to LPS- and IFNγ-induced inflammation than wild-type cells do [7]. Accordingly, PLCγ2 KO inhibited the LPS-induced inflammatory response in human iMGLs [6], suggesting that PLCγ2 is central for TLR-mediated acute inflammatory signaling. In the present study, RNA sequencing revealed the downregulation of inflammatory and interferon signaling pathways and a similar trend in secreted IL-6 and TNF-α levels in human MDMi cells after LPS treatment. The different cell types and/or varying LPS doses used in these studies may at least partially explain the downregulation of the inflammatory response in MDMi cells. In line with the MDMi data, the PLCγ2-P522R variant slightly reduced inflammatory cytokine levels in the temporo-occipital cortex and hippocampus of APP/PS1 mice and led to suppression of inflammatory and interferon signaling pathways in CD11b+ microglia isolated from 13-month-old mice without AD-associated stress. These findings suggest that the PLCγ2-P522R variant suppresses the proinflammatory activation associated with chronic β-amyloid deposition (APP/PS1 mice), aging (CD11b+ microglia) or a low inflammatory stimulus (MDMi). Overall, the protective PLCγ2-P522R variant is most likely centrally involved in the regulation of the inflammatory response, although the effect size and direction may be context dependent.

Microglia rely on the flexible use of diverse energy sources, including FAs, to perform numerous immune functions efficiently and to maintain tissue homeostasis [58]. Under energy-demanding conditions, FAs are released from their main intracellular lipid storage units, known as LDs, which mainly contain triglycerides and cholesterol esters. Free FAs are then transported to mitochondria, where they are used to fuel mitochondrial oxidative phosphorylation in a process called FA β-oxidation (FAO) [58]. Conversely, aged or stressed microglia present a buildup of intracellular LDs, deterioration of mitochondrial efficiency, and downregulation of oxidative phosphorylation-related genes [59, 60]. Reduced accumulation of LDs was observed in the present study in PLCγ2-P522R KI mouse microglia upon myelin and, to a lesser extent, LPS treatment, suggesting that the PLCγ2-P522R variant enhances lipid metabolism under different stress conditions. Accordingly, several DEGs (e.g., *Fabp3, Fabp5, Fabp7, Mgll, and Eci1*) and biological pathways (β-oxidation and FAO) identified from RNA sequencing and proteomics data directly indicate enhanced lipid metabolism and FAO in acutely isolated adult PLCγ2-P522R mouse microglia. In line with the present findings, depletion of either TREM2 or PLCγ2 has previously been shown to increase lipid accumulation and impair mitochondrial respiration via the suppression of lipid regulatory genes, including *FABP5* [6, 8, 61]. Here, several genes encoding fatty acid-binding proteins (Fabp3, Fabp5, and Fabp7), which play a role in lipid droplet lipolysis and the transport of free fatty acids to mitochondria for energy production [62], were found to be upregulated in PLCγ2-P522R mouse microglia. Additionally, the gene *Mgll*, which encodes monoacylglycerol lipase responsible for the final step in triglyceride hydrolysis to free fatty acids [63], along with *Eci1*, an enoyl-CoA delta isomerase 1 involved in mitochondrial fatty acid oxidation, was also upregulated in PLCγ2-P522R mouse microglia. PI3K-AKT signaling is another key regulator of lipid metabolism, and PI3K inhibitors have been shown to efficiently block the accumulation of LDs in numerous studies and in several study models [9, 64, 65]. In this context, we have previously shown that AKT activation is decreased in the PLCγ2-P522R mouse brain, possibly due to increased consumption of PIP_2_ by PLCγ2 and consequently reduced PIP_3_ resources for promoting PI3K-AKT signaling [7]. In line with this finding, RNA sequencing revealed that several genes encoding PI3K subunits were downregulated in adult PLCγ2-P522R KI mouse microglia compared with WT microglia. Although further studies are needed to confirm the connection between the in vitro and in vivo findings, it is possible that the reduced accumulation of LDs observed in the present study is at least partially mediated via reduced lipid storage due to the inhibition of PI3K-AKT signaling and/or increased LD lipolysis due to the increased expression of *Fabps*.

Importantly, these observations were further supported by transcriptomic and functional evidence from human microglial models, which revealed the upregulation of oxidative phosphorylation, FAO, and mitochondrial respiration-related genes in MDMi cells from PLCγ2-P522R variant carriers and increased mitochondrial respiration in homozygous PLCγ2-P522R iMGL lines with *APOE*3/3 but not in those from the *APOE*4/4 background. These findings indicate that the protective PLCγ2-P522R variant cannot fully counteract the negative effects of *APOE*4 on mitochondrial respiration. However, additional research is necessary to explore whether other aspects of mitochondrial function, such as FAO, are influenced by the PLCγ2-P522R variant and whether it might help mitigate the well-documented disruptions in mitochondrial homeostasis caused by *APOE*4. Taken together, the transcriptomics and functional observations in the mouse and human models point toward increased mitochondrial functions, FAO, and lipid metabolism and thus suggest that the protective PLCγ2-P522R variant may be associated with beneficial effects against age- and/or stress-related functional deterioration of microglia.

There are several hypermorphic variants in *PLCG2* that have been associated with autoimmune disease and cancer [58–61, 66]. In this context, the PLCγ2-P522R variant is a mild hypermorph, which only slightly increases the enzymatic activity of PLCγ2 compared with other hypermorphic variants, such as PLCγ2-S707Y, which is associated with severe autoinflammatory symptoms and immune dysregulation [66]. Importantly, the PLCγ2-P522R variant has not been associated with any autoimmune diseases or cancers in previous studies, highlighting its beneficial effects on immune cells. Furthermore, it was recently discovered that rare loss-of-function variants in *PLCG2* increase AD risk via nonsense-mediated mRNA decay [15], suggesting that the degree of enzymatic activity of PLCγ2 is the key determinant of whether *PLCG2* variants mediate risk-increasing or protective effects on AD or other diseases. Therefore, additional studies focusing on the proper PLCγ2 activity range are needed to comprehensively understand its impact on cellular functions to be further applied in potential therapeutic approaches. In this context, however, drug candidates that target PLCγ2 should mimic the effects of the PLCγ2-P522R variant on microglial functions.

## Conclusions

Our present study suggested that β-amyloid deposition and associated neuronal pathologies are reduced in APP/PS1 model mice carrying the AD-protective PLCγ2-P522R variant as a result of increased microglial activation and clearance of β-amyloid deposits while suppressing their proinflammatory phenotype. These protective changes are likely mechanistically linked to increased lipid/FA metabolism and mitochondrial functions, which have been observed in different mouse and human microglial models. Although further studies are warranted, our findings suggest that the protective effects of the PLCγ2-P522R variant upon AD-associated stress could be a consequence of slower metabolic deterioration related to microglial aging.

## Supplementary Information

Additional file 1

Additional file 2

Additional file 3

Additional file 4

Additional file 5

## Data Availability

Dataset generated and/or analyzed in the current study are included within the article and its additional files. The RNA sequencing datasets of mouse cortical tissue and isolated CD11b+ microglia are available in the Gene Expression Omnibus (NCBI) repository, GSE277167, https://www.ncbi.nlm.nih.gov/geo/query/acc.cgi?acc = GSE277167. The study participant consent does not allow opening the sequencing data of the carrier-derived MDMi cells which was generated and analyzed during the current study, but they are available from the corresponding authors (MT or MH) on a reasonable request.

## Abbreviations

A+/P^ki/ki^: APP/PS1 x PLCγ2-P522R KI
A+/P^wt/wt^: APP/PS1
AD: Alzheimer’s disease
APP/PS1: APPswe/PS1dE9 transgenic mouse line
DAG: Diacylglycerol
DAM: Disease-associated microglia
DEGs: Differentially expressed genes
DEPs: Differentially expressed proteins
DIA-PASEF: Data independent acquisition parallel accumulation–serial fragmentation
ECAR: Extracellular acidification rate
FAO: Fatty acid oxidation
GFAP: Glial fibrillary acidic protein
IFN-α: Interferon-α
iMGL: Induced pluripotent stem cell-derived microglia
IP3: Inositol trisphosphate
KI: Knock-in
KO: Knock-out
LD: Lipid droplets
LDH: Lactate dehydrogenase
LFQ: Label-free quantification
LOAD: Late-onset Alzheimer’s disease
LPS: Lipopolysaccharide
MDMi: Monocyte-derived microglia
OCR: Oxygen consumption rate
OSEM: Ordered-subset expectation maximization
PBMC: Peripheral blood mononuclear cells
PET: Position emission tomography
PIP2: Phosphatidylinositol 4,5-bisphosphate
PLCγ2-P522R: Phospholipase C gamma 2, proline 522 to arginine
ROIs: Regions of interest
sTREM2: Soluble TREM2
TGF-β: Transforming factor beta
TIMS: Trapped Ion Mobility Spectrometry
TLR: Toll-like receptor
TREM2: Triggering receptor expressed on myeloid cells 2
TSPO: Mitochondrional translocator protein 18 kDa
WT: Wild-type
