# Supplementary material for: The protective PLCγ2-P522R variant mitigates Alzheimer’s disease-associated pathologies by enhancing beneficial microglial functions
Source: J Neuroinflammation. 2025 Mar 5;22:64. doi: 10.1186/s12974-025-03387-6 (PMC11881468; doi:10.1186/s12974-025-03387-6)

Uncut Western blot images of APP and its metabolites in A) temporo-occipital cortex and B) hippocampus of the APP/PS1xPlcγ2-P522R ($A+/P^{ki/ki}$) and APP/PS1 ($A+/P^{wt/wt}$) mice. Red X indicates a sample unrelated to the project.

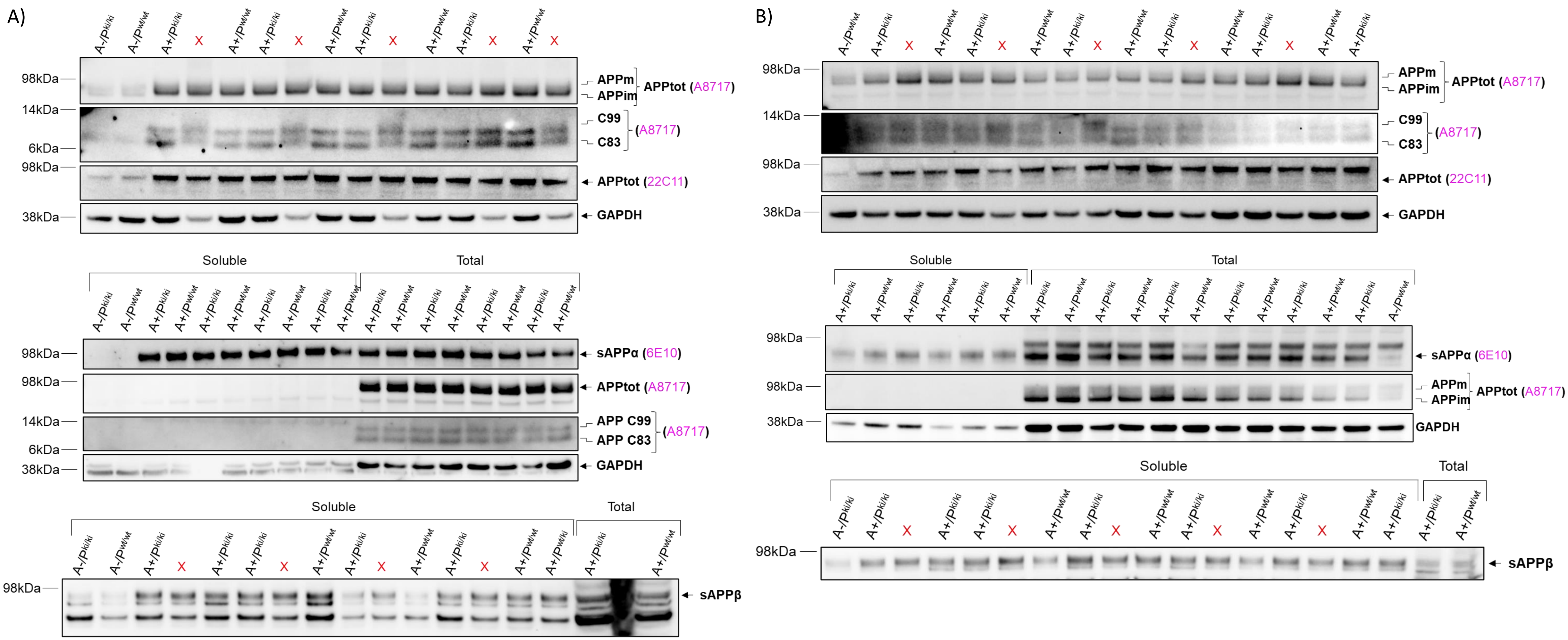

Uncut Western blot images of Tau and p-Tau in A) temporo-occipital cortex and B) hippocampus of the APP/PS1xPlcγ2-P522R (A+/$P^{ki/ki}$) and APP/PS1 (A+/$P^{wt/wt}$) female mice.

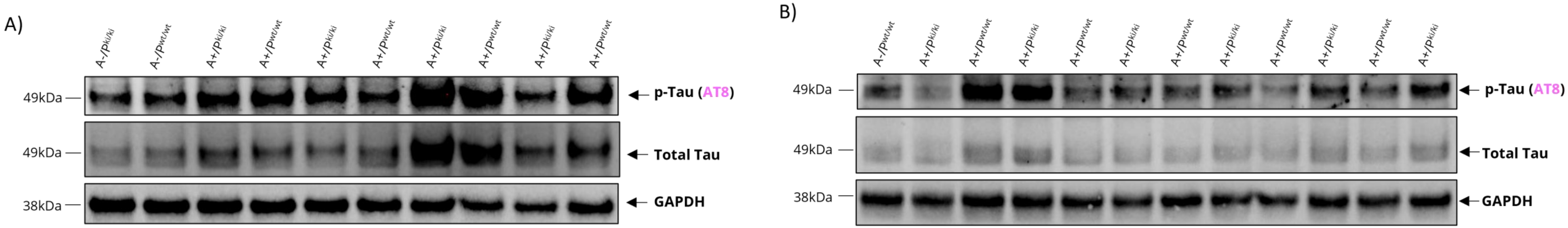

F VC Blot III 6E10 prime 60 s

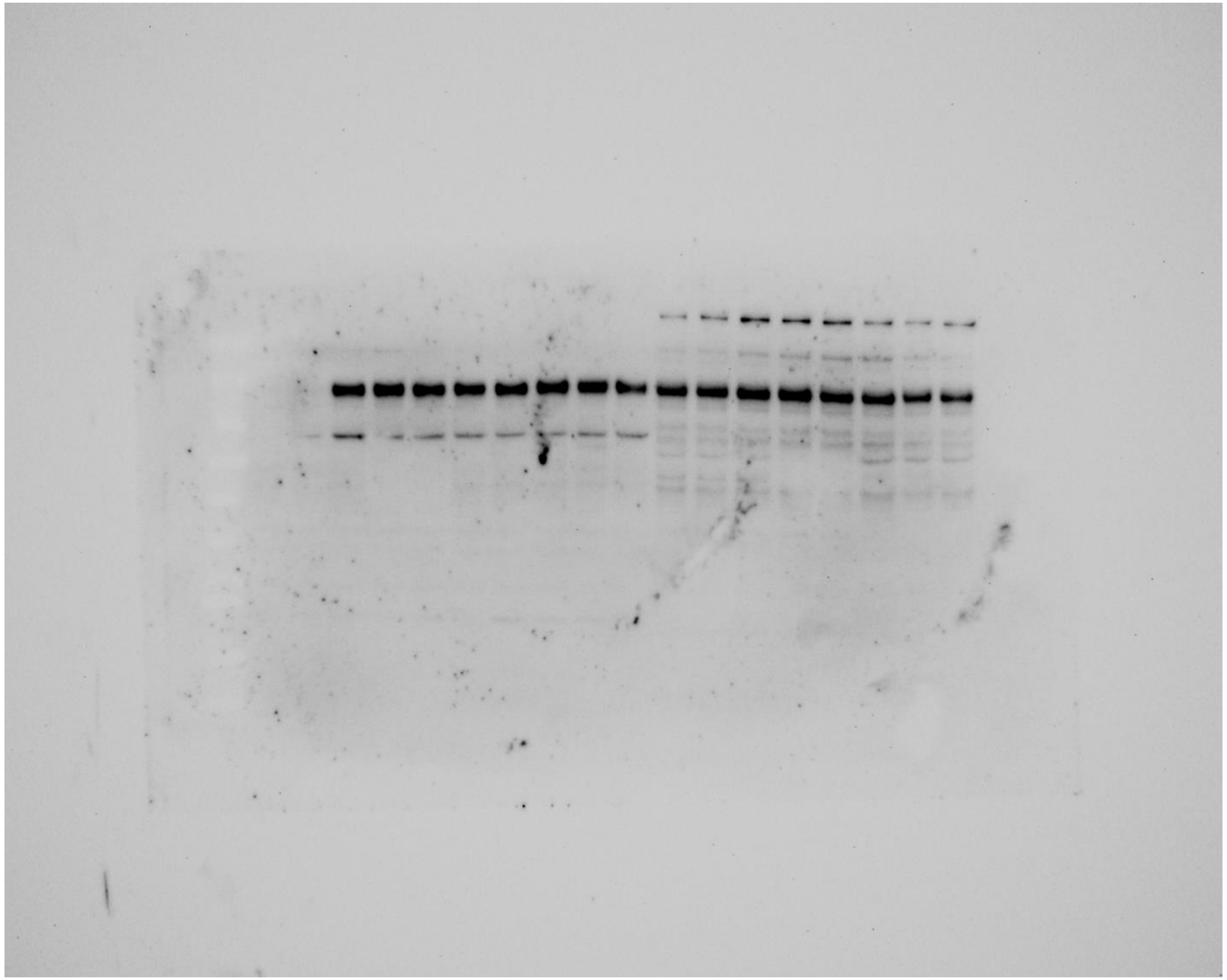

F VC Blot III gapdh prime 60 s

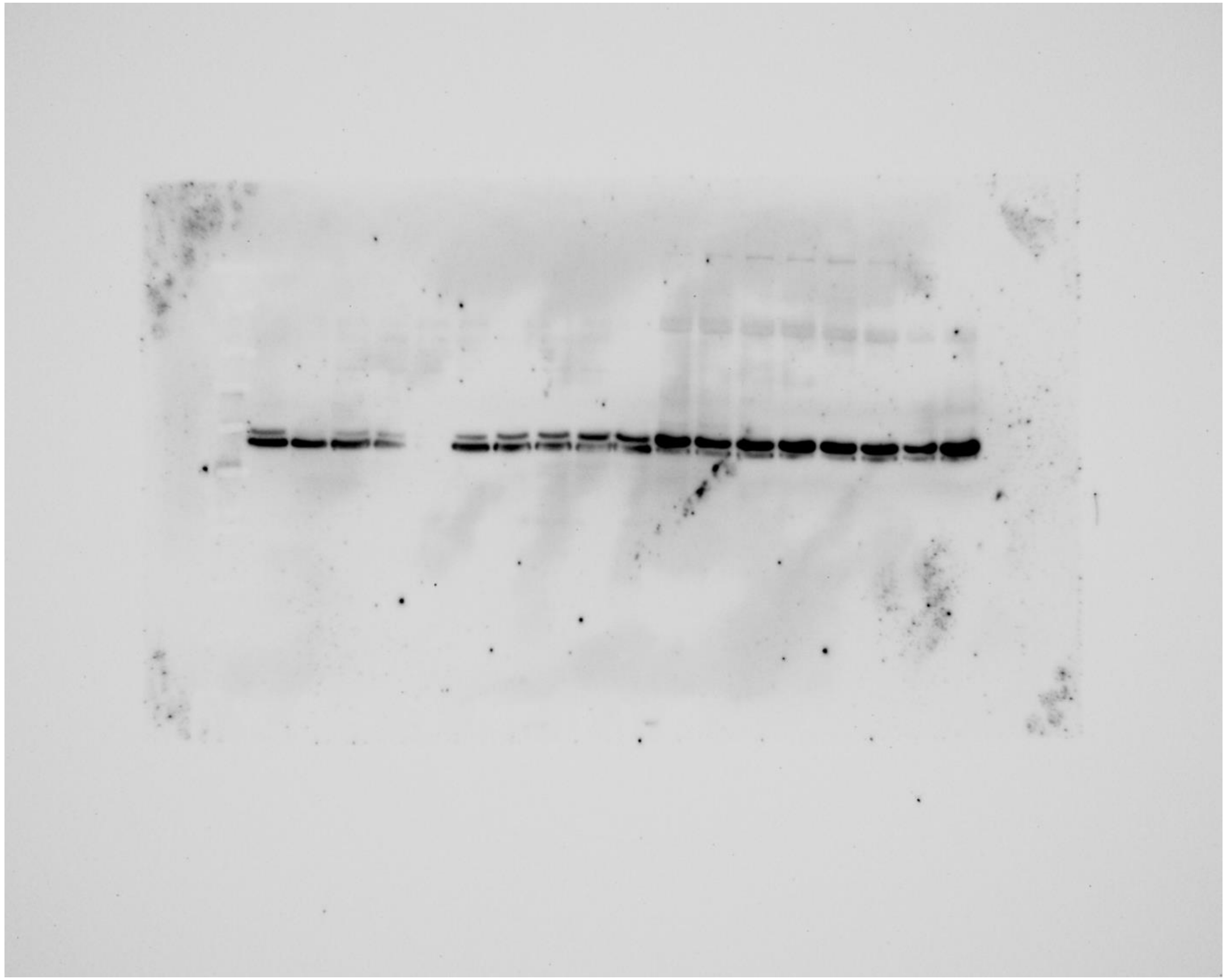

F VC Blot III A8717 prime 30 s

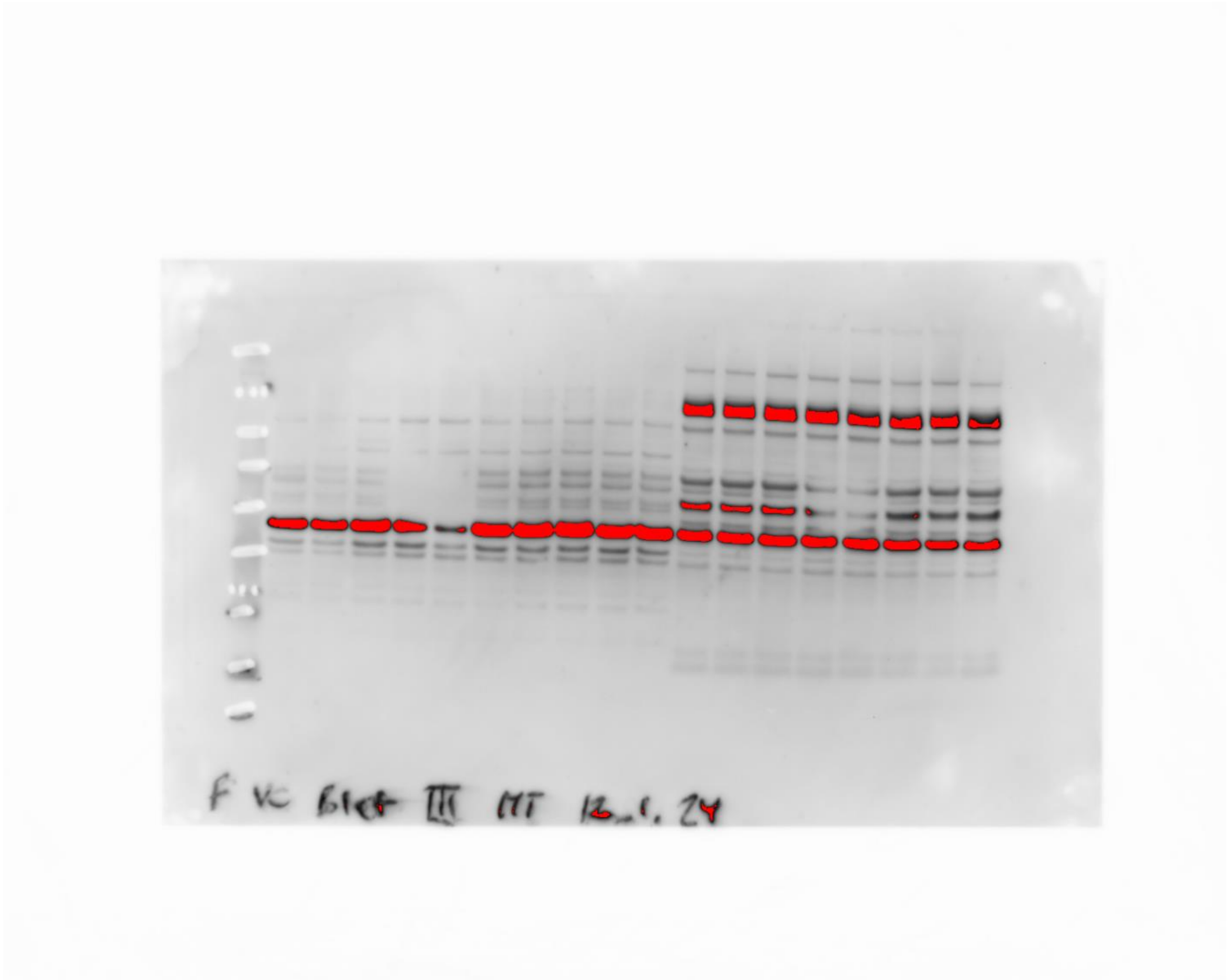

F VC Blot III A8717 select 30 s

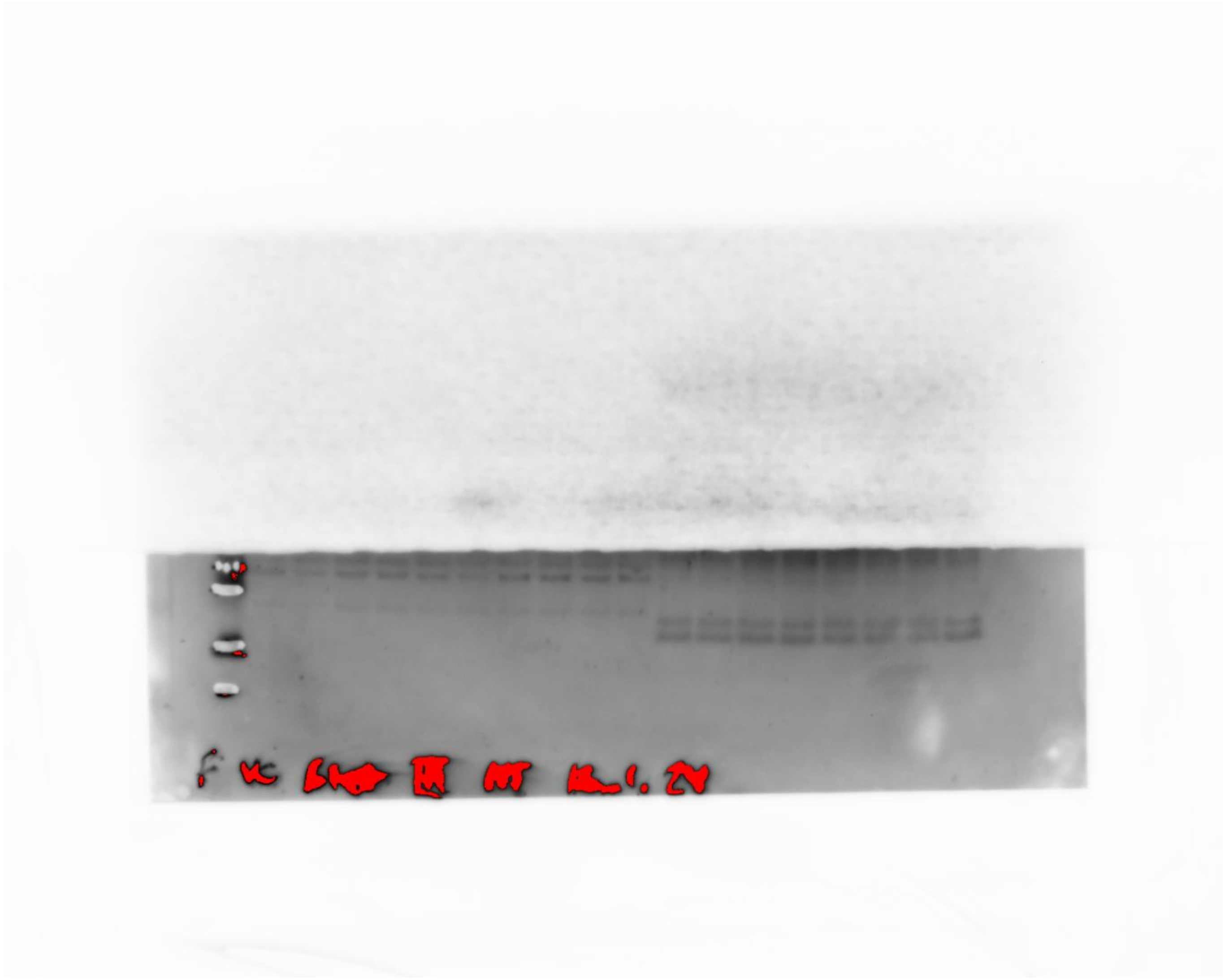

HC Blot I 6E10 1min

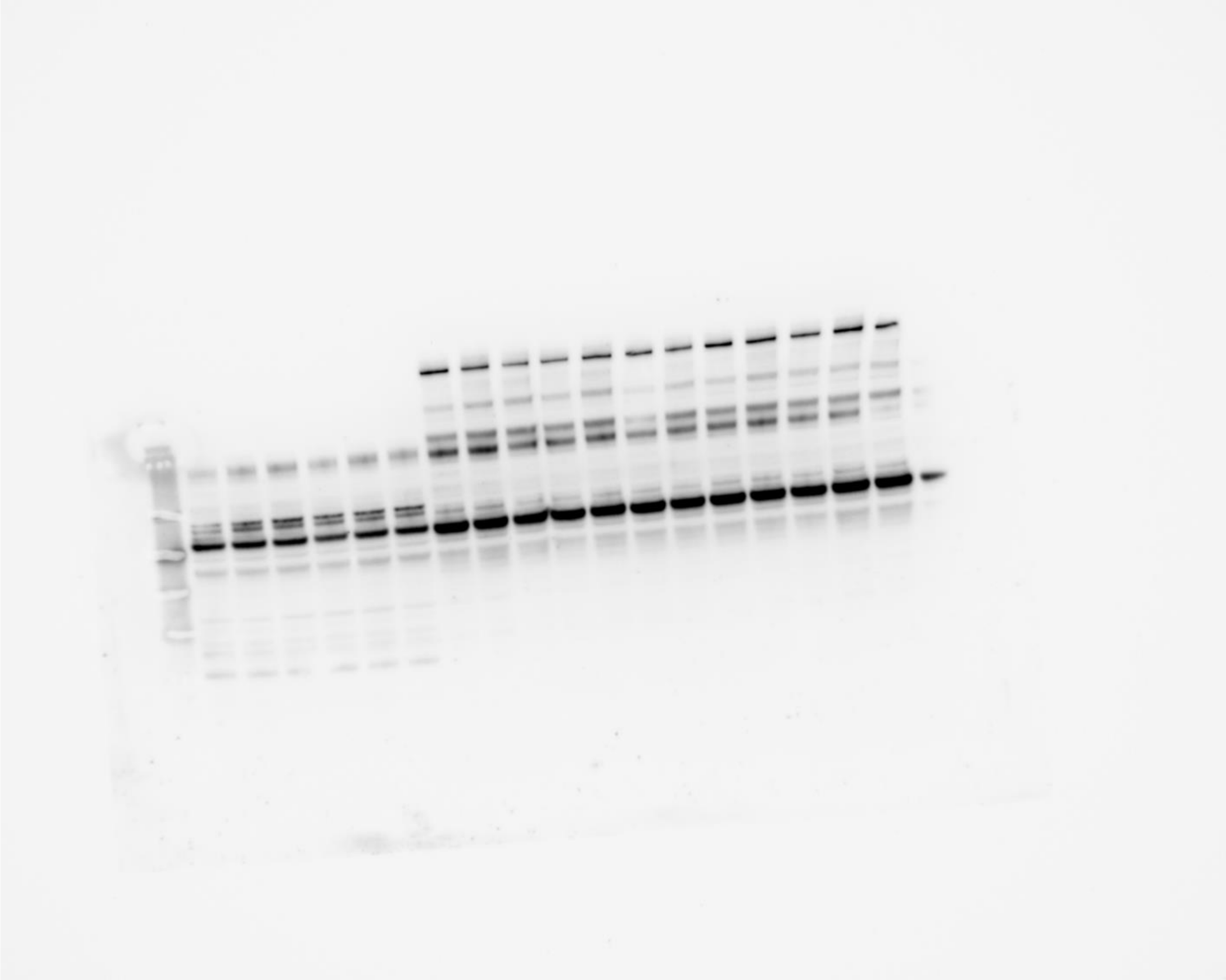

HC Blot I 6E10

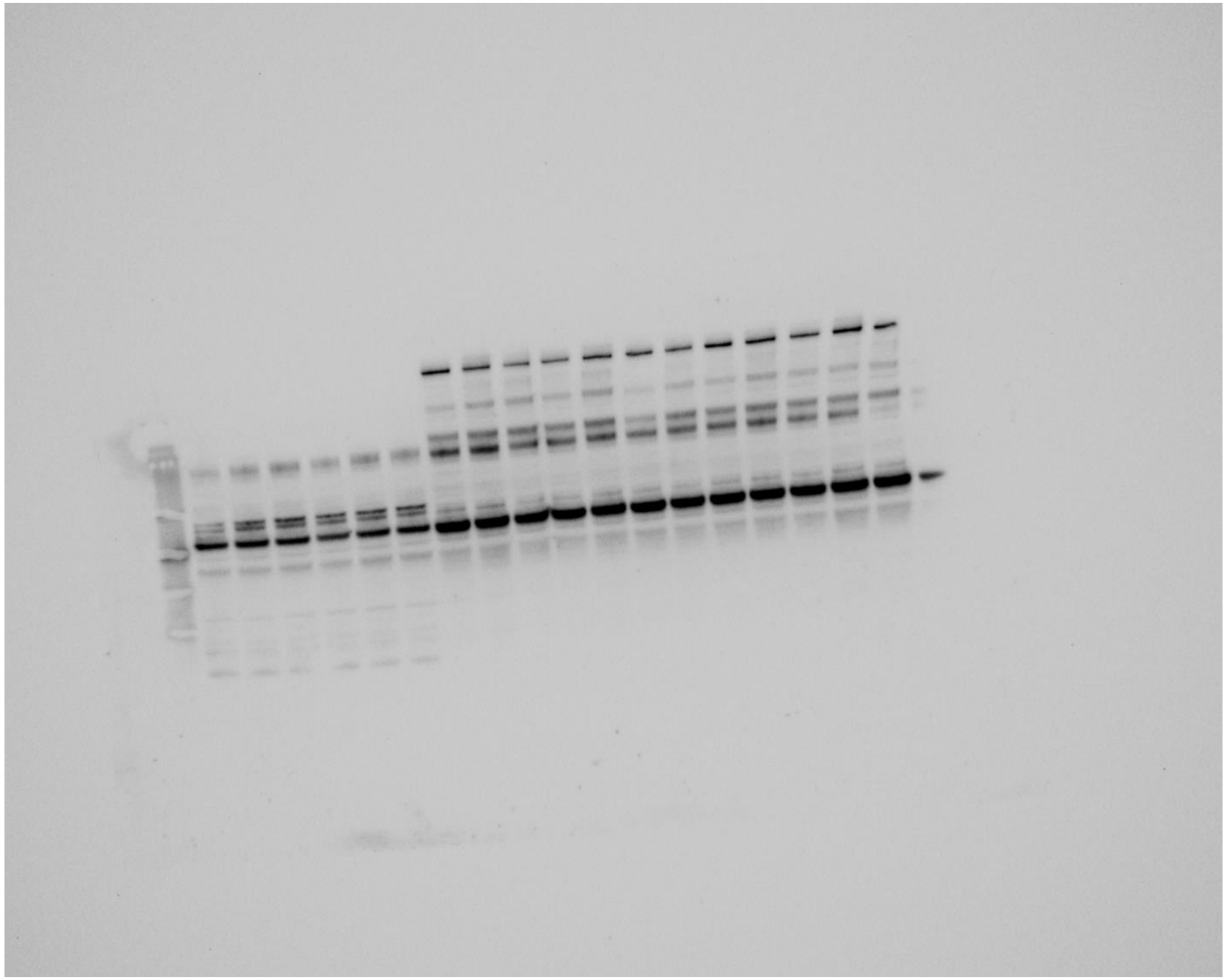

HC Blot I A8717 1min

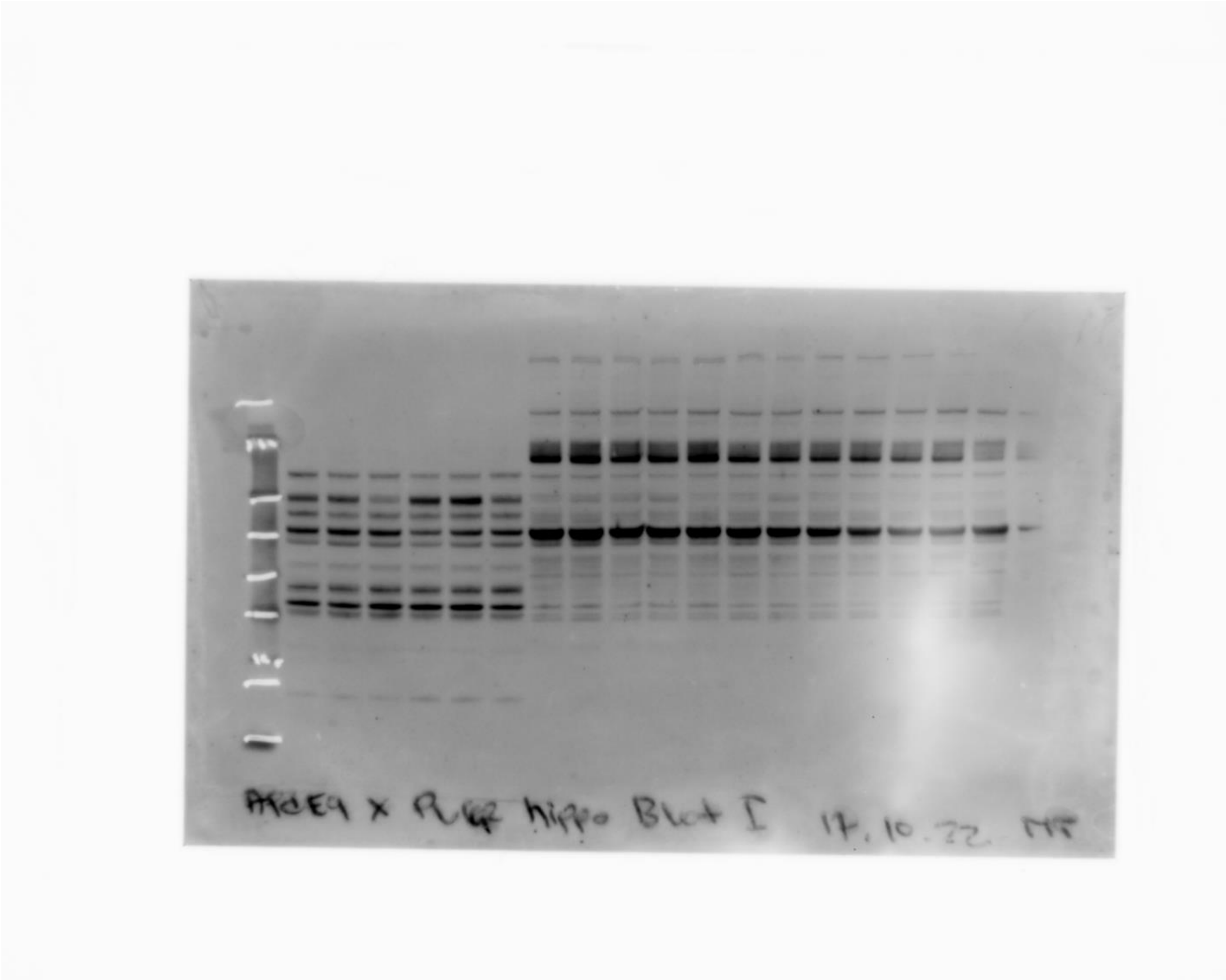

HC Blot I A8717

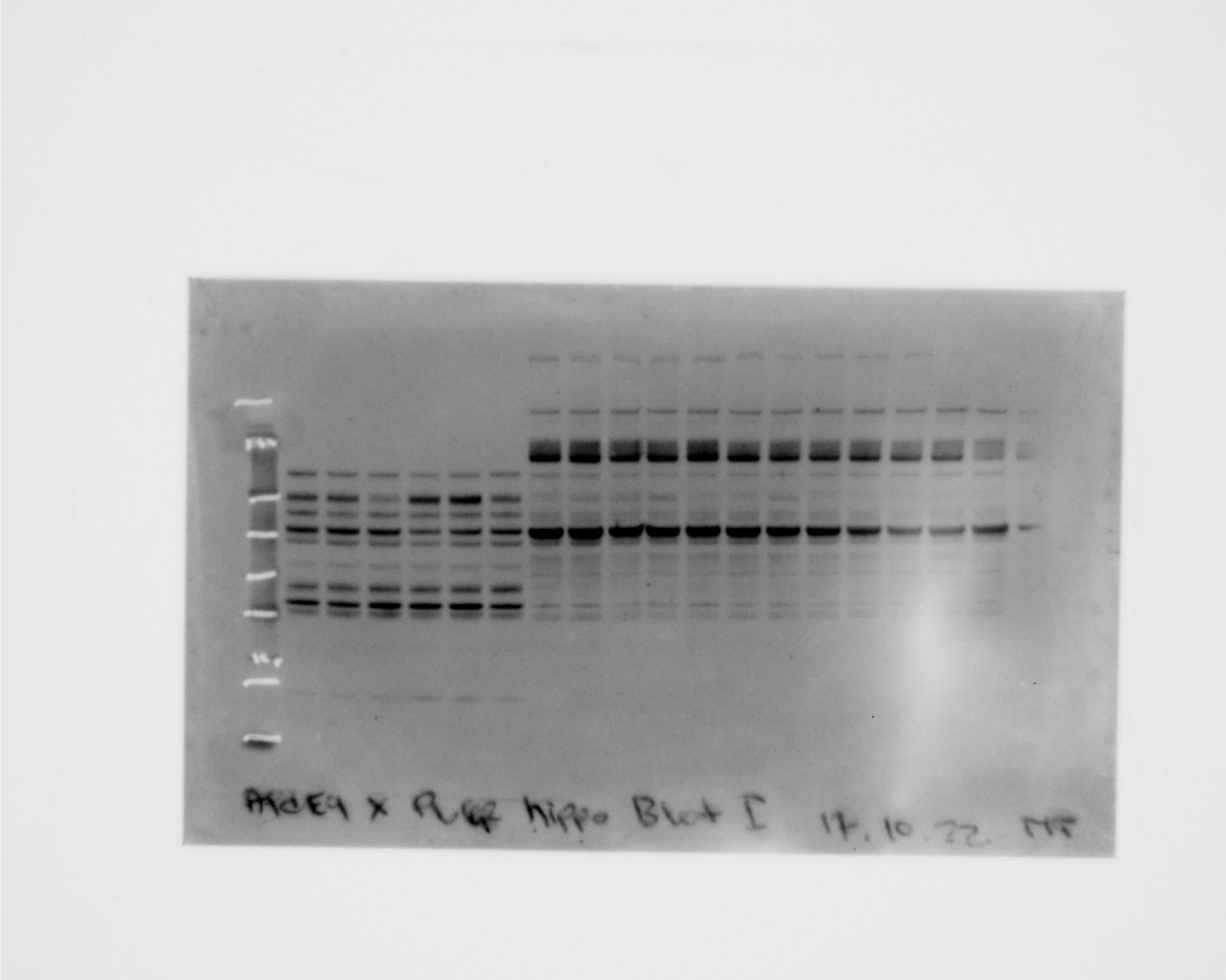

HC Blot I GAPDH

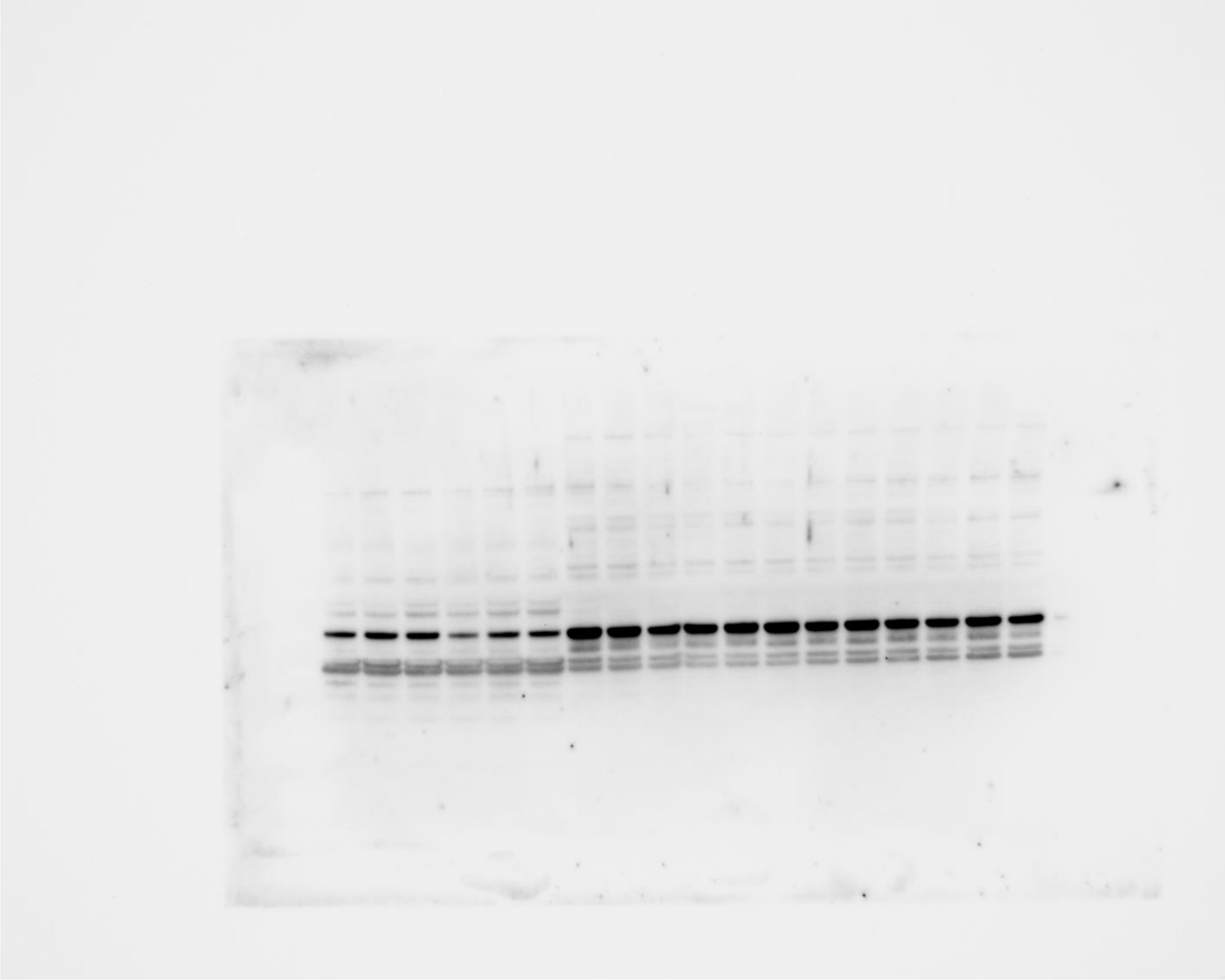

HC Blot II sAPP Beta

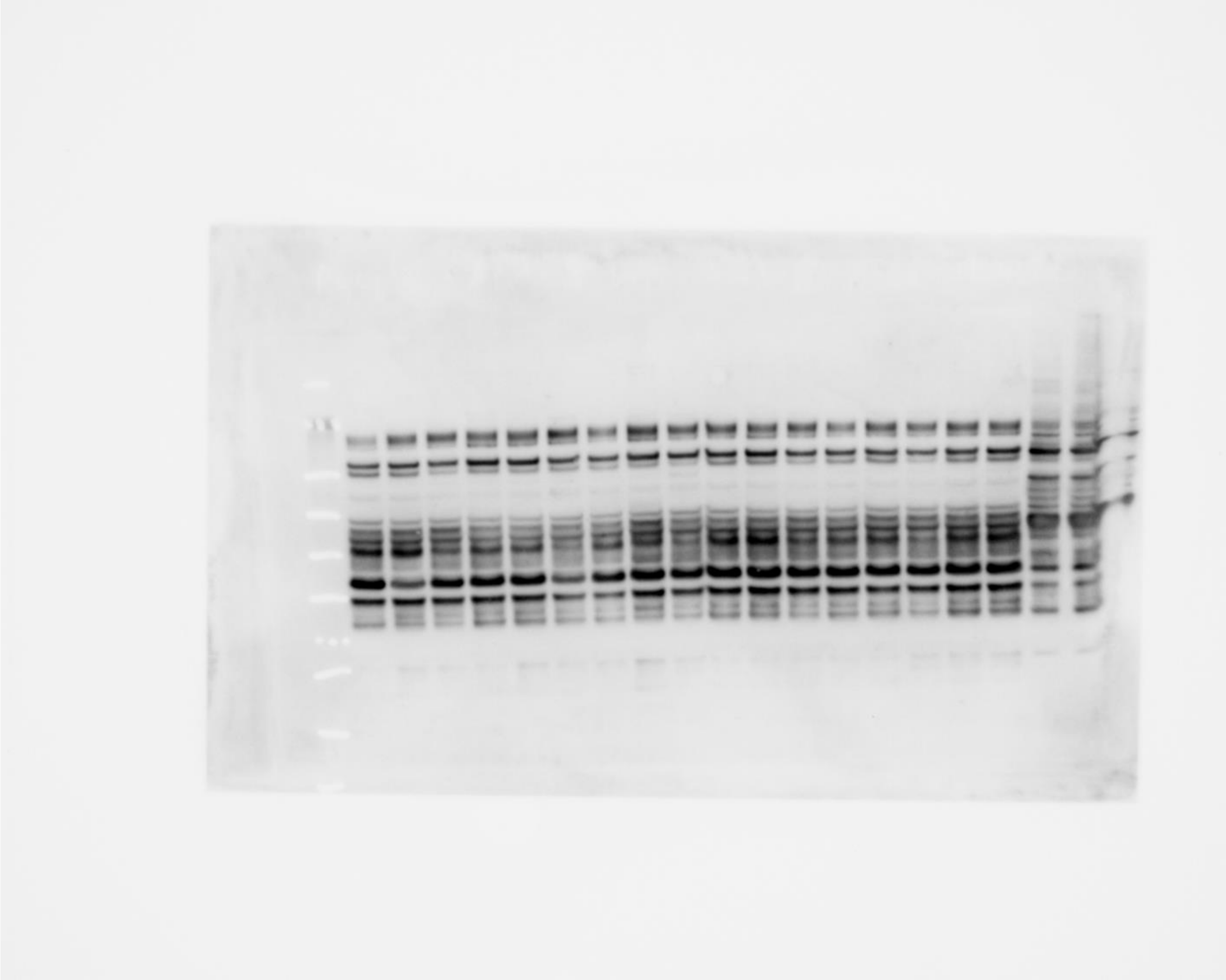

HC Blot III 22C11 30 sec

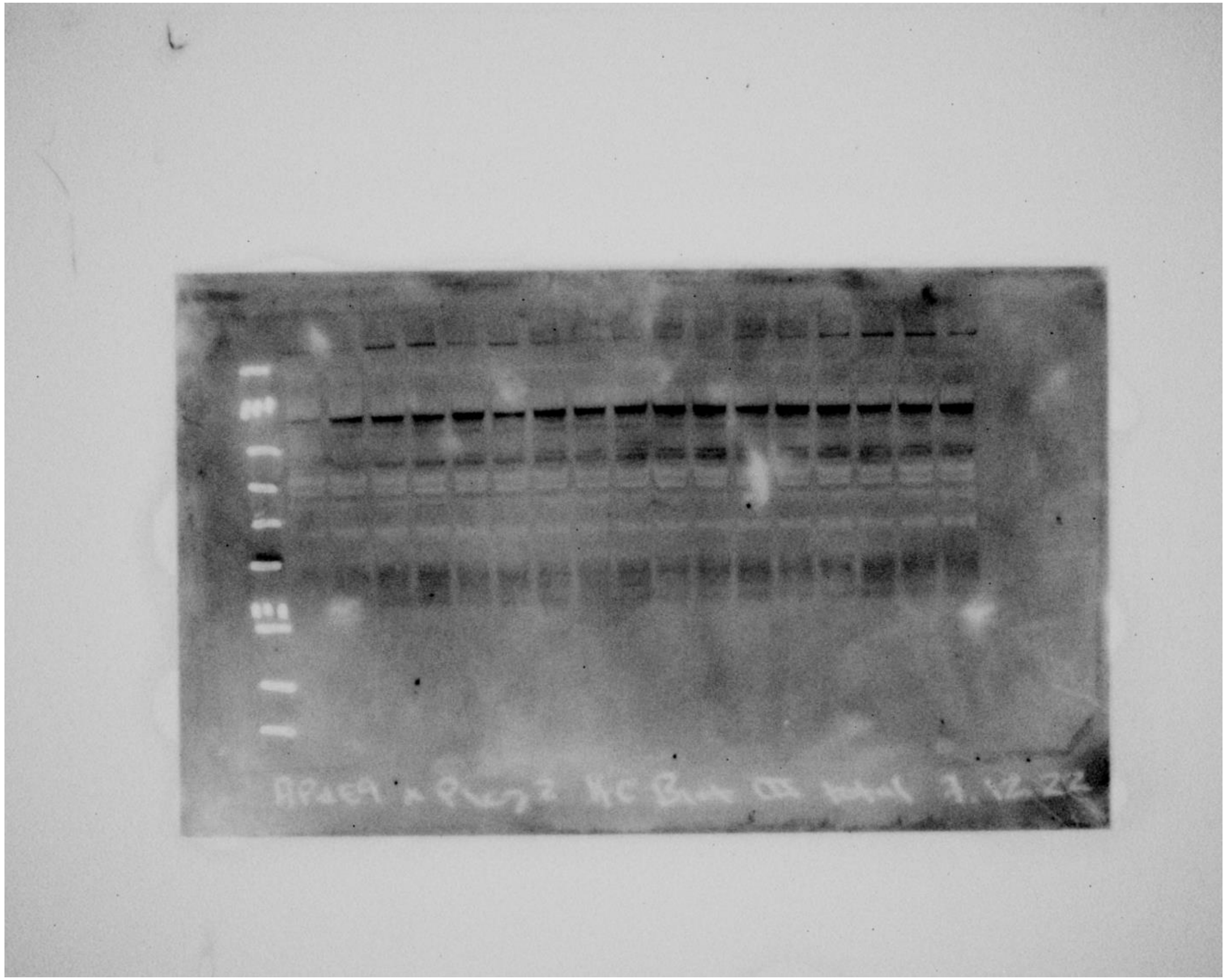

HC Blot III 22C11 45 sec

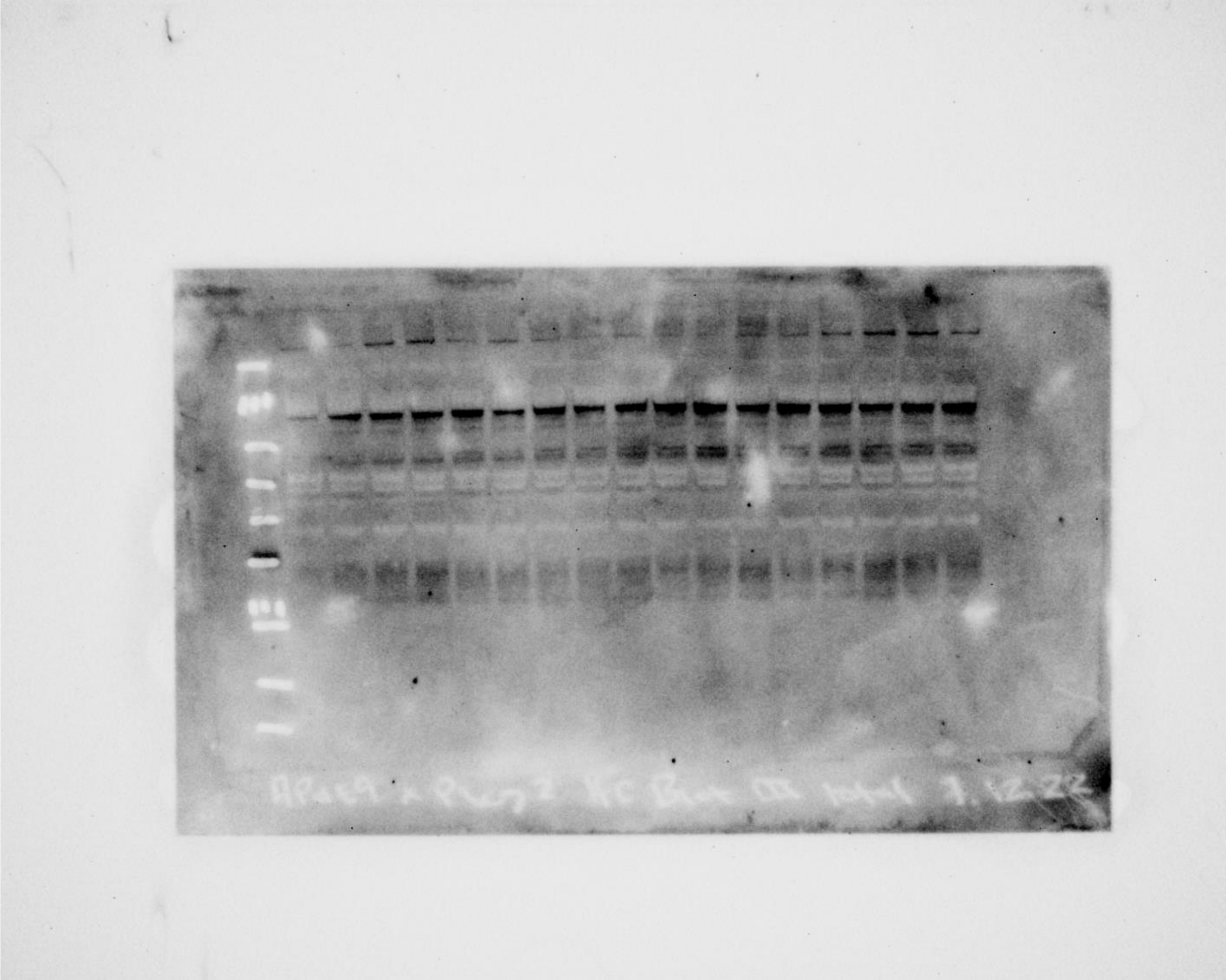

HC Blot III A8717 5s prime

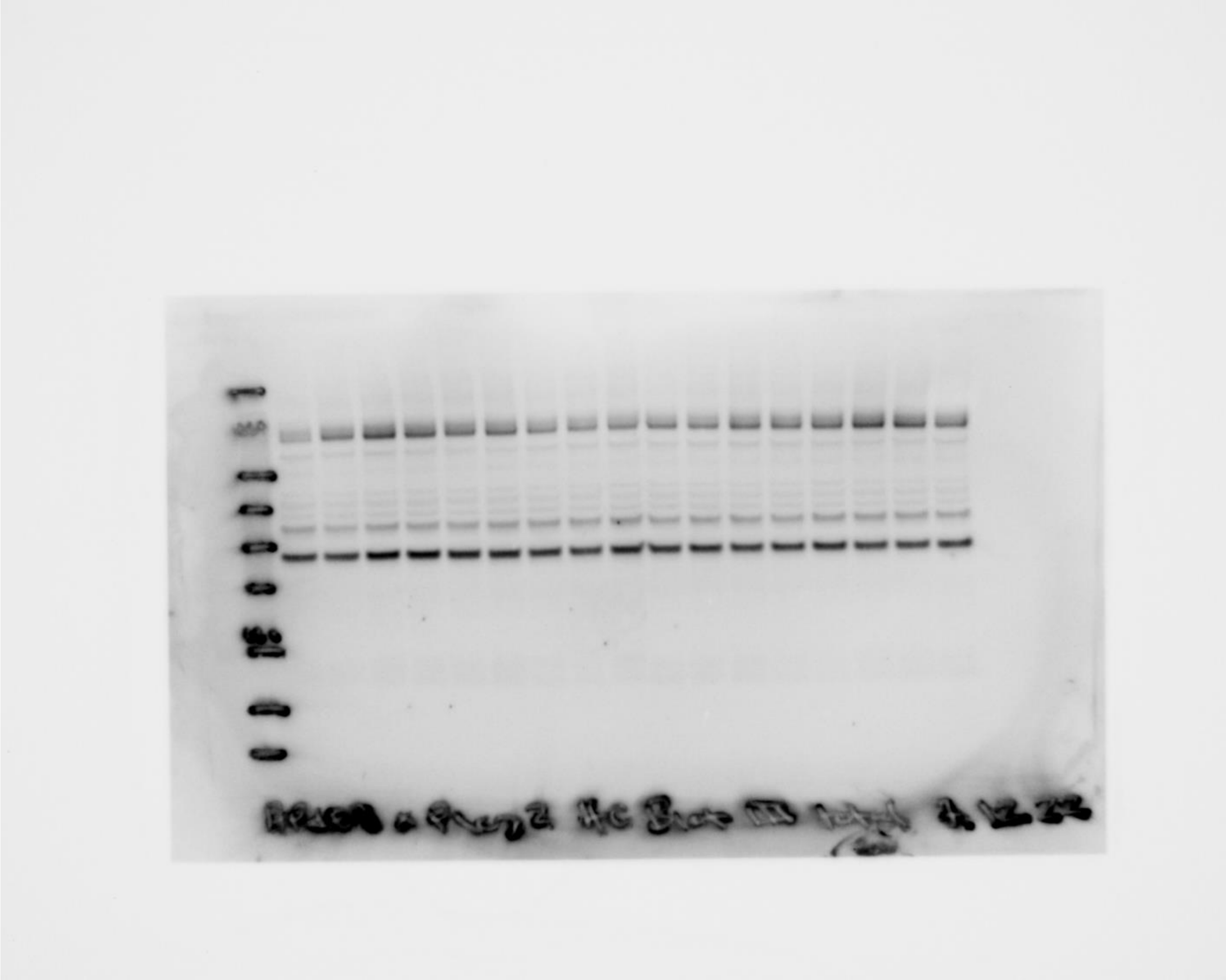

HC blot III A8717 CDF 30 sec select

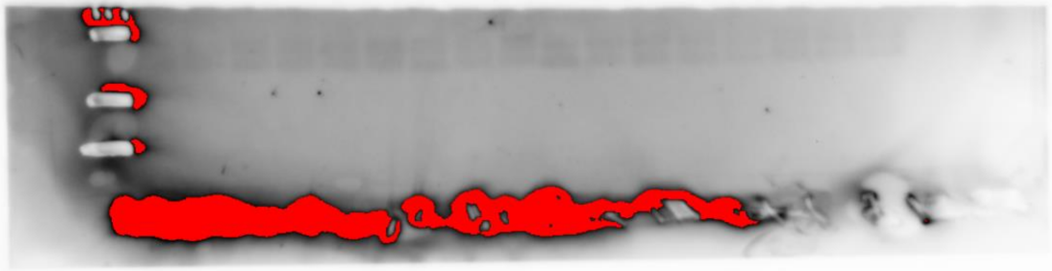

HC blot III A8717 CTF 60s prime

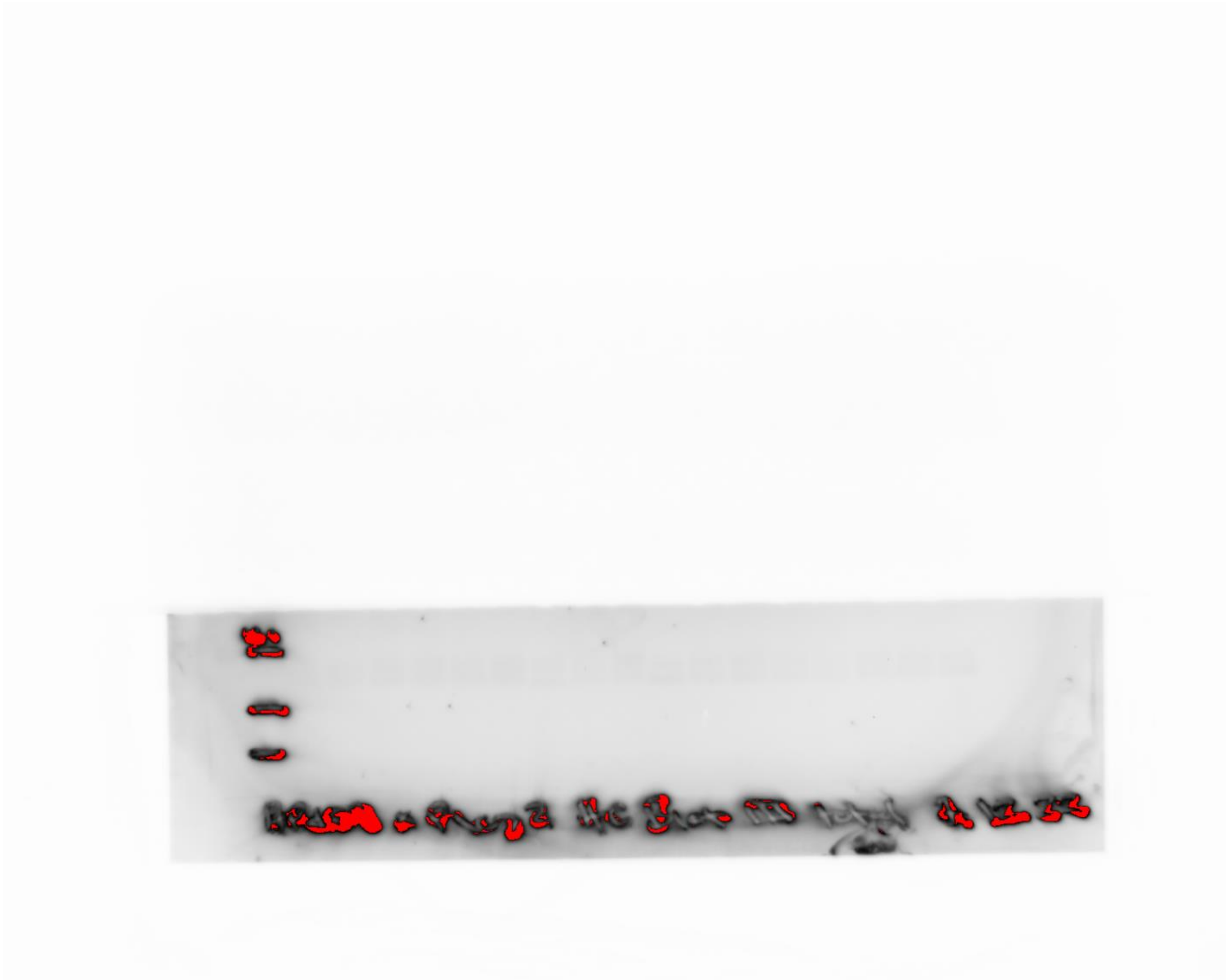

HC Blot III GAPDH ab8245 5 sec marker

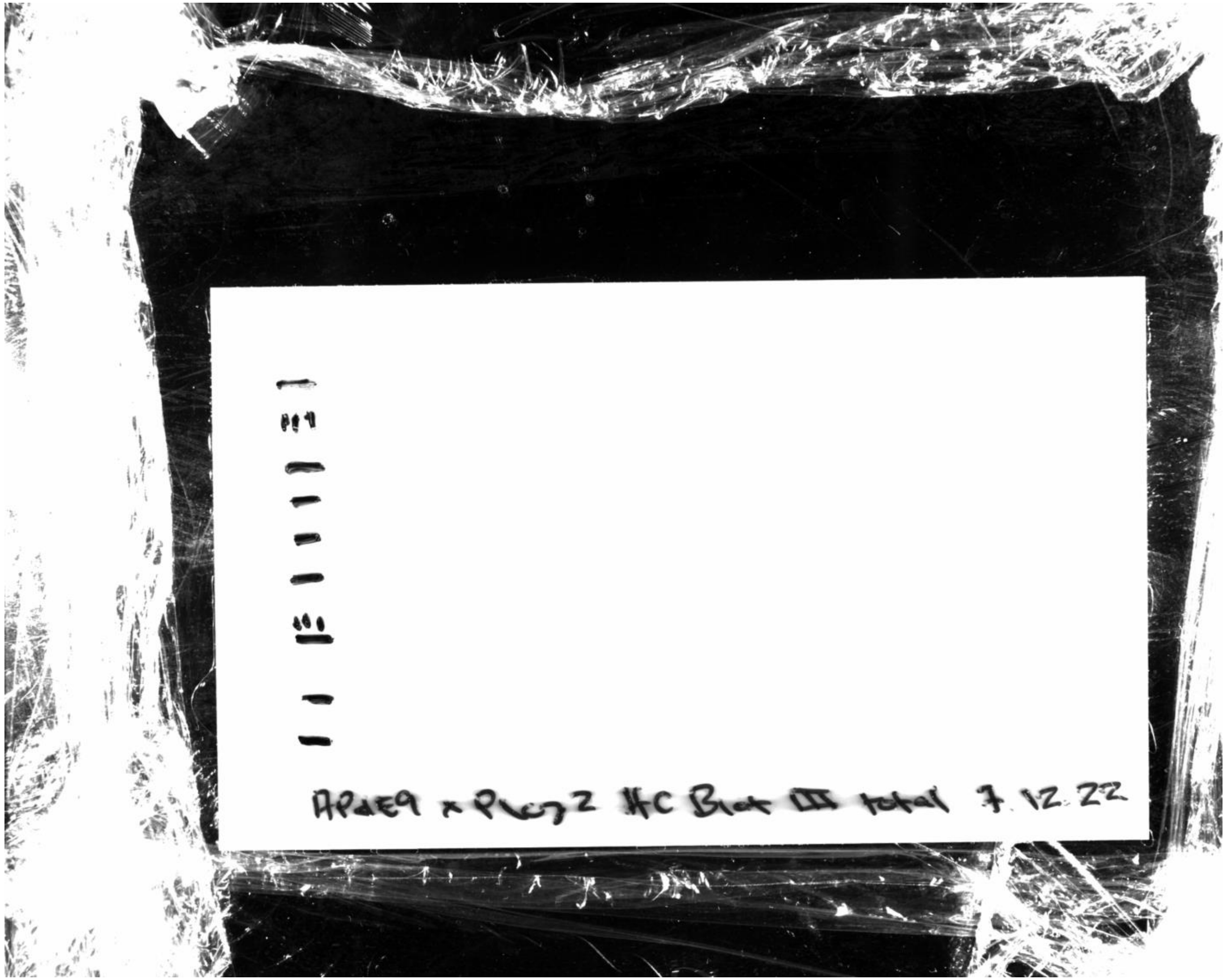

HC Blot III GAPDH ab8245 5 sec

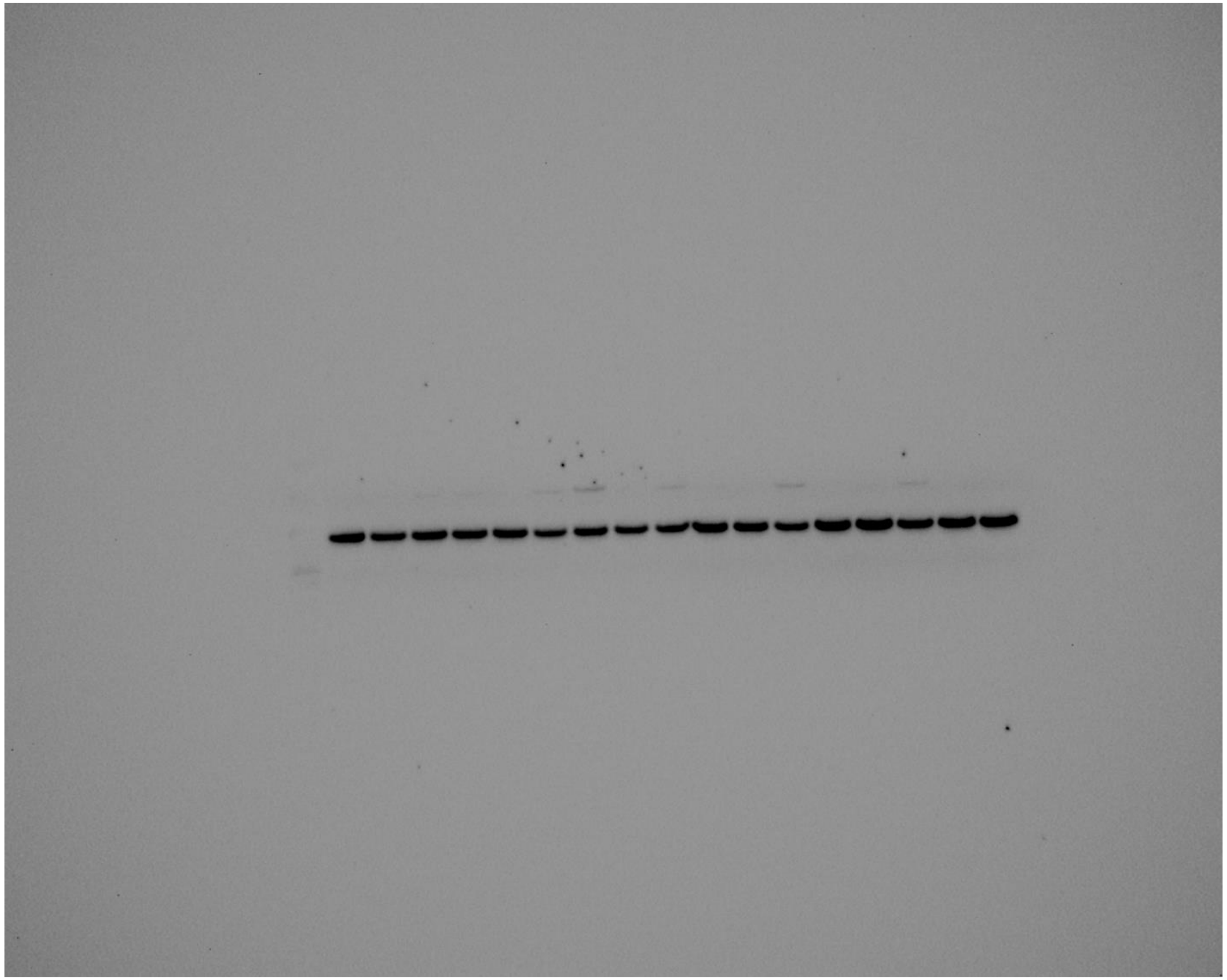

VC blot I sAPPbeta wild type

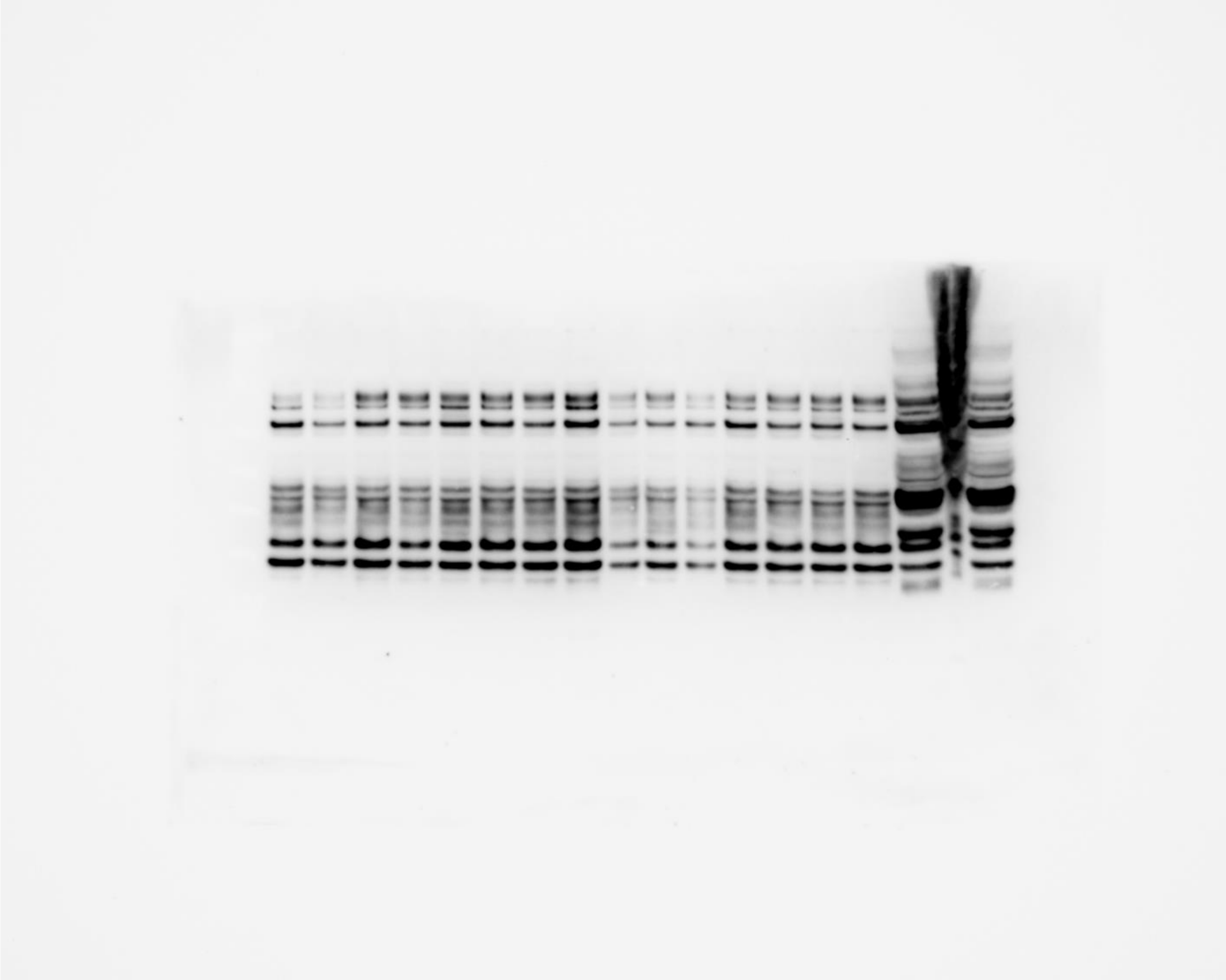

VC Blot II 22C11 30 sec

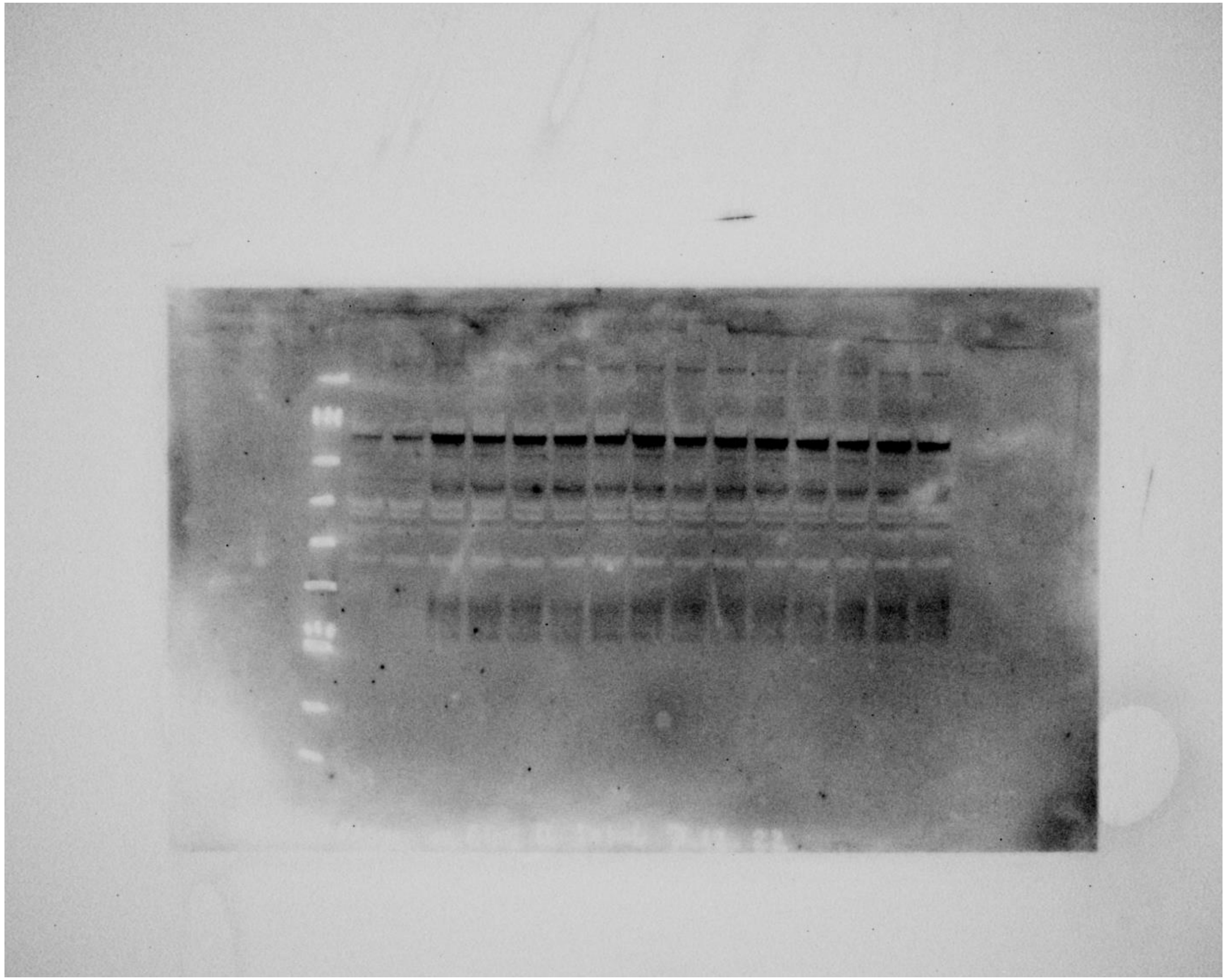

VC Blot II 22C11 45 sec

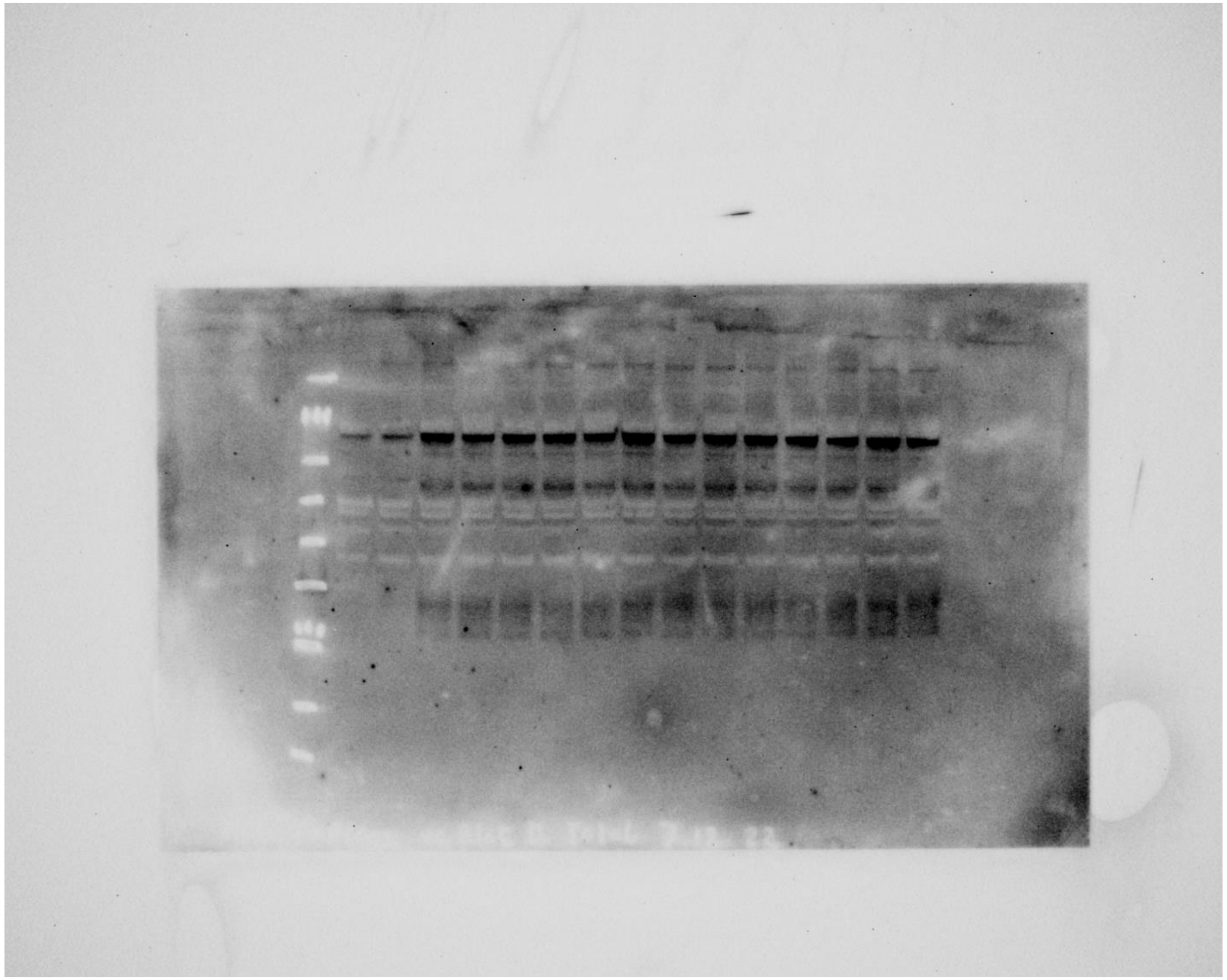

VC blot II A8717 CTF 1 min prime

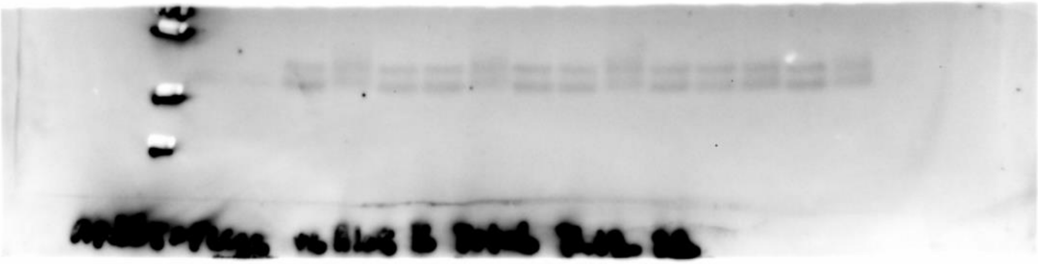

VC blot II A8717 CTF 30 s

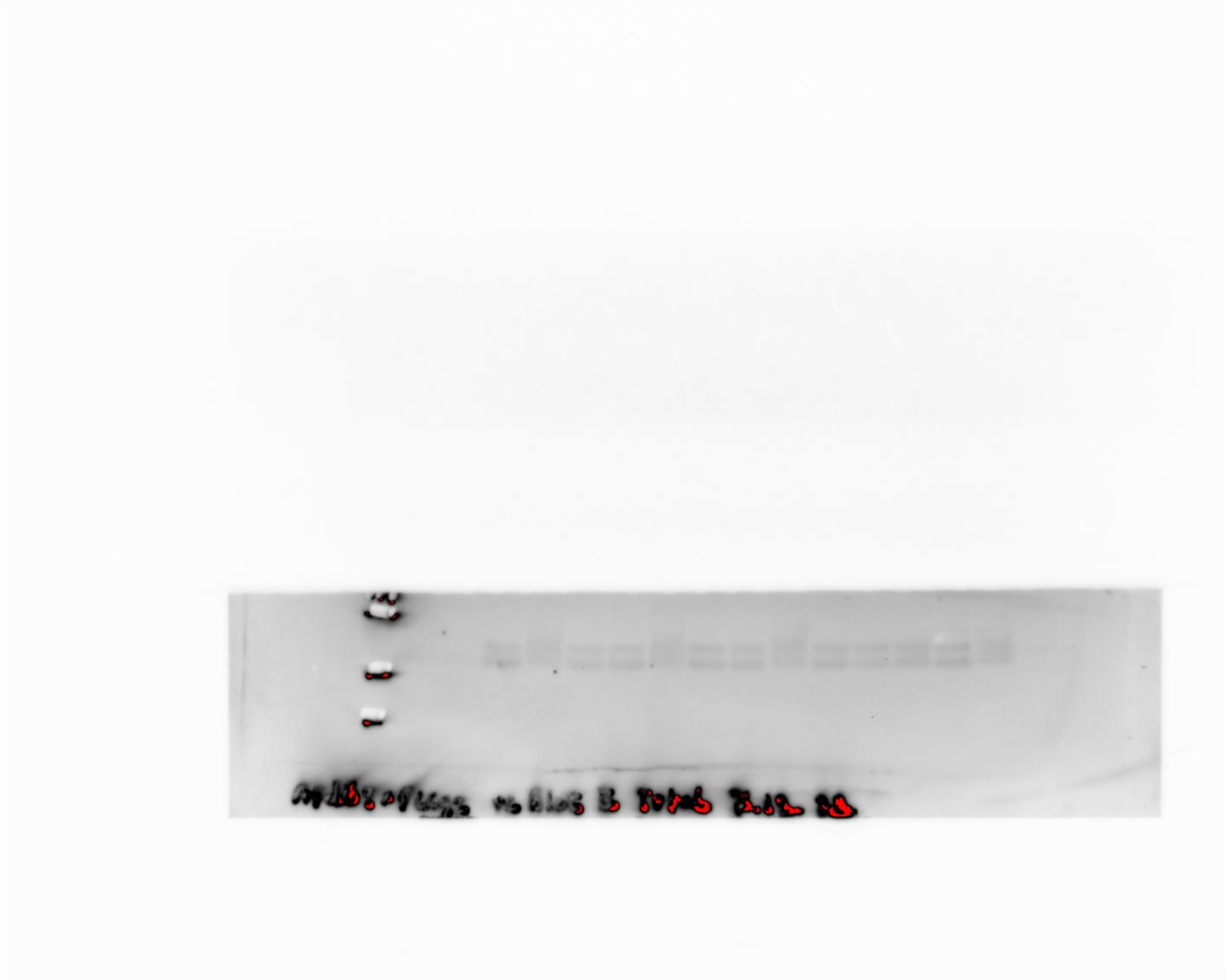

VC blot II A8718 5s prime

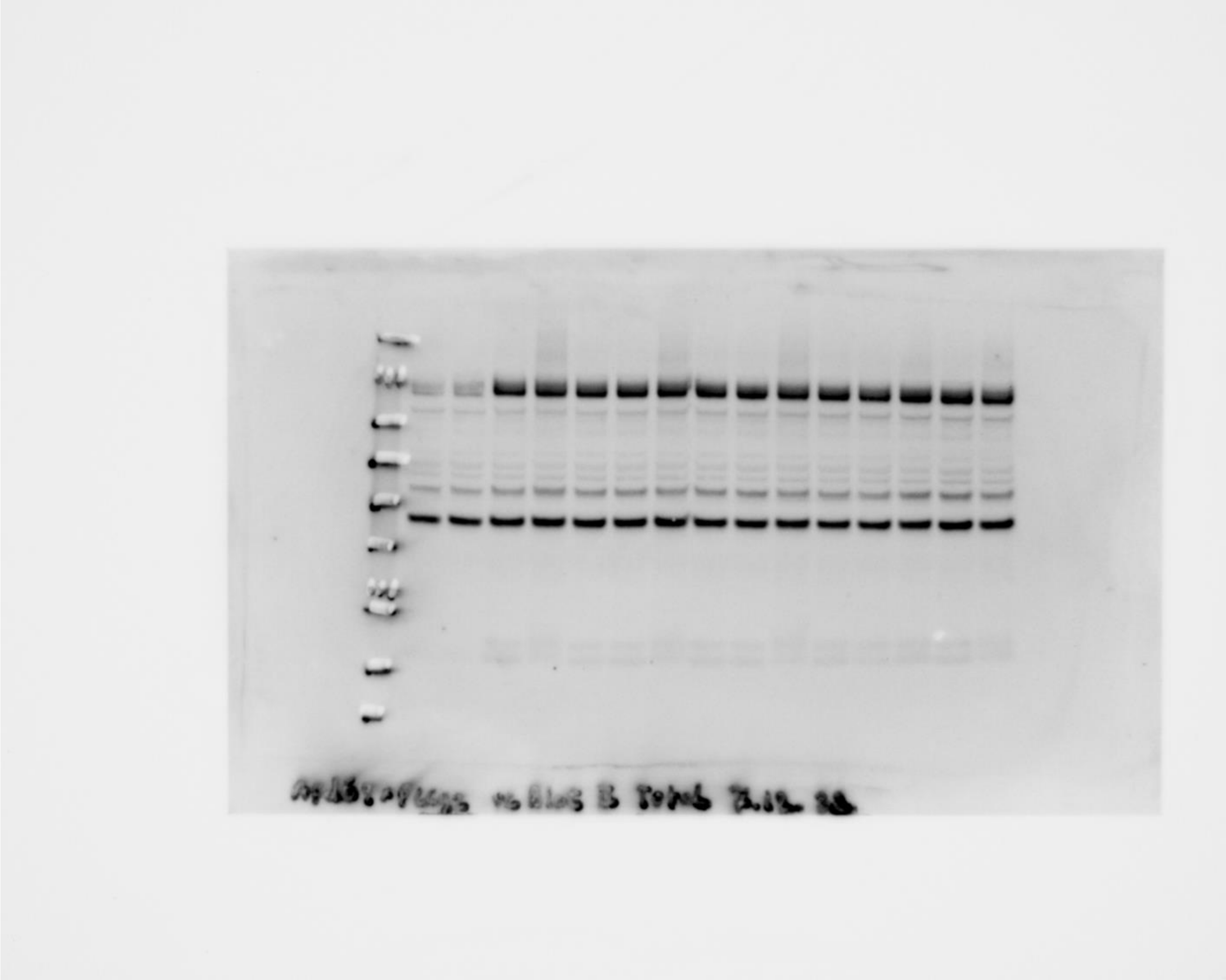

VC Blot II GAPDH ab8245 5 marker

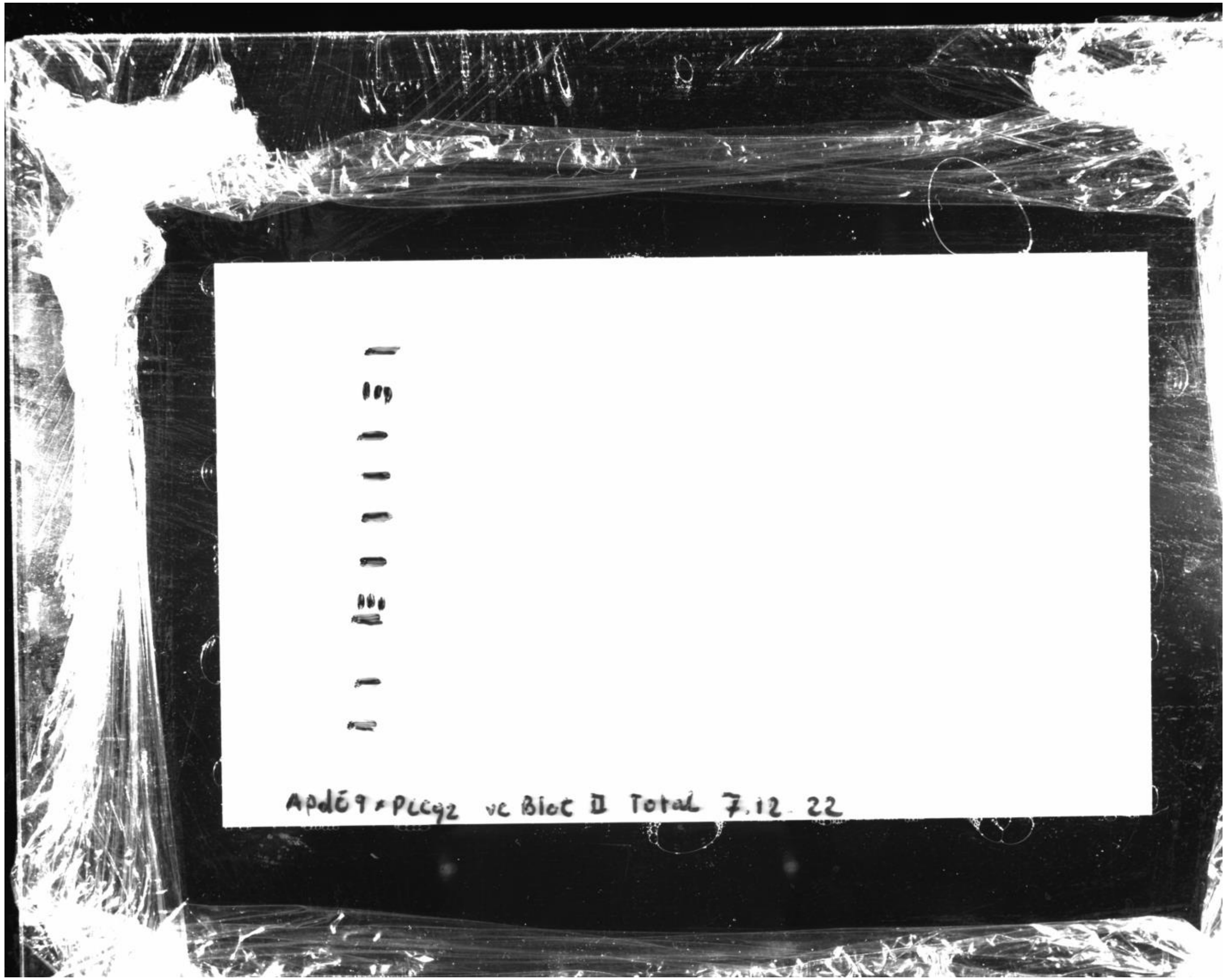

VC Blot II GAPDH ab8245 5 sec

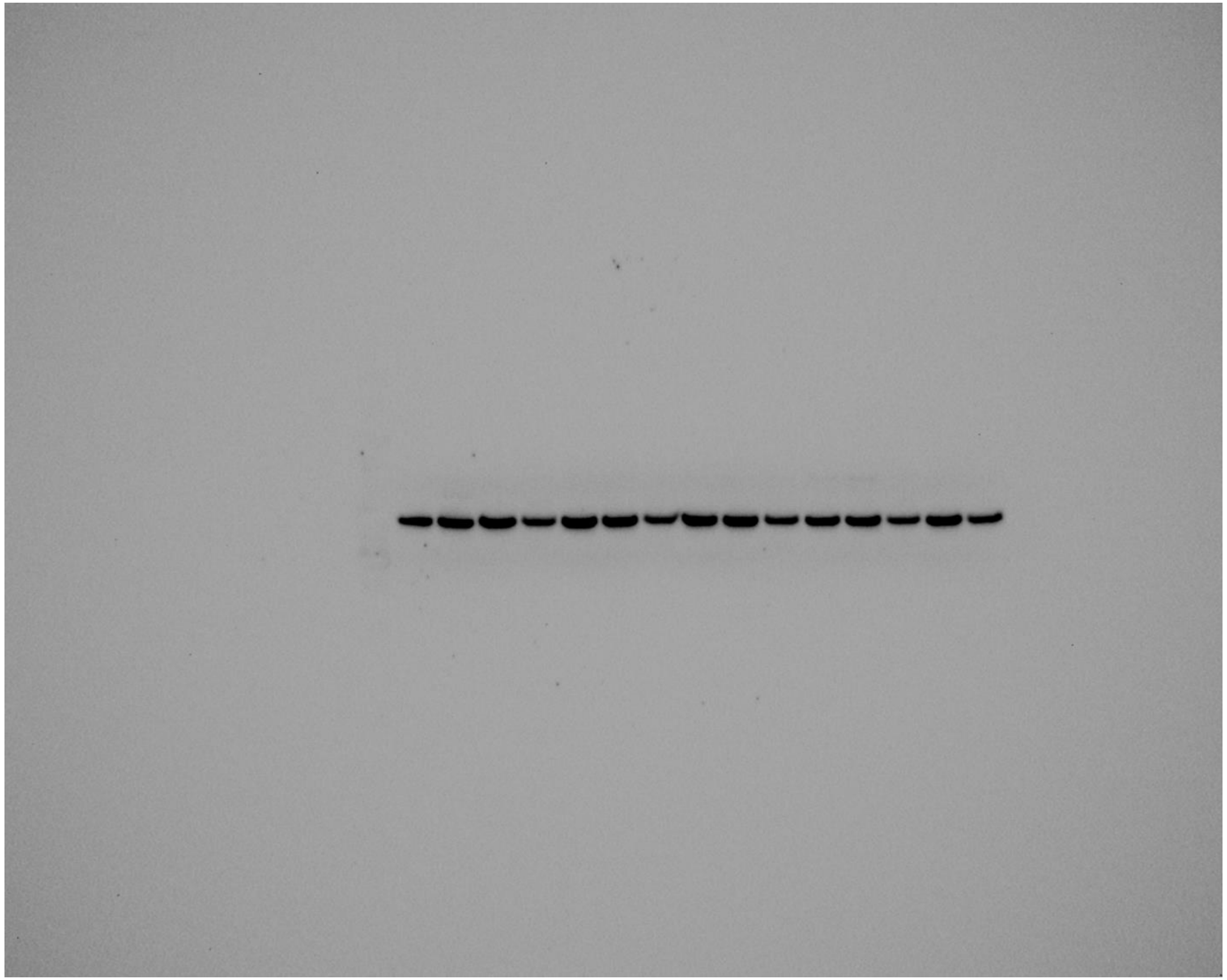

VC Blot V p-Tau (AT8) MN1020

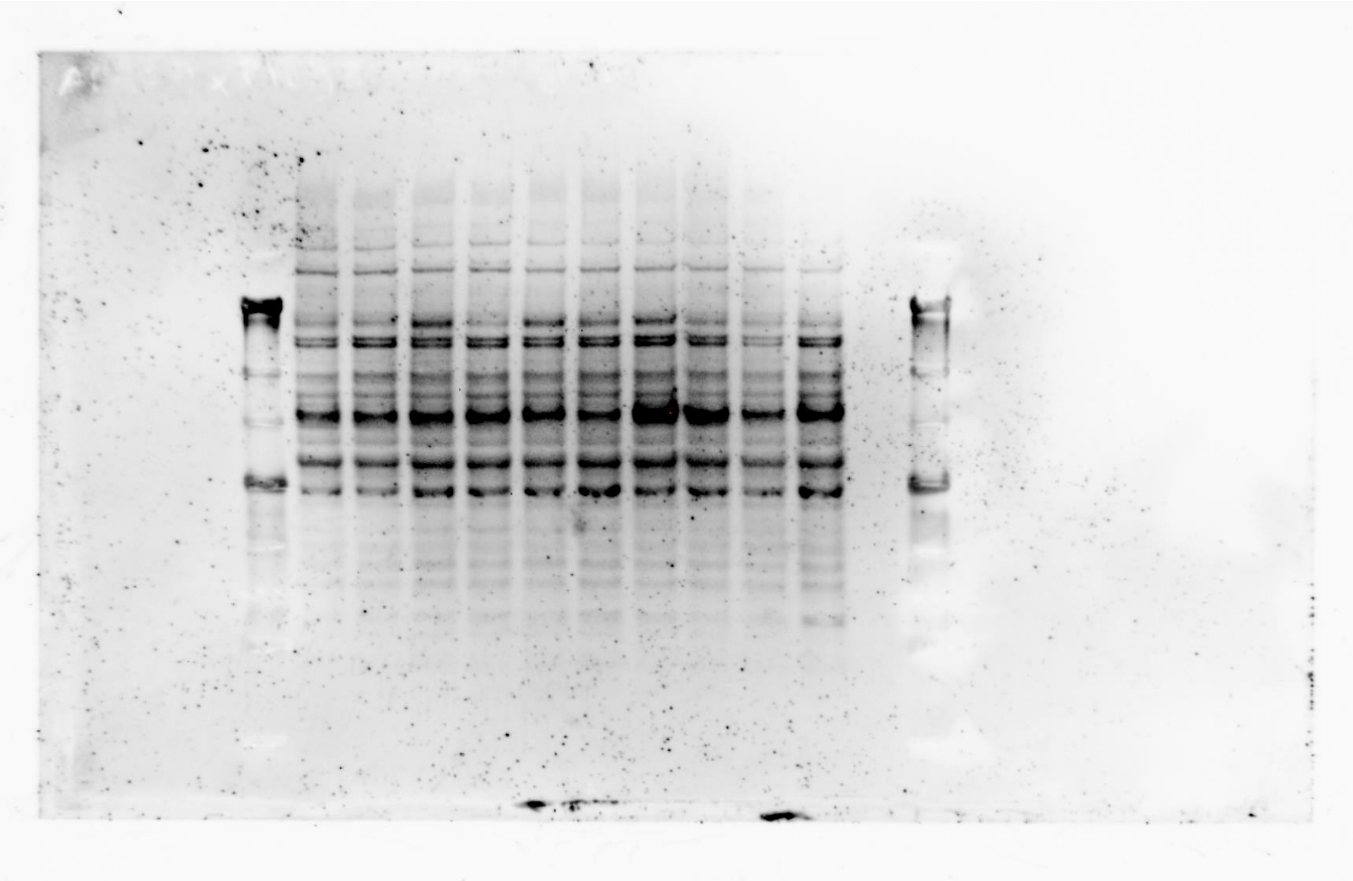

VC Blot V tau RD4 05-804

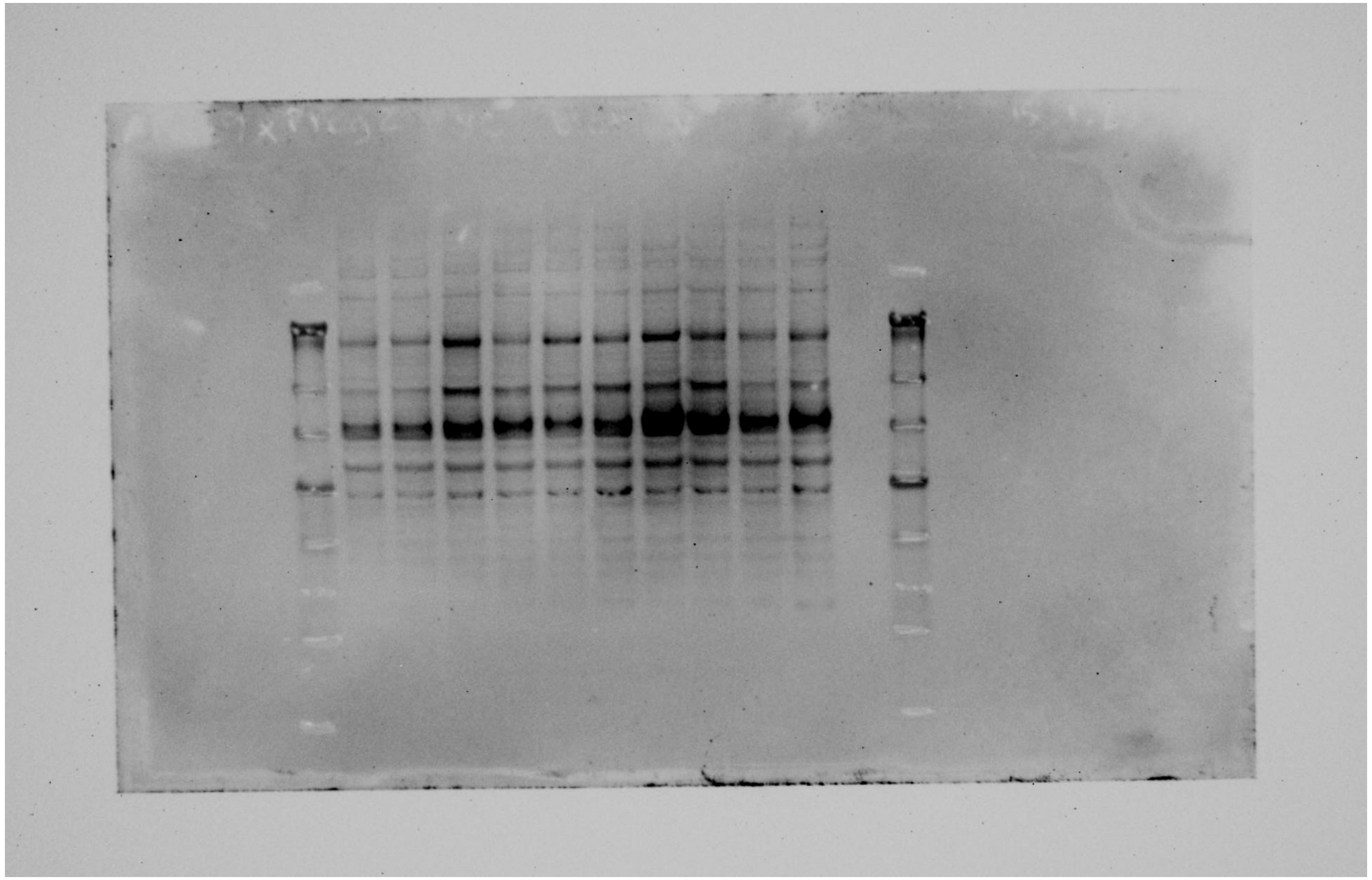

VC Blot V GAPDH ab8245

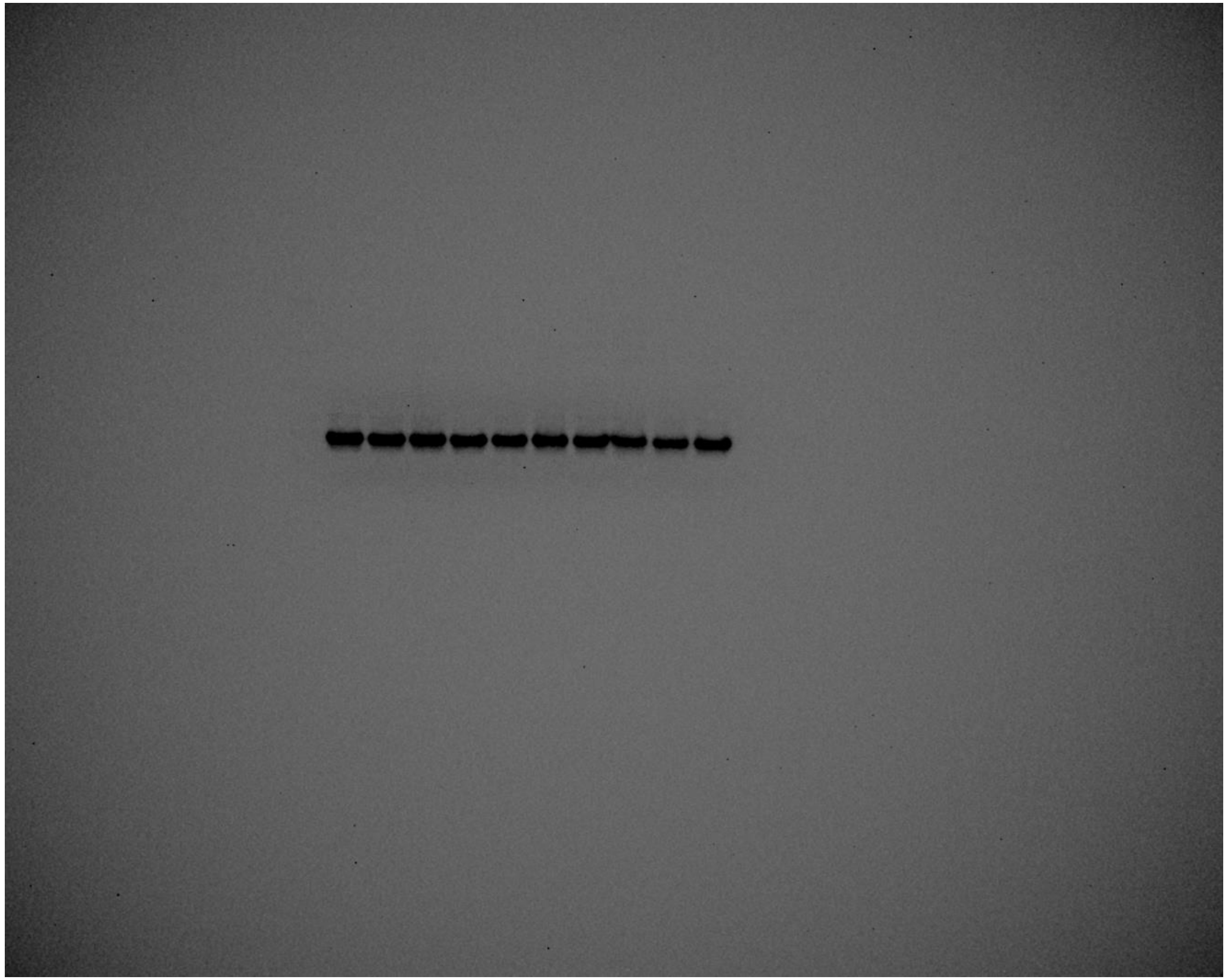

HC Blot VI p-Tau (AT8) MN1020

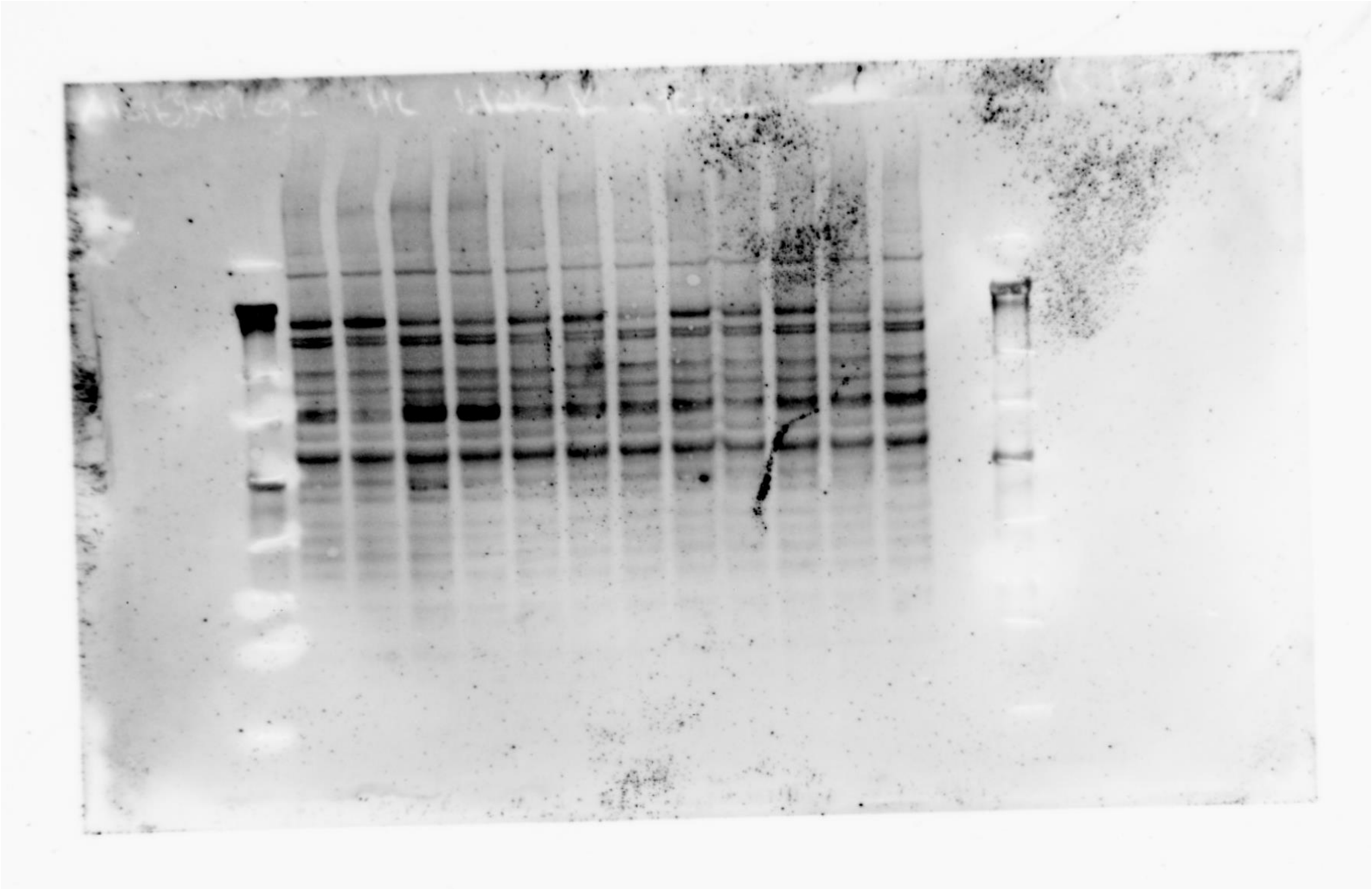

HC Blot VI Tau RD4 05-804

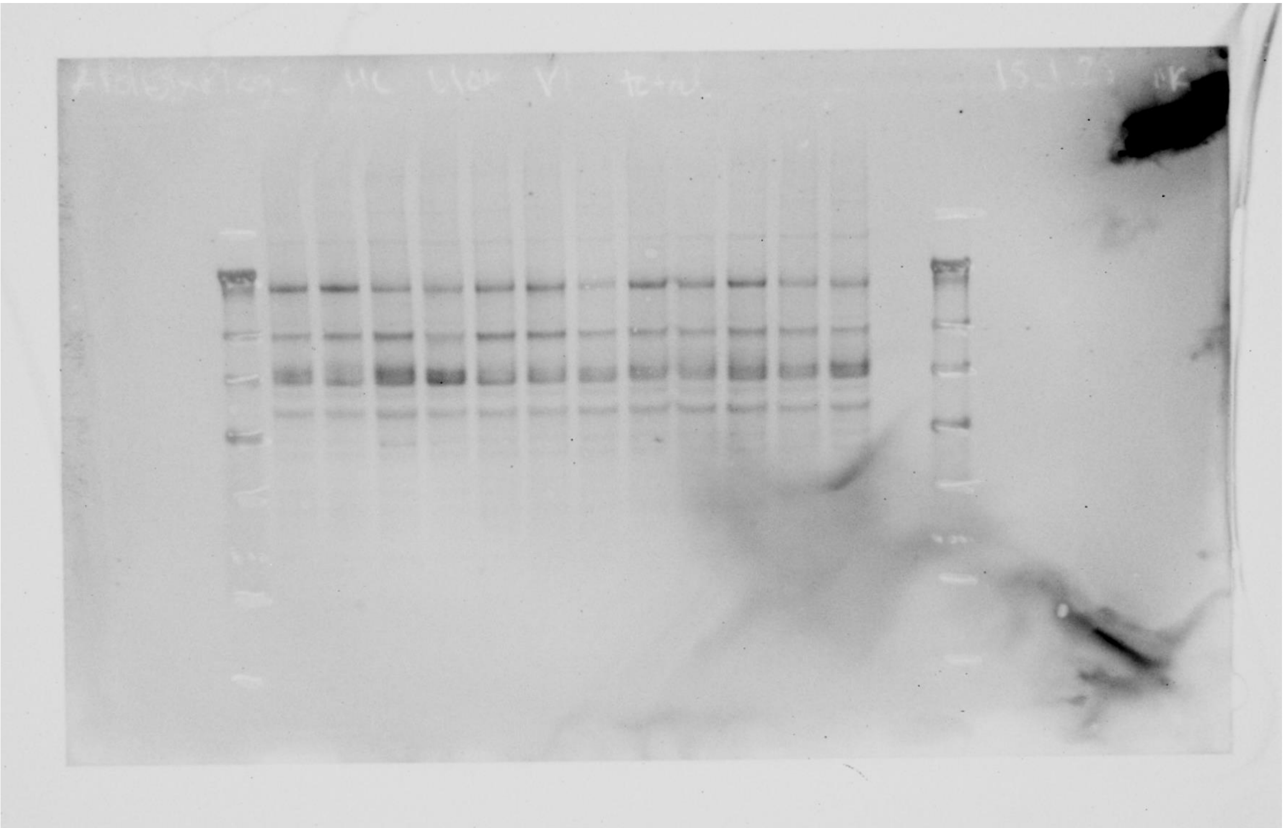

HC Blot VI GAPDH ab8245

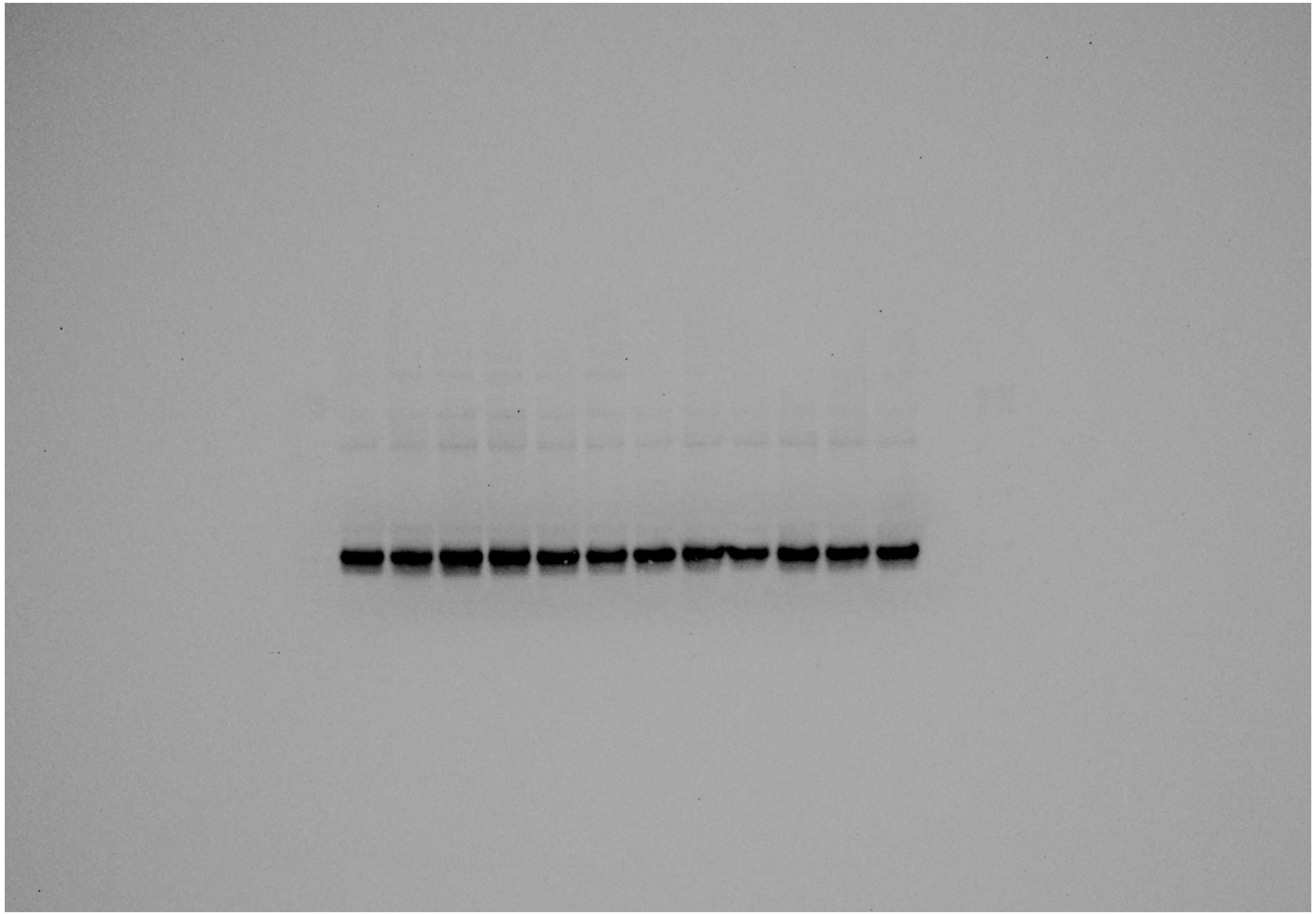

Supplement: Supplementary file 1 — Additional file 1 [file 12974_2025_3387_MOESM1_ESM.pdf]
