# Supplementary material for: The protective PLCγ2-P522R variant mitigates Alzheimer’s disease-associated pathologies by enhancing beneficial microglial functions
Source: J Neuroinflammation. 2025 Mar 5;22:64. doi: 10.1186/s12974-025-03387-6 (PMC11881468; doi:10.1186/s12974-025-03387-6)
Supplement: Supplementary file 3 — Additional file 3 [file 12974_2025_3387_MOESM3_ESM.pdf]

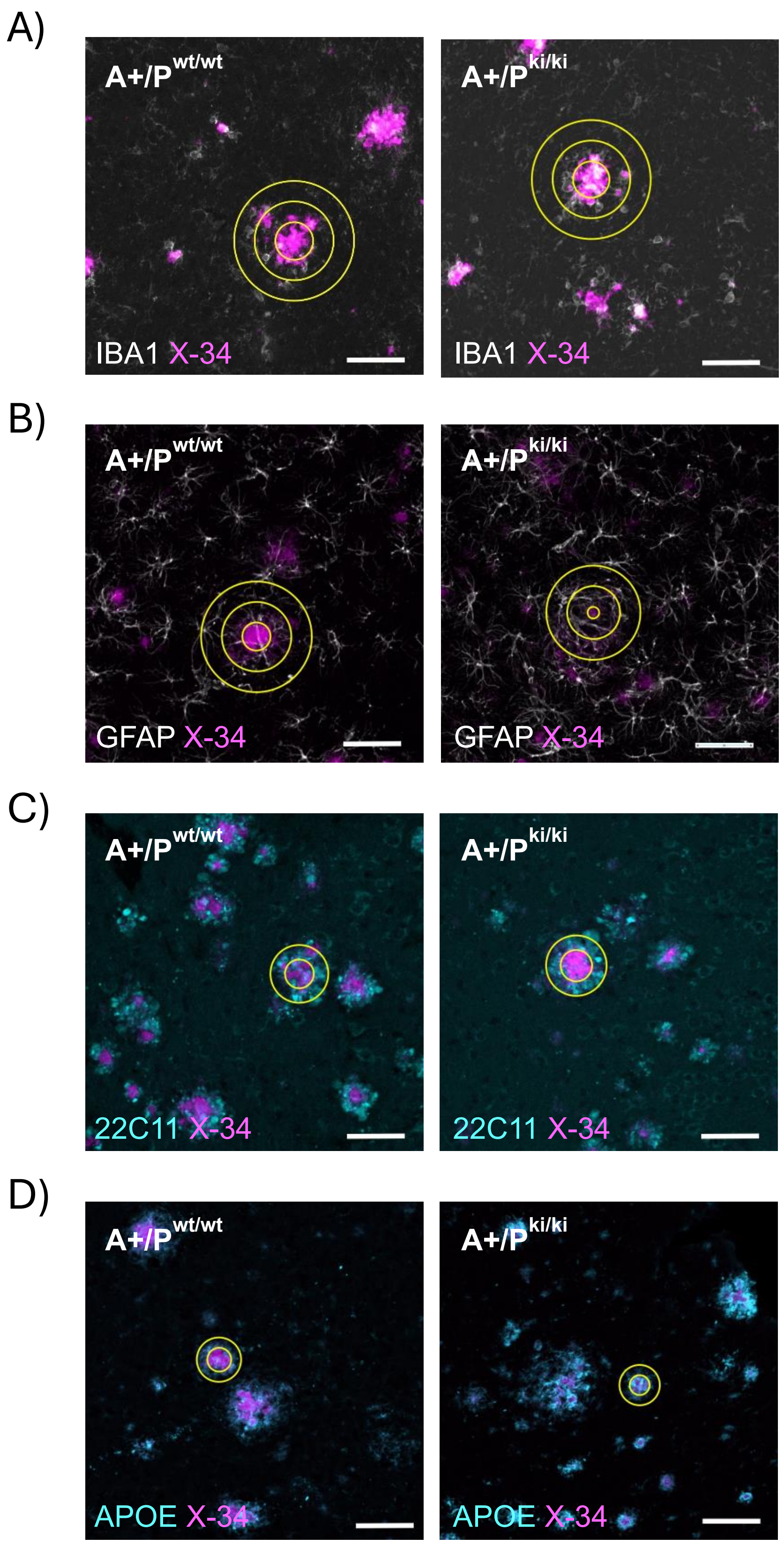
A)
A+/P wt/wt
IBA1 X-34
A+/P ki/ki
IBA1 X-34
B)
A+/P wt/wt
GFAP X-34
A+/P ki/ki
GFAP X-34
C)
A+/P wt/wt
22C11 X-34
A+/P ki/ki
22C11 X-34
D)
A+/P wt/wt
APOE X-34
A+/P ki/ki
APOE X-34

Supplementary Figure 1. Representative ROIs used for analyzing IBA1, GFAP, 22C11, and APOE within and surrounding β-amyloid plaques. The inner circle was calculated based on the plaque size and width of the outer circle(s) were kept constant within each analysis. Analysis of IBA1 and GFAP within 0-20 and 20-40 μm (A-B), 22C11 within 0-14 μm (C), and APOE within 0-20 μm (D) from the plaque outline was conducted.

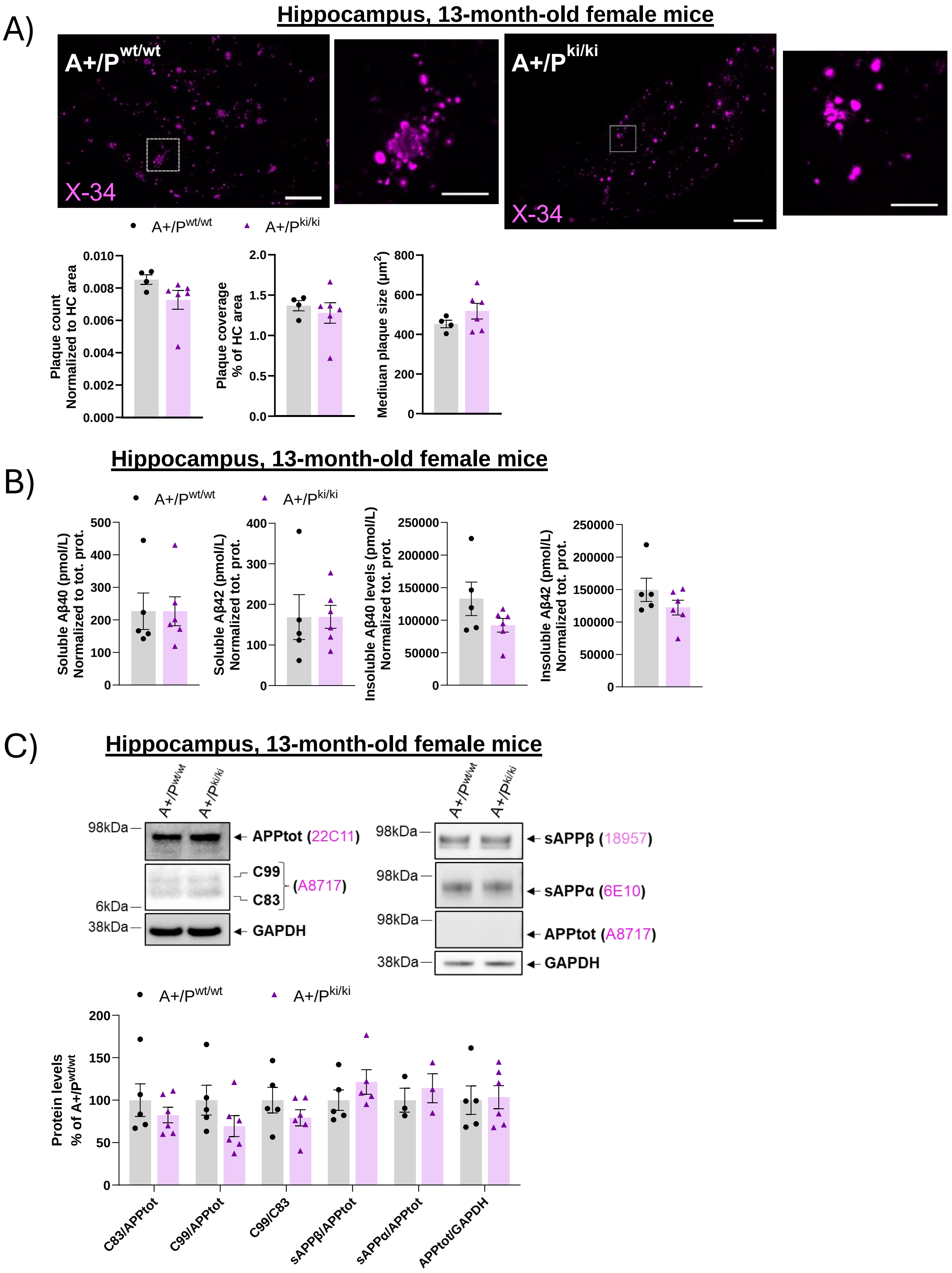

A)
Hippocampus, 13-month-old female mice
A+/Pwt/wt
X-34
A+/Pki/ki
X-34
A+/Pwt/wt
A+/Pki/ki
Plaque count Normalized to HC area
0.010
0.008
0.006
0.004
0.002
0.000
Plaque coverage % of HC area
2.0
1.5
1.0
0.5
0.0
Mediuan plaque size (µm²)
800
600
400
200
0
B)
Hippocampus, 13-month-old female mice
A+/Pwt/wt
A+/Pki/ki
Soluble Aβ40 (pmol/L) Normalized to tot. prot.
500
400
300
200
100
0
Soluble Aβ42 (pmol/L) Normalized tot. prot.
400
300
200
100
0
Insoluble Aβ40 levels (pmol/L) Normalized tot. prot.
250000
200000
150000
100000
50000
0
Insoluble Aβ42 (pmol/L) Normalized tot. prot.
250000
200000
150000
100000
50000
0
C)
Hippocampus, 13-month-old female mice
A+/Pwt/wt
A+/Pki/ki
98kDa
APPtot (22C11)
C99
C83
(A8717)
6kDa
38kDa
GAPDH
98kDa
sAPPβ (18957)
98kDa
sAPPα (6E10)
98kDa
APPtot (A8717)
38kDa
GAPDH
A+/Pwt/wt
A+/Pki/ki
Protein levels % of A+/Pwt/wt
200
150
100
50
0
C83/APPtot
C99/APPtot
C99/C83
sAPPβ/APPtot
sAPPα/APPtot
APPtot/GAPDH

Supplementary Figure 2. PLCγ2-P522R variant does not change β-amyloid load in the hippocampus of female APP/PS1 mice. A) β-amyloid plaque count, coverage (total plaque area, % of whole analyzed area, μm^2), and size (μm^2) of the individual plaques in the hippocampus of the APP/PS1xPlγ2-P522R (A+/$P^{ki/ki}$) and APP/PS1 (A+/$P^{wt/wt}$) mice. n(A+/$P^{wt/wt}$)=4, n(A+/$P^{ki/ki}$)=6. B) Insoluble, but not soluble Aβ40 and -42 levels are slightly, but not significantly lower in the hippocampus of the A+/$P^{ki/ki}$ mice as compared to the A+/$P^{wt/wt}$ mice. Aβ40 and -42 levels are normalized to the total protein concentration in the same sample. n(A+/$P^{wt/wt}$)=5, n(A+/$P^{ki/ki}$)=6. C) Representative Western blots and corresponding quantification show no differences in the levels of full-length APP (APPtot, normalized to GAPDH), APP C-terminal fragments (C99 and C83, normalized to APPtot), or soluble APPα and –β (sAPPα, sAPPβ, normalized to APPtot) species in the hippocampus of the A+/$P^{ki/ki}$ and the A+/$P^{wt/wt}$ mice. n(A+/$P^{wt/wt}$)=4, n(A+/$P^{ki/ki}$)=4. Scale bars in the representative immunofluorescent images are 657 μm for the whole area and 164 μm for the zoomed view. Unpaired t-test. All data are presented as mean ± SEM. Each datapoint represents an individual mouse.

A)

Hippocampus, 13-month-old female mice

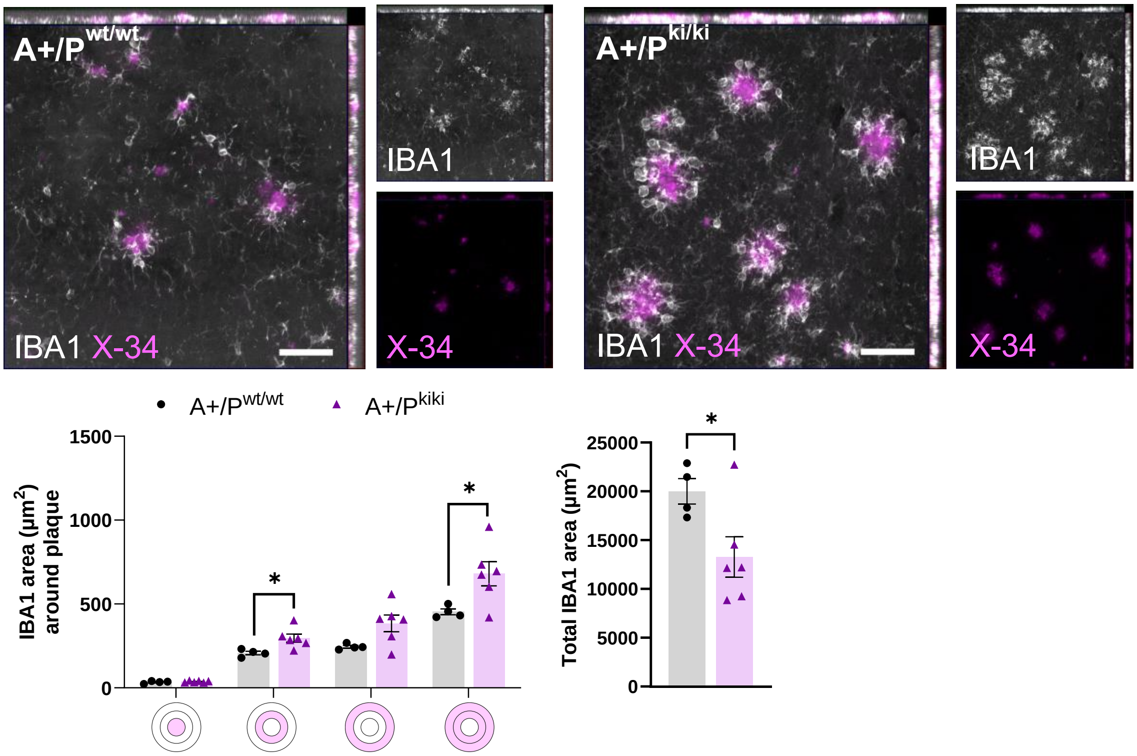

B)

Hippocampus, 13-month-old female mice

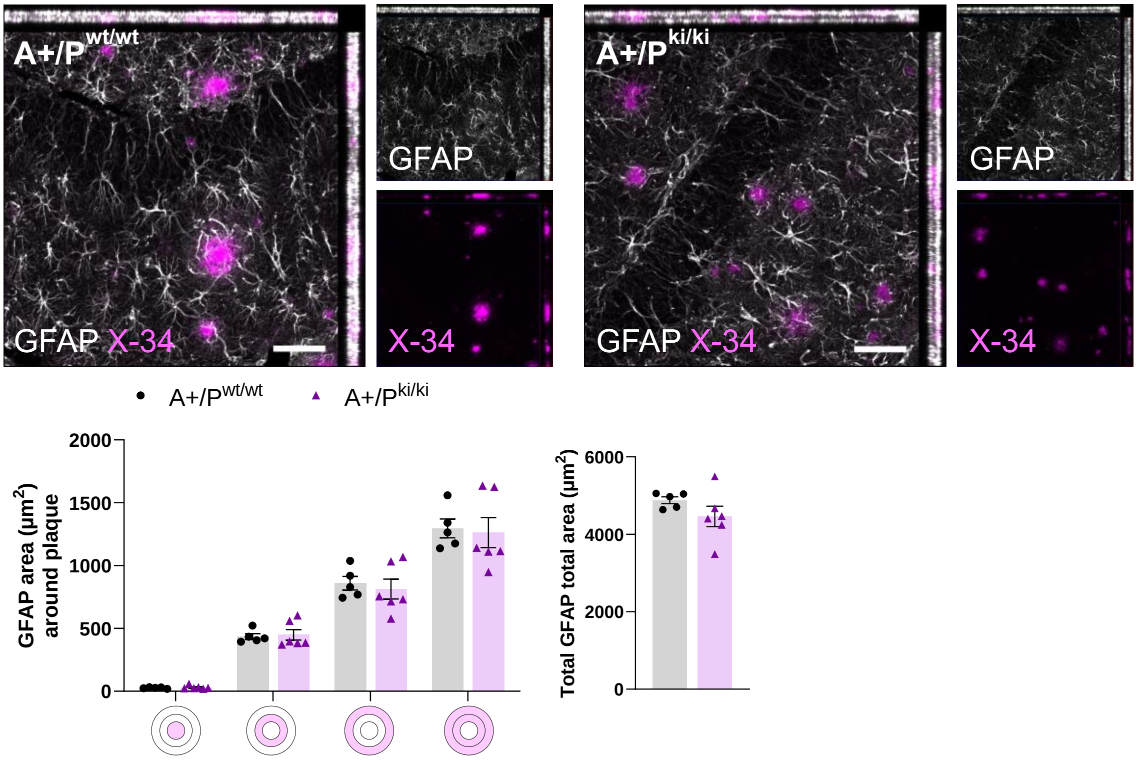

Supplementary Figure 3. PLCγ2-P522R variant increases microglia clustering around β-amyloid plaques in the hippocampus of female APP/PS1 mice. A) Area (μm^2) of IBA1-positive microglia is increased within 0-20 μm (*p=0.023) and 0-40 μm (*p=0.037) from the plaque outline in the hippocampus of the A+/$P^{ki/ki}$ mice as compared to the A+/$P^{wt/wt}$ mice. Simultaneously, total IBA1 area is decreased (*p=0.043). n(A+/$P^{wt/wt}$)=4, n(A+/$P^{ki/ki}$)=6. B) GFAP-positive astrocyte area (μm^2) around plaques (within 0-20 and 20-40 μm from the plaque outline) and total GFAP area remain unaltered between the genotypes. n(A+/$P^{wt/wt}$)=5, n(A+/$P^{ki/ki}$)=6. The scale bar in the representative immunofluorescent images is 50 μm. Unpaired samples t-test. All data are presented as mean ± SEM. Each datapoint represents an individual mouse.

Hippocampus, 13-month-old female mice

A)

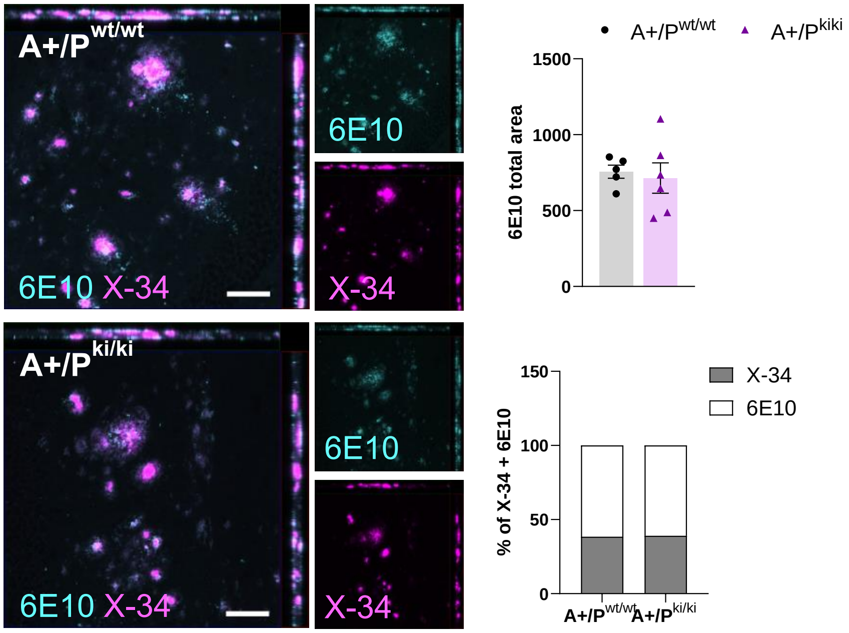

Hippocampus, 13-month-old female mice

B)

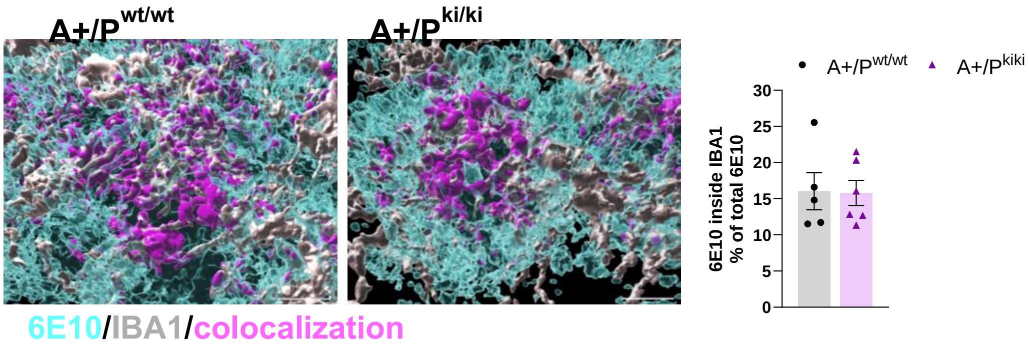

Hippocampus, 13-month-old female mice

C)

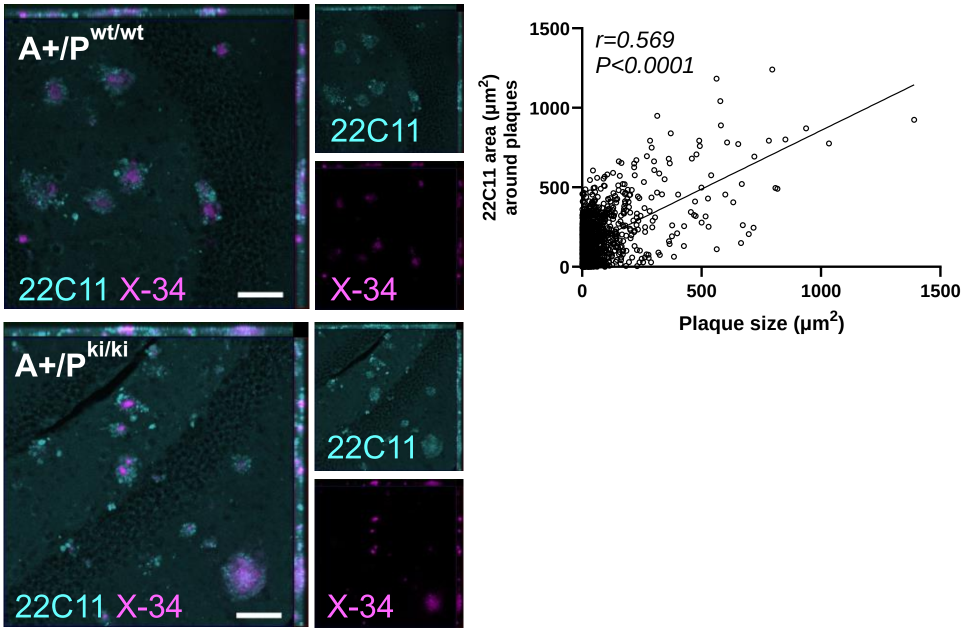

Supplementary Figure 4. PLCγ2-P522R variant decreases dystrophic neurites around β-amyloid plaques in the hippocampus of female APP/PS1 mice. A) Total area (μm^2) of diffuse Aβ (6E10) and composition β-amyloid plaques, as indicated by percentage of compact amyloid (X-34) of all β-amyloid (X-34+6E10), remain unaltered in the hippocampus of the APP/PS1xPlγ2-P522R (A+/P$^{ki/ki}$) mice as compared to the APP/PS1 (A+/P$^{wt/wt}$) mice. n(A+/P$^{wt/wt}$)=5, n(A+/P$^{ki/ki}$)=6. B) A 3D-reconstruction of 6E10 and IBA1 (microglia) signal and their co-localization and quantification showing 6E10 within IBA1 as % of all 6E10 in the hippocampus of A+/P$^{ki/ki}$ mice as compared to the A+/P$^{wt/wt}$ mice. C) Area (μm^2) of 22C11-labeled dystrophic neurites around β-amyloid plaques strongly correlates with plaque size (r=0.569, p<0.0001). 22C11 area is decreased within 0-7 μm (*p=0.01) and 0-14 μm (*p=0.01) distance from the β-amyloid plaque outline in the hippocampus of the A+/P$^{ki/ki}$ mice as compared to the A+/P$^{wt/wt}$ mice when normalized to the plaque size. n(A+/P$^{wt/wt}$)=4, n(A+/P$^{ki/ki}$)=6. The scale bar in the representative immunofluorescent images is 50 μm. Unpaired t-test and Pearson correlation. All data are presented as mean ± SEM. Each datapoint represents an individual mouse.

A) **Temporo-occipital cortex, 13-month-old female mice**

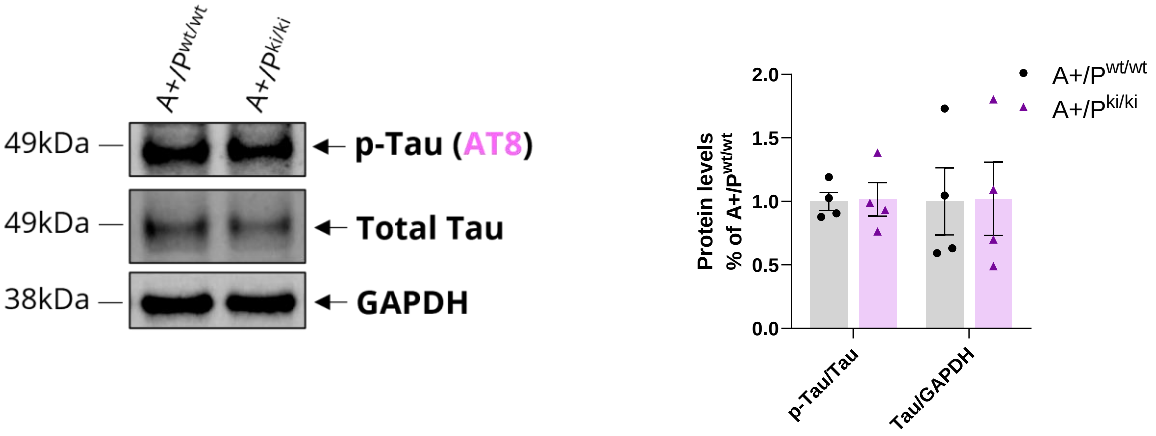

B) **Hippocampus, 13-month-old female mice**

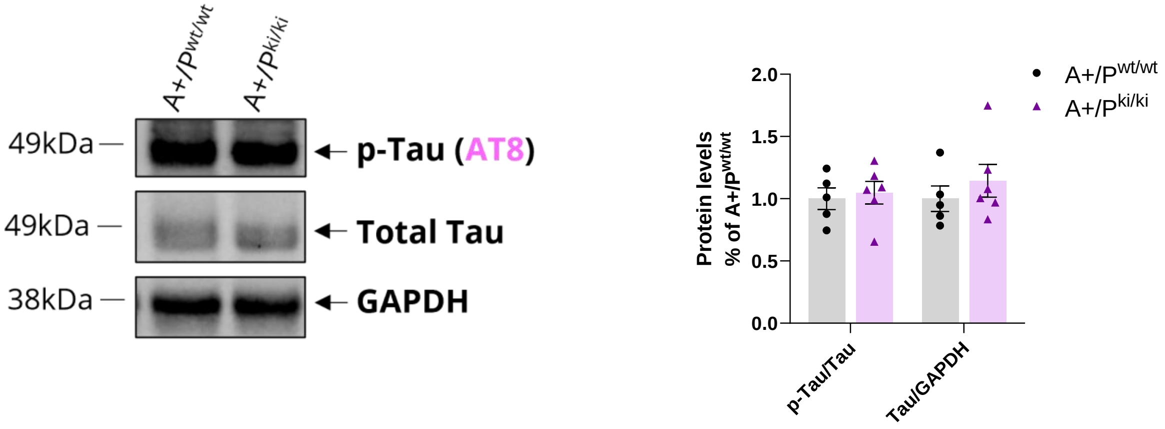

Supplementary Figure 5. PLCγ2-P522R variant does not affect the ratio of p-Tau/total Tau in the temporo-occipital cortex or hippocampus of female APP/PS1 mice. Representative Western blots and corresponding quantification did not show statistically significant differences in the ratio of p-Tau/total Tau or total Tau normalized to GAPDH A) in the temporo-occipital cortex of the A+/$P^{ki/ki}$ and the A+/$P^{wt/wt}$ mice, n(A+/$P^{wt/wt}$)=4, n(A+/$P^{ki/ki}$)=4 and B) in the hippocampus of the A+/$P^{ki/ki}$ and the A+/$P^{wt/wt}$ mice, n(A+/$P^{wt/wt}$)=5, n(A+/$P^{ki/ki}$)=6). Unpaired t-test. All data are presented as mean ± SEM. Each datapoint represents an individual mouse.

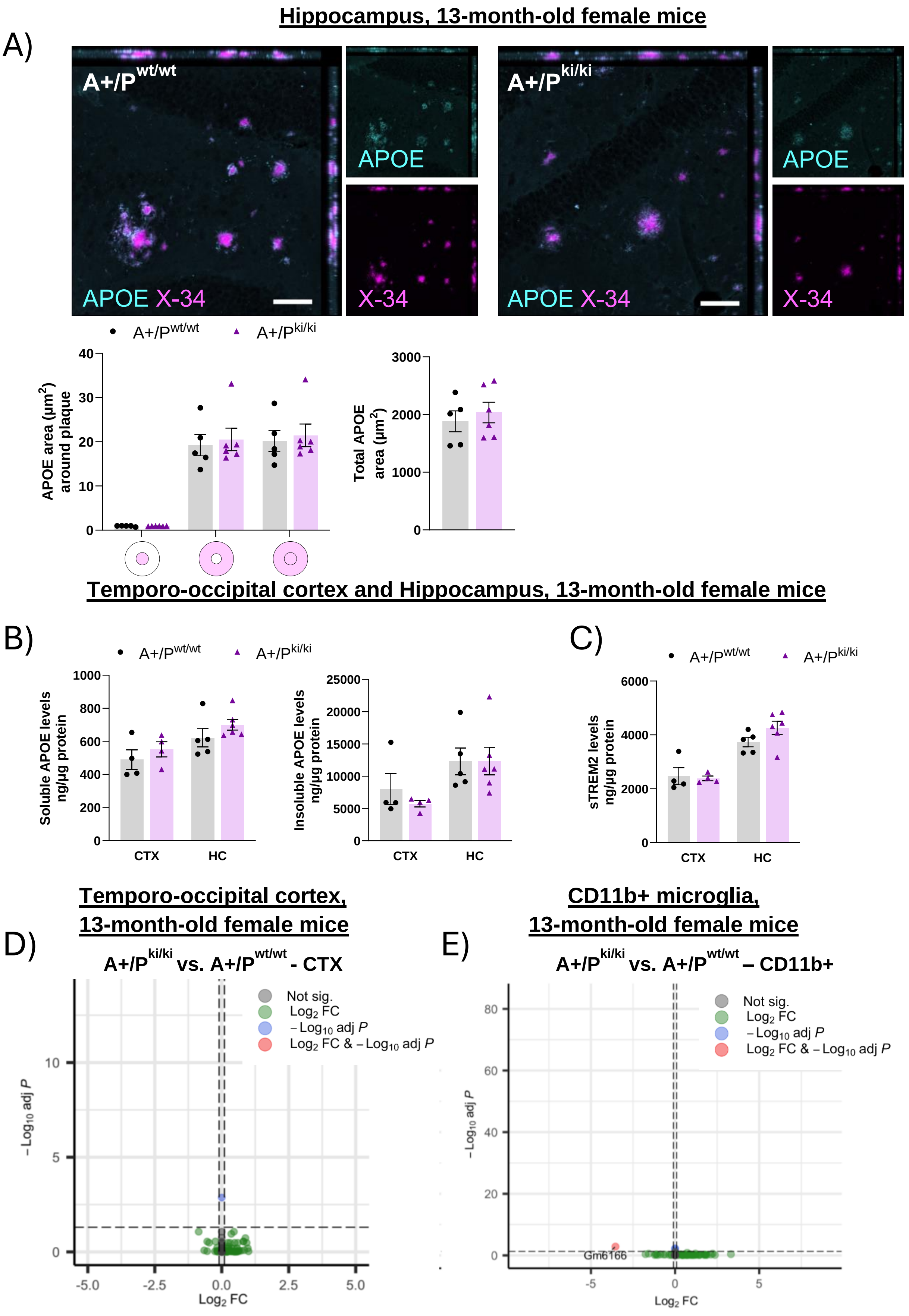

Hippocampus, 13-month-old female mice
A)
A+/Pwt/wt
APOE
APOE X-34
X-34
A+/Pki/ki
APOE
APOE X-34
X-34
A+/Pwt/wt
A+/Pki/ki
APOE area (μm2) around plaque
Total APOE area (μm2)
Temporo-occipital cortex and Hippocampus, 13-month-old female mice
B)
A+/Pwt/wt
A+/Pki/ki
Soluble APOE levels ng/μg protein
Insoluble APOE levels ng/μg protein
CTX
HC
C)
A+/Pwt/wt
A+/Pki/ki
sTREM2 levels ng/μg protein
CTX
HC
Temporo-occipital cortex, 13-month-old female mice
D)
A+/Pki/ki vs. A+/Pwt/wt - CTX
Not sig.
Log2 FC
–Log10 adj P
Log2 FC & –Log10 adj P
–Log10 adj P
Log2 FC
CD11b+ microglia, 13-month-old female mice
E)
A+/Pki/ki vs. A+/Pwt/wt – CD11b+
Not sig.
Log2 FC
–Log10 adj P
Log2 FC & –Log10 adj P
Gm6166
–Log10 adj P
Log2 FC

Supplementary Figure 6. PLCγ2-P522R variant does not change plaque-associated APOE in the hippocampus of female APP/PS1 mice. A) Analysis of total APOE or APOE area (μm^2) on top and surrounding (within 0-10 and 10-20 μm from the β-amyloid plaque outline) β-amyloid plaques reveals no differences between the APP/PS1xPlγ2-P522R (A+/P$^{ki/ki}$) and APP/PS1 (A+/P$^{wt/wt}$) mice. n(A+/P$^{wt/wt}$)=5, n(A+/P$^{ki/ki}$)=6. The scale bar in the representative immunofluorescent images is 50 μm. Unpaired samples t-test. All data are presented as mean ± SEM. Each datapoint represents an individual mouse. B) Volcano blot showing differentially expressed genes (DEGs) in parieto-occipital cortex and C) CD11b+ microglia isolated from A+/P$^{wt/wt}$ and A+/P$^{ki/ki}$ mouse brain.

<u>**CD11b+ microglia, 13-month-old female mice**</u>

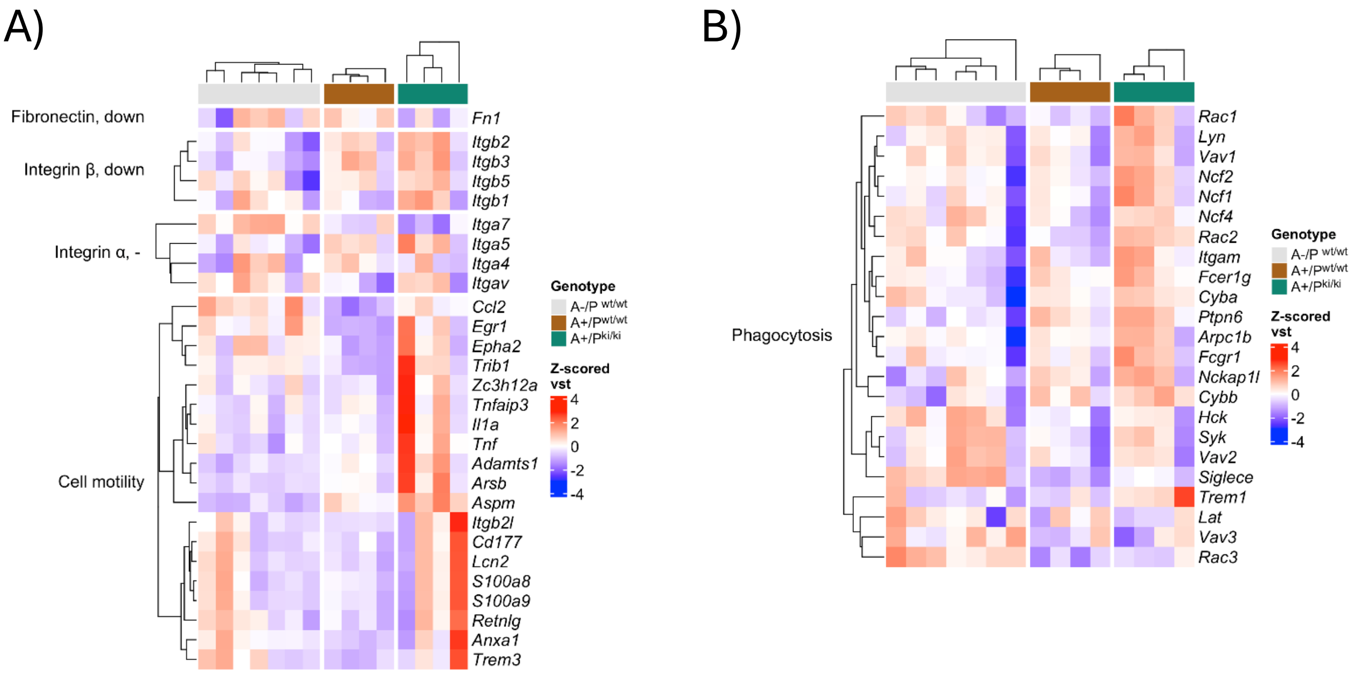

Supplementary Figure 7. The expression profiles of PLCγ2-associated integrins, and cell motility and phagocytosis genes in CD11b+ microglia isolated from female PLCγ2-P522R KI and WT mice. Heatmaps of z-scored vst-normalized gene expression in CD11b+ microglia isolated from the whole brain of 13-month-old wildtype (A-/P$^{wt/wt}$), A+/P$^{wt/wt}$ and A+/P$^{ki/ki}$ female mice for A) PLCγ2-associated genes (Fibronectin and Integrin classes α and β) and GSEA core enrichment genes of the GOBP_CELL_MOTILITY gene set (Cell motility) from the comparison of A+/Pwt/wt and A+/P$^{ki/ki}$ microglia, and B) select genes in the WP_MICROGLIA_PATHOGEN_PHAGOCYTOSIS_PATHWAY. CD11b+ A-/P$^{wt/wt}$ n=7, A+/P$^{wt/wt}$ n=4, and A+/P$^{ki/ki}$ n=4.

CD11b+ microglia, 13-month-old male mice

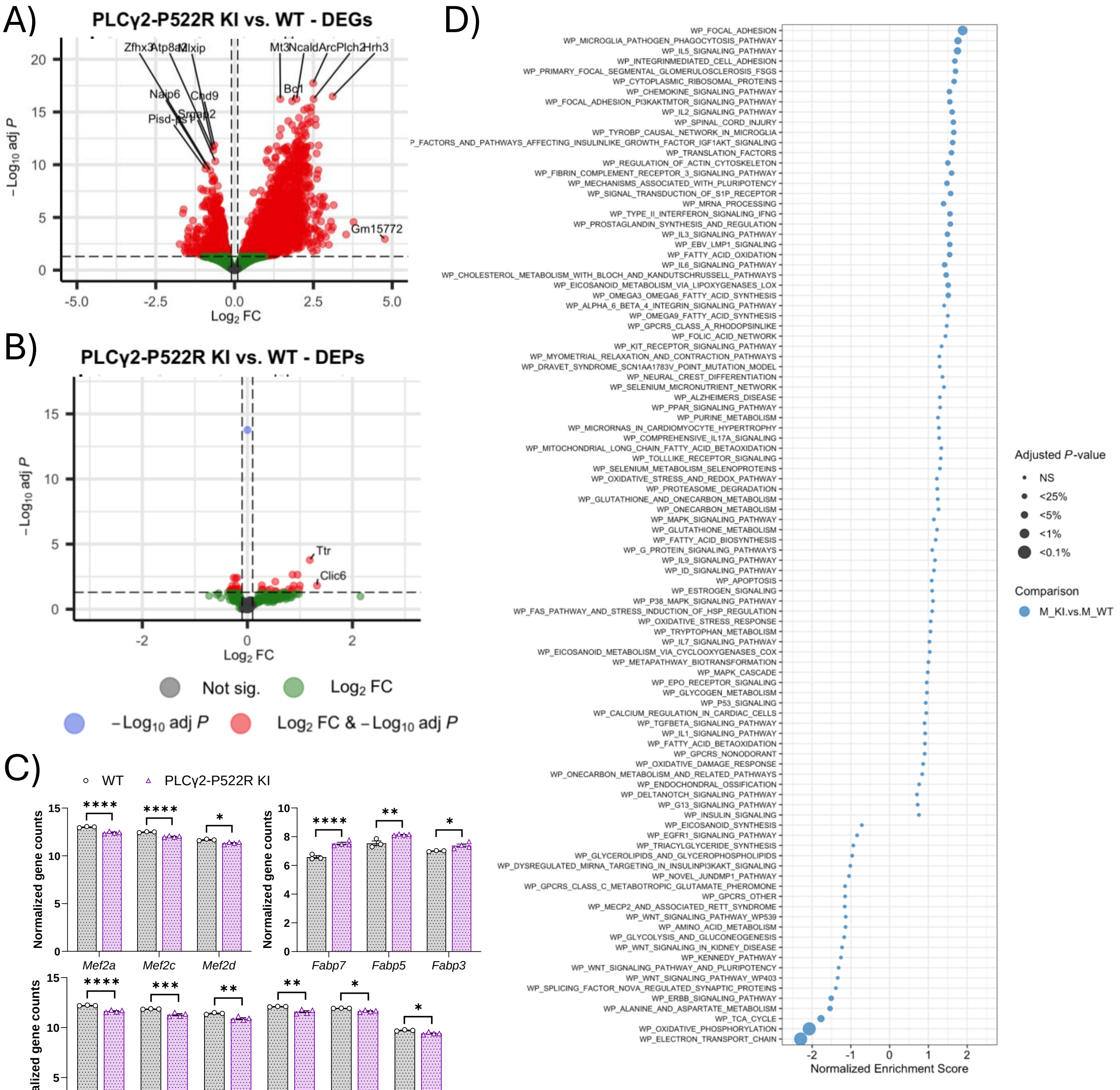

CD11b+ microglia, 13-month-old female mice

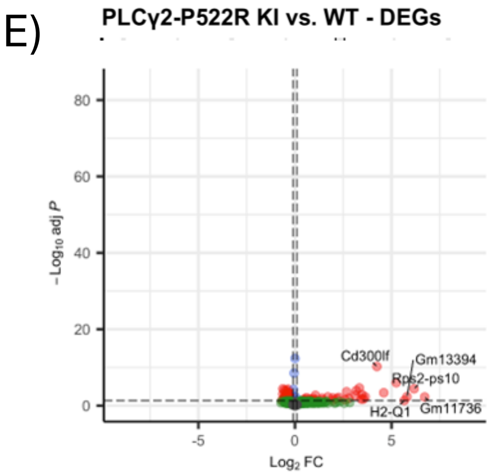

Supplementary Figure 8. Differentially expressed genes and proteins in CD11b+ microglia of WT and PLCγ2-P522R KI male mice. Volcano plot of A) differentially expressed genes (DEGs) and B) differentially expressed proteins (DEPs, expressed as their encoding gene symbols) in CD11b+ microglia isolated from 13-month-old PLCγ2-P522R KI and WT male mice. FDR<0.05. Horizontal dashed line: adjusted p-value 0.05; vertical dashed lines: |log2FC|=0.1. C) PLCγ2-P522R downregulates calcium-sensitive transcription family members, *Mef2a* (****pAdj<0.0001), *Mef2c* (****pAdj<0.0001), and *Mef2d* (*pAdj=0.015) as compared to WT microglia. Expression of genes encoding fatty acid binding proteins *Fabp7* (****pAdj<0.0001), *Fabp5* (pAdj=0.003), and *Fabp3* (*pAdj=0.049) is increased in PLCγ2-P522R KI microglia. Expression of several genes encoding phosphatidylinositol 3-kinase (PI3K) subunits, *Pik3cd* (****pAdj<0.0001), *Pik3cg* (***pAdj=0.0003), *Pik3r5* (**pAdj=0.002), *Pik3ap1* (**pAdj=0.003), *Pik3r1* (*pAdj=0.012), *Pik3r4* (*pAdj=0.033), is downregulated in PLCγ2-P522R KI as compared to WT microglia. D) A dot plot of normalized enrichment scores for enriched and depleted protein sets in enrichment analyses by GSEA for protein (Wikipathways) expression in PLCγ2-P522R KI and WT mice. E) Volcano plot of differentially expressed genes (DEGs) in CD11b+ microglia isolated from 13-month-old female PLCγ2-P522R KI and WT mice. RNA n(WT)= male 3 and female 7, n (PLCγ2-P522R KI) = male 4 and female 4, protein n(WT)=5, n(PLCγ2-P522R KI)=6.

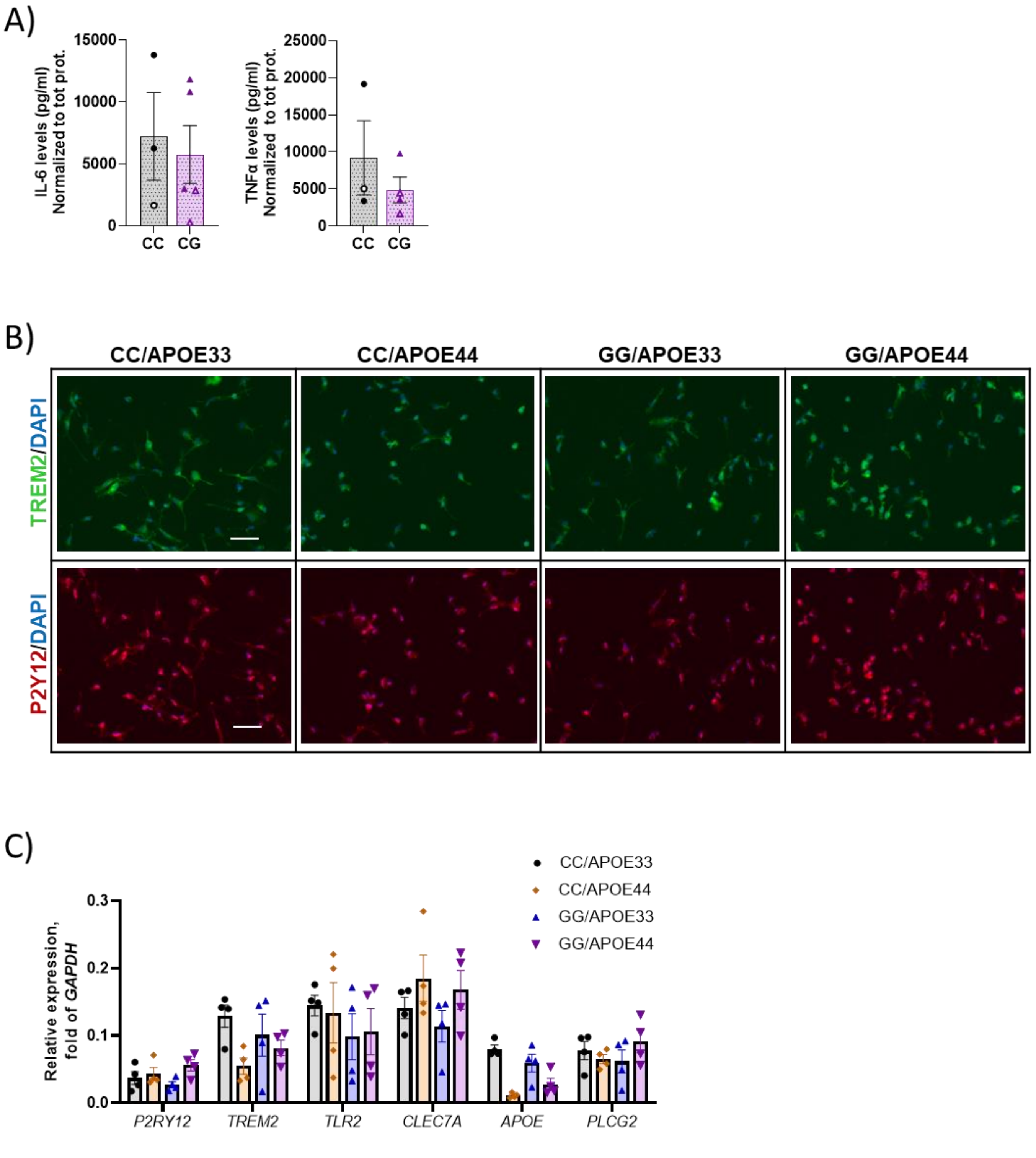

Supplementary Figure 9. Characterization of human microglia-like cell models. A) IL-6 and TNF-α levels in the conditioned medium of blood monocyte-derived microglia (MDMi) of the PLCγ2-P522R variant carriers (CG) and matched controls (CC) after treatment with

lipopolysaccharide for 24 h. IL-6 and TNF-α levels are normalized to the total protein concentration in the respective lysate. n(CC)=3, n(CG)=4. Each datapoint represents data from one individual. Colored circles indicate data obtained from females and hollow circles data obtained from males. Microglial markers in induced pluripotent stem cell-derived microglia (iMGL) detected by immunocytochemistry (B) and RT-qPCR (C). In (B) scale bars are 50μm. Data are from PLCγ2 control line (CC) and isogenic PLCγ2-P522R homozygous line (GG) with either *APOE33* or *APOE44* background. In (C) there were two batches of cells, two technical replicates each. All data are presented as mean ± SEM.
