# Supplementary material for: The protective PLCγ2-P522R variant mitigates Alzheimer’s disease-associated pathologies by enhancing beneficial microglial functions
Source: J Neuroinflammation. 2025 Mar 5;22:64. doi: 10.1186/s12974-025-03387-6 (PMC11881468; doi:10.1186/s12974-025-03387-6)
Supplement: Supplementary file 4 — Additional file 4 [file 12974_2025_3387_MOESM4_ESM.docx]

**SUPPLEMENTARY MATERIALS AND METHODS**

**Western blotting of Tau protein**

Primary antibodies used for Western blotting of phosphorylated Tau (p-Tau), total Tau, and GAPDH were mouse anti-Phospho-Tau 1:1000 (AT8; Ser202, Thr205; #MN1020, Invitrogen), mouse anti-Tau 1:1000 (4-repeat isoform RD4, #05-804, Millipore), and mouse anti-GAPDH 1:10 000 (#ab8245, Abcam), respectively.

**IL-6 and TNF-α measurement in conditioned MDMi medium**

For analyzing inflammatory cytokines in the conditioned medium, medium was collected, centrifuged at 10 000 x g, for 10 min at +4 °C, and supernatant was stored at –80 °C until analysis (below). IL-6 and TNF-α levels in the medium were analyzed with IL6 and TNF alpha Human Uncoated ELISA Kit (#88-7346-22 and #88-7066-22, respectively Thermo Fisher).

**Immunocytochemistry and qPCR-based characterization of microglia markers in iMGL cells**

iMGL cells grown on coverslips coated with Matrigel (1:100) were fixed with 4% formaldehyde for 20 min at RT and stained overnight with rabbit anti-P2Y12 (1:1000, #HPA014518, Merc) and goat anti-TREM2 (1:100 #AF1828, R&D Systems) primary antibodies diluted in PBS containing 1% BSA and 0.05% Tween20 at +4 °C. After 4 x 5min washing in PBS + 0.05% Tween20, the cells were incubated for 1 h with chicken anti-rabbit AlexaFluor 647 (#A21443, ThermoFisher Scientific) and chicken anti-goat AlexaFluor488 (#A21467, ThermoFisher Scientific) secondary antibodies (both diluted 1:500 in PBS containing 1% BSA and 0.05% Tween20) at RT. During the washing step, the cells were further incubated for 5 min with DAPI at 1 μg/ml in PBS to stain the nuclei. The coverslips were mounted on glass slides using Fluoromount-G mounting medium (#0100-01, Southern Biotech), and imaged using Zeiss AxioImager M1 microscope.

iMGL cells for qPCR-based characterization were grown and maturated for 4-5 days on 12-well plates coated with Matrigel (2:100). RNA was isolated by using Qiagen RNeasy Mini Kit (#74106, Qiagen) according to manufacturer’s instructions. cDNA was synthetized using Maxima reverse transcriptase enzyme (#EP0742, Thermo Fisher Scientific) according to manufacturer’s instructions. RT-qPCR was used to analyze the expression levels of microglia genes, *P2RY12, TREM2, TLRM2, CLEC7A, APOE,* and *PLCG2* by using Maxima Probe/ROX qPCR Master Mix (#11813923, Thermo Fisher Scientific) and the Taqman primers listed in **Supplementary table 1** on Bio-Rad CFX96 Real-Time System (Biorad)**.** The relative mRNA expression results were normalized to GAPDH using Q-gene program (DOI: [10.1093/bioinformatics/btg157](https://doi.org/10.1093/bioinformatics/btg157) ).

**Table 1:** Primers used for characterization of microglia markers in iMGL cells.

| **Primers** | **Producer** | **Catalog number** |
| --- | --- | --- |
| P2RY12 | Thermo Fisher Scientific | Hs00375457_m1 |
| TREM2 | Thermo Fisher Scientific | Hs00219132_m1 |
| TLR2 | Thermo Fisher Scientific | Hs00152932_m1 |
| CLEC7A | Thermo Fisher Scientific | Hs00224028_m1 |
| APOE | Thermo Fisher Scientific | Hs00171168_m1 |
| PLCG2 | Thermo Fisher Scientific | Hs00182192_m1 |
| GAPDH | Thermo Fisher Scientific | Hs99999905_m1 |
